# Product‐Reshaped Gas‐Phase Hydrodynamics Govern CO_2_ Electrolysis in Gas Diffusion Electrodes

**DOI:** 10.1002/advs.78274

**Published:** 2026-10-14

**Authors:** Abdullah A. Alyahya, Mayra S. Tovar‐Oliva, Blessings G. Malimusi, Sebastián Murcia‐López, María Escudero‐Escribano, Timm Krüger, Ignacio Tudela

**Affiliations:** ^1^ School of Engineering, Institute for Materials and Processes, Edinburgh Electrochemical Engineering Group (e3 Group) The University of Edinburgh Edinburgh United Kingdom; ^2^ Centre of Excellence for Nanomaterials for Clean Energy Applications King Abdulaziz City for Science and Technology (KACST) Riyadh Saudi Arabia; ^3^ Catalan Institute of Nanoscience and Nanotechnology (ICN2) Edifici ICN2, CSIC and Barcelona Institute of Science and Technology, UAB Campus Bellaterra Barcelona Spain; ^4^ Catalan Institution for Research and Advanced Studies (ICREA) Barcelona Spain; ^5^ School of Engineering, Institute for Multiscale Thermofluidics The University of Edinburgh Edinburgh United Kingdom

**Keywords:** continuum modeling, $C_{2+}$ products, ${\mathrm{CO}}_{2}$ electroreduction, gas diffusion electrodes, gas‐phase hydrodynamics, intermediate availability, reactor scale‐up

## Abstract

High‐rate ${\mathrm{CO}}_{2}$ electrolysis is commonly assessed using current density, single‐pass conversion and Faradaic efficiency, but these global metrics do not indicate whether the local reaction environment required for selective multicarbon formation is preserved during scale‐up. Using an experimentally validated, flow‐direction‐resolved continuum model of a Cu‐based gas‐diffusion‐electrode (GDE) flow‐cell system, we demonstrate that gas‐fed ${\mathrm{CO}}_{2}$ electrolysers behave as reacting‐flow electrochemical reactors in which electrochemical conversion transforms the gas phase in the cathode compartment and makes it part of the reactor response. As cathode potential becomes more negative, ${\mathrm{CO}}_{2}$ consumption, CO formation and consumption, and gaseous product evolution progressively dilute the ${\mathrm{CO}}_{2}$‐rich reacting gas phase, altering gas composition, density and velocity and feeding back into reactant delivery and intermediate availability. This reaction‐induced hydrodynamic response creates an operating window favoring ethylene formation and reveals a conservation‐driven feedback that becomes unavoidable during scale‐up: as electrode length, conversion and gaseous product formation increase, extended electrodes must preserve the spatial overlap between ${\mathrm{CO}}_{2}$ delivery, CO generation, controlled CO consumption and limited dilution of the reacting gas phase.

## Graphical abstract

In gas‐fed ${\mathrm{CO}}_{2}$ electrolysers, the gas phase is part of the electrochemical response. As gaseous products form, they change gas composition and flow, which in turn affects ${\mathrm{CO}}_{2}$ delivery and CO intermediate availability. This feedback changes the reaction environment needed for multicarbon formation, becoming unavoidable as reactors are scaled to larger areas, higher conversion, and greater gas production.

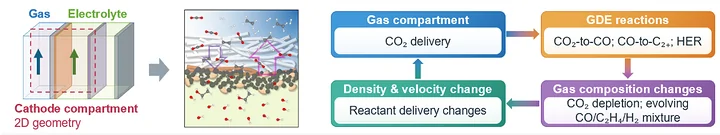

## 1 Introduction

The electrochemical reduction of ${\mathrm{CO}}_{2}$ offers a route to convert renewable electricity and captured carbon into chemical feedstocks and fuels [1, 2, 3]. Among the possible products, multicarbon hydrocarbons and oxygenates are particularly attractive because they increase the value of the carbon stream, but their formation requires control over complex reaction networks and local interfacial environments [4, 5]. Cu‐based catalysts remain the main platform for forming $C_{2+}$ products, although their selectivity is highly sensitive to catalyst structure, electrolyte composition, reactant transport and operating conditions [4, 6, 7].

Flow reactors equipped with gas diffusion electrodes (GDEs) have enabled much higher current densities than conventional H‐cells by supplying gaseous ${\mathrm{CO}}_{2}$ directly to the catalyst layer, thereby mitigating limitations associated with the low solubility of ${\mathrm{CO}}_{2}$ in aqueous electrolytes [8]. This shift was partly motivated by a better understanding of how catalyst activity and selectivity depend on the local reaction environment, which differs significantly between low‐current cells and commercially relevant GDE operation [9]. However, GDE operation also introduces coupled transport and phase‐distribution effects that become increasingly important during scale‐up. A GDE is not a passive ${\mathrm{CO}}_{2}$ delivery layer; it is a porous multiphase structure in which gas access, electrolyte penetration, wettability, ionic conduction, and product removal must remain balanced [10, 11].

Most studies of gas‐fed ${\mathrm{CO}}_{2}$ electroreduction have focused on catalyst identity, local reaction conditions, membrane configuration or cell design, and have generally used global metrics, such as current density, Faradaic efficiency and single‐pass conversion to evaluate overall process performance [4, 10]. These design variables and performance indicators remain essential, but they do not by themselves explain how a gas‐fed electrode behaves as it is scaled in the flow direction. From an electrochemical reactor‐engineering perspective, process scale‐up is a coupled reactor problem in which current distribution, mass transfer, hydrodynamics and geometry must be considered together rather than through global performance metrics alone [12]. Recent stack‐level analysis of CO and ${\mathrm{CO}}_{2}$ electrolysis has further emphasized that pressure and concentration gradients, flow distribution and flow‐channel design become increasingly important at scale [13]. The remaining challenge is to understand how the gas phase itself evolves when ${\mathrm{CO}}_{2}$ consumption, CO formation and consumption, and gaseous product generation occur simultaneously along a gas‐fed GDE.

Continuum models are useful for addressing this challenge because local reaction environments are difficult to probe experimentally inside operating GDE reactors. They can link measured performance to spatial distributions of flow, composition, potential and current density that are not accessible from global current density or product analysis alone [14]. Previous continuum descriptions have provided important insight into ${\mathrm{CO}}_{2}$ transport limitations, current and potential distributions, local pH, electrolyte chemistry, crossover and spatial non‐uniformity in ${\mathrm{CO}}_{2}$ electrolysers [15, 16, 17, 18, 19, 20, 21]. For example, validated one‐dimensional (1D) continuum models for MEA systems have shown how ${\mathrm{CO}}_{2}$‐to‐CO performance and crossover emerge from coupled ion formation, transport, ${\mathrm{CO}}_{2}$ solubility, and water management [21], while two‐dimensional (2D) GDE models have shown that along‐channel depletion of ${\mathrm{CO}}_{2}$ and buffer species can cause strong spatial variations in current density at high single‐pass conversion [18].

These studies establish that local reaction environments cannot be inferred directly from inlet conditions or global performance metrics. However, most continuum models have been developed around questions of catalyst, electrolyte, membrane and interfacial control of activity and selectivity. As gas‐fed ${\mathrm{CO}}_{2}$ electrolysis moves toward larger electrode areas and stack‐relevant operation, an important electrochemical reactor‐engineering question is whether the reactor can preserve the local reacting environment required for selectivity along the flow direction. Answering this question requires treating the cathode gas phase not as a fixed reservoir imposed by the inlet composition, but as a reacting‐flow domain whose composition, density and velocity evolve with electrochemical conversion.

Within this reacting‐flow problem, CO provides the key intermediate link between ${\mathrm{CO}}_{2}$ delivery and $C_{2+}$ formation on Cu‐based catalysts [22, 23, 24, 25]. The local rate of $C_{2+}$ formation therefore depends not only on intrinsic reaction kinetics, but also on how rapidly ${\mathrm{CO}}_{2}$ is supplied, how much CO is generated, and whether this CO remains locally available or is transported away. At the same time, CO availability does not by itself determine product branching among ethylene, ethanol, and other CO‐derived products, which also depends on product‐specific kinetic factors and downstream surface chemistry [5, 17].

Here, we develop and validate a 2D cathode‐side continuum model for multi‐product ${\mathrm{CO}}_{2}$ electroreduction in a flow reactor equipped with a GDE containing Cu‐based catalysts. The model resolves the coupled gas, liquid, and electrochemical processes that determine local ${\mathrm{CO}}_{2}$ delivery, CO formation and consumption, electrolyte speciation, hydrogen evolution and gaseous product transport along the electrode. Model parameterization and validation were based on the same experimental platform used to define the simulated reactor, for which geometry, flow conditions, electrolyte composition, GDE structure, and product‐current data were available [11]. This provides a controlled validation context in which the ${\mathrm{CO}}_{2}$‐to‐CO transition and HER response can be interpreted quantitatively, while deviations among individual CO‐derived products identify the boundary between reactor‐scale intermediate availability and product‐specific surface chemistry. The model is then used to examine how reaction‐induced changes in gas‐phase hydrodynamics couple local ${\mathrm{CO}}_{2}$ utilization, CO availability and multicarbon product formation along the electrode length. The simplified cell architecture, coordinate system, and corresponding 2D model section are shown in Figure 1.

**FIGURE 1:**
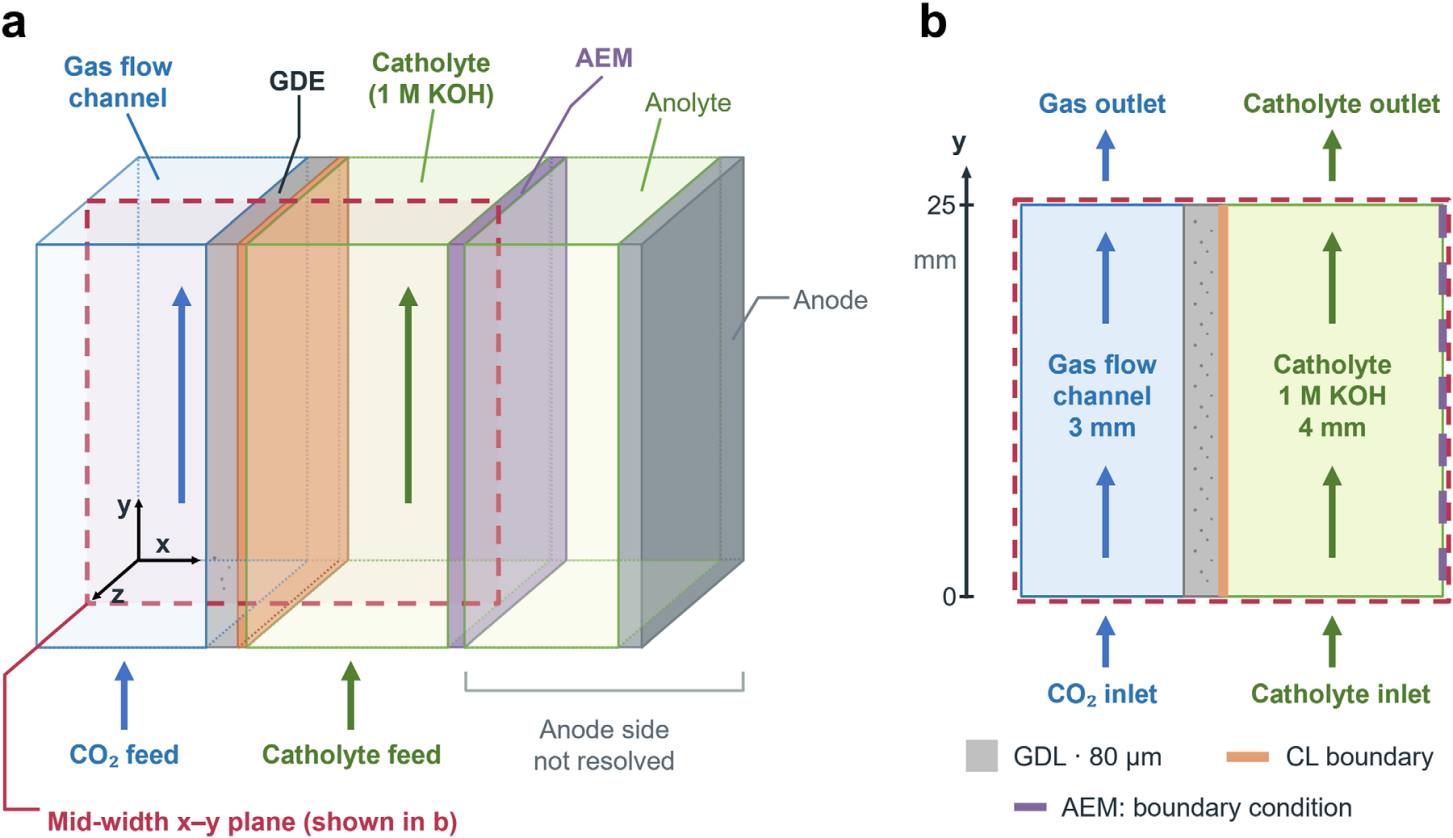
Simplified cell architecture and two‐dimensional cathode‐side model. (a) The cell is arranged in the through‐plane direction (x), with the gas and electrolyte flow channels extending along the electrode length (y) and the electrode width defined along z. The GDE separates the gas flow channel from the catholyte channel, while the AEM and anode‐side compartment are shown for context. The highlighted plane indicates the mid‐width x–y section represented by the model. (b) Corresponding 2D computational domain, including the gas flow channel, the 80μm porous gas diffusion layer (GDL), the catalyst‐layer (CL) reactive boundary and the electrolyte flow channel. The electrolyte‐facing surface of the AEM is represented through boundary conditions. Gas and catholyte enter at y=0 and flow along the 25 mm electrode length. The model resolves variations in the x–y plane while assuming uniformity across the electrode width (z). Schematic not to scale; thin layers are enlarged for clarity.

## 2 Results

### 2.1 Product Formation Turns the Gas Phase into a Dynamic Transport Domain

Product selectivity is often one of the first metrics used to evaluate electrochemical reactors, including ${\mathrm{CO}}_{2}$ electrolysers. During scale‐up, however, selectivity must be interpreted together with the transport environment that supplies reactants to the GDE. If gaseous products alter local composition, density and velocity, the gas phase cannot be treated as a fixed ${\mathrm{CO}}_{2}$ supply boundary imposed only by the inlet flow rate. Figure 2 addresses this point by showing how product formation changes the gas‐flow and composition fields as the cathode potential becomes more negative. The electrolyte velocity field remains essentially unchanged across the simulated potentials because the electrolyte was treated as an incompressible single‐phase liquid with constant density and viscosity (Figure 2 a). In contrast, the gas velocity increases along the gas flow channel as the cathode potential becomes more negative, showing that the gas phase responds to electrochemical conversion rather than acting only as a passive ${\mathrm{CO}}_{2}$ delivery channel (Figure 2a,b).

**FIGURE 2:**
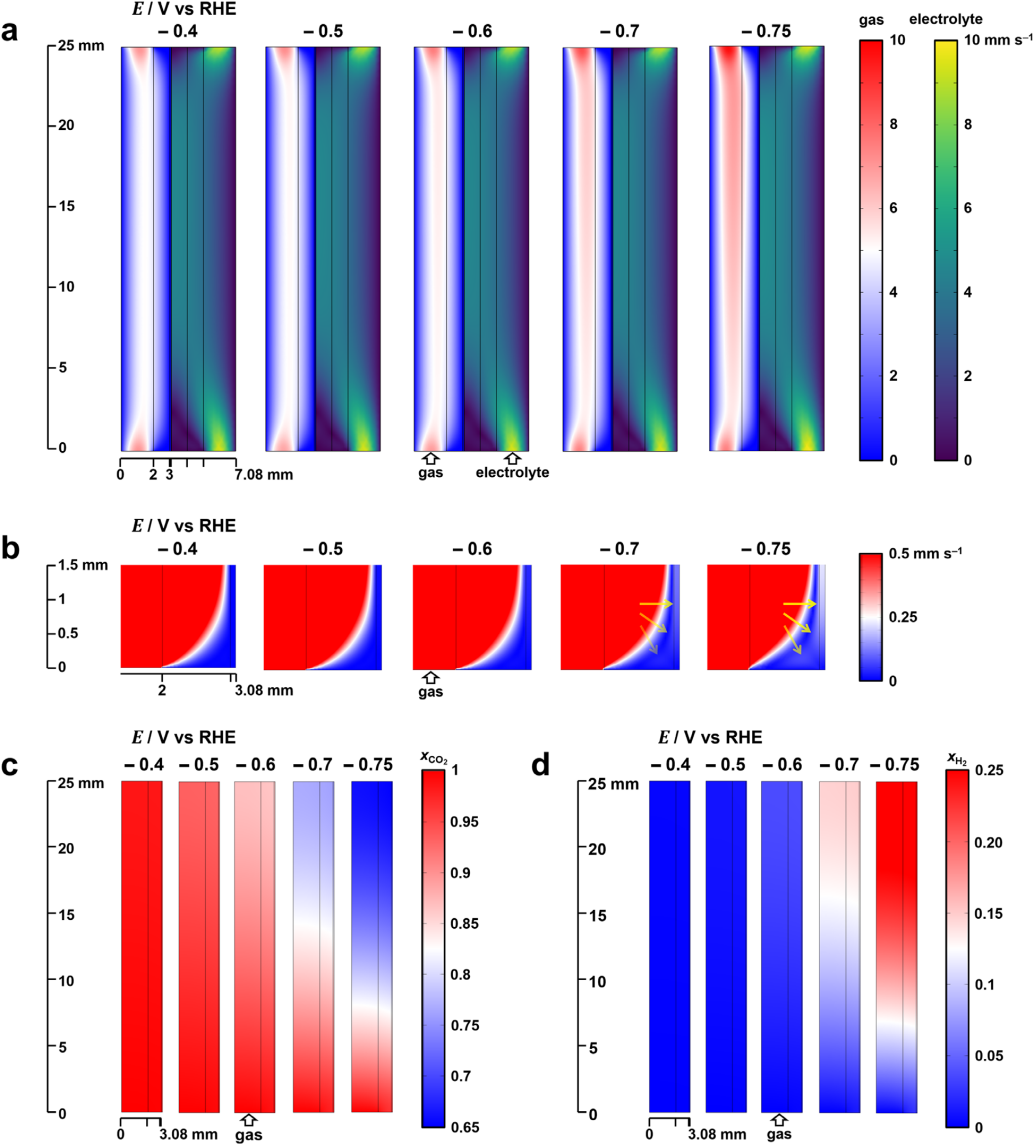
Product formation reshapes gas transport in the cathode compartment of the GDE flow reactor. (a) Simulated velocity magnitude in the full cathode compartment; in the porous GDE, the plotted velocity corresponds to the superficial gas velocity. (b) Magnified velocity field near the gas inlet, showing the increasingly dynamic region adjacent to the GDE as the cathode potential becomes more negative. Yellow arrows are visual guides highlighting the stronger near‐interface gas‐velocity changes at high overpotential. (c,d) Gas‐phase molar fractions of ${\mathrm{CO}}_{2}$ and $H_{2}$, respectively, showing progressive ${\mathrm{CO}}_{2}$ depletion and increasing $H_{2}$ accumulation along the gas flow direction. All cathode potentials are reported versus the reversible hydrogen electrode (RHE). The gas phase therefore evolves with electrochemical conversion through coupled changes in composition, density and velocity.

The increase in gas velocity arises from changes in gas composition (Figure 2c,d; Figures S4, S5 and S6). At mild cathodic potentials, the gas phase remains dominated by ${\mathrm{CO}}_{2}$. As the overpotential increases, ${\mathrm{CO}}_{2}$ is progressively consumed along the gas flow channel and GDE, while lighter gaseous products, particularly $H_{2}$, become increasingly important. This effect is already visible near the gas inlet (Figure 2b). At mild potentials, the region adjacent to the GDE remains comparatively slow‐moving, whereas at the most negative potentials it becomes more dynamic as gaseous products generated at the GDE surface enter the gas phase. Although the inlet volumetric flow rate of ${\mathrm{CO}}_{2}$ is fixed, product formation changes the local gas composition and density, and therefore modifies the velocity field close to the electrode. In addition, the corresponding decrease in gas density within the gas flow channel requires an increase in velocity to satisfy mass continuity. Gas‐side hydrodynamics are therefore not imposed solely by the inlet flow rate; they evolve with the local extent of electrochemical conversion. Through‐plane profiles across the full 80μm GDL thickness show comparatively small gradients in gas composition, with ${\mathrm{CO}}_{2}$ partial pressure decreasing slightly toward the catalyst‐layer boundary and $H_{2}$ partial pressure increasing in the opposite direction (Figures S5 and S6). By contrast, substantially larger changes in the absolute partial pressures develop over the 25 mm electrode length, particularly at the more negative cathode potentials. Under the conditions considered here, the dominant gas‐phase redistribution therefore occurs along the flow direction, demonstrating why resolving the longitudinal dimension is a must to capture the reactor‐scale behavior reported in this work.

The impact of these gas‐phase changes on reactor performance is shown in Figure 3. The model quantitatively reproduces the experimental increase in ${\mathrm{CO}}_{2}$‐to‐CO partial current density under apparent kinetic control and captures the subsequent departure from this Tafel‐like response as transport limitations become important (Figure 3a). This agreement supports the transport interpretation by linking the lower‐overpotential response to the transition to interfacial ${\mathrm{CO}}_{2}$ transport limitation as the overpotential increases. HER increases sharply at the most negative potentials, consistent with the predicted rise in $H_{2}$ molar fraction (Figure 3b). The CO‐to‐ethylene pathway also increases strongly over the relevant potential range (Figure 3c), showing that the model captures the experimentally observed transition toward $C_{2+}$‐dominated operation in the GDE used for validation [11]. The remaining CO‐derived products are interpreted in the Supporting Information (Figure S3), where they are used to identify the limits of the reduced availability‐based kinetic representation.

**FIGURE 3:**
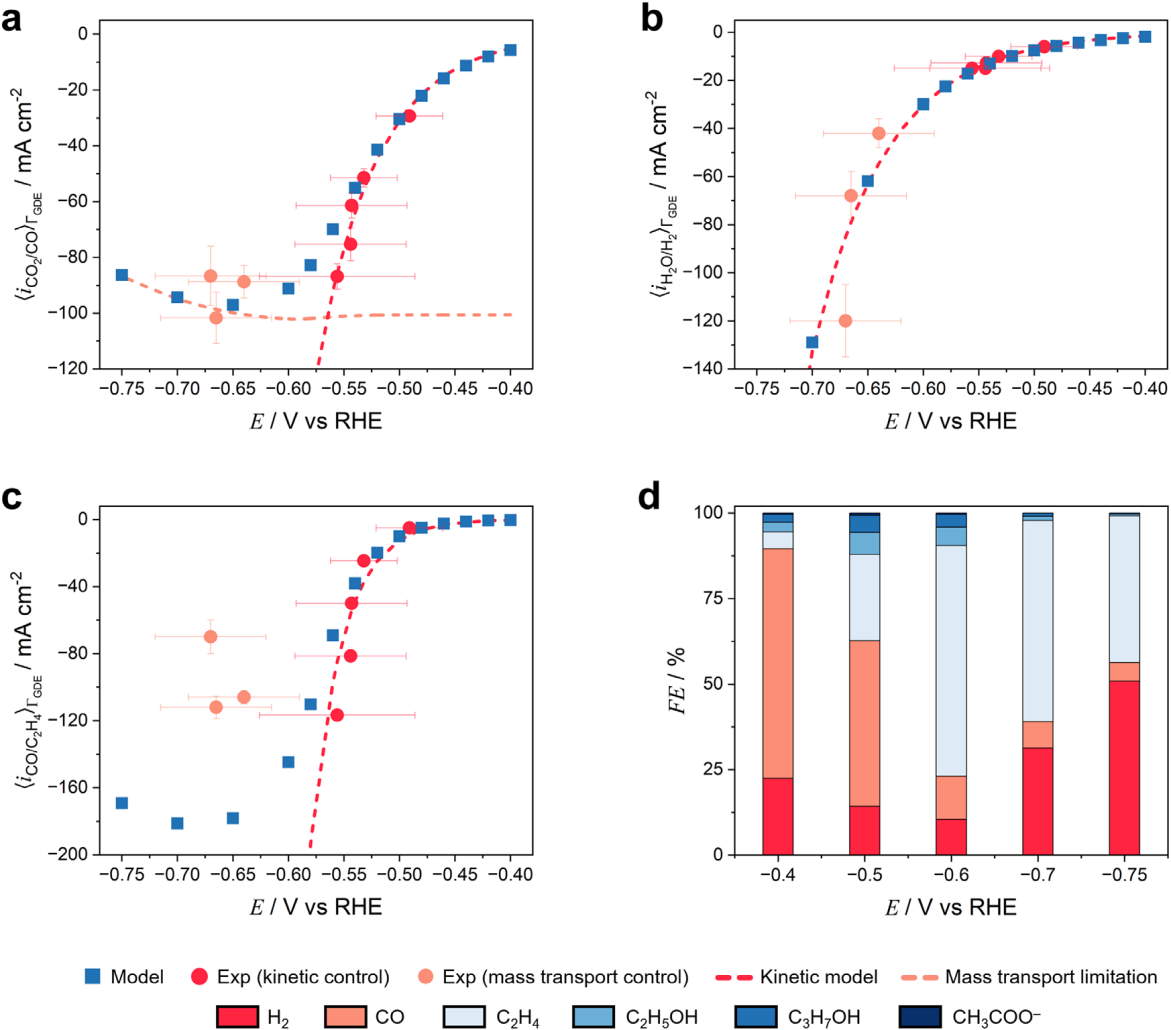
Experimental and simulated product‐current trends associated with the evolution of the gas phase. (a–c) Experimental and simulated partial current densities for the ${\mathrm{CO}}_{2}$‐to‐CO, HER and CO‐to‐ethylene ($C_{2}H_{4}$) pathways. (d) Faradaic efficiencies calculated from the simulated partial current densities. The model quantitatively reproduces the ${\mathrm{CO}}_{2}$‐to‐CO and HER responses across the relevant potential range and captures the main trend in ethylene formation, including the transition toward $C_{2+}$‐dominated operation.

The Faradaic efficiencies summarize this transition Figure 3d). At mild potentials, CO accounts for a large fraction of the carbon products. At intermediate potentials, ethylene becomes the dominant carbon product, reflecting the combined effect of sufficient overpotential, CO availability and favorable kinetics for the CO‐to‐ethylene pathway. At the most negative potentials, HER becomes increasingly important and the Faradaic efficiency toward carbon products decreases. This behavior already points to a scale‐up limitation: increasing current density by moving to more negative potentials also intensifies ${\mathrm{CO}}_{2}$ depletion, $H_{2}$ formation and gas‐composition changes, all of which feed back into local reactor performance.

### 2.2 Interfacial ${\mathrm{CO}}_{2}$ Utilization Controls the Onset of Transport Limitation

Once electrochemical conversion reshapes the gas phase, the next question is where this evolving gas‐phase environment begins to limit ${\mathrm{CO}}_{2}$ reduction at the catalyst surface. This matters because transport limitation in a GDE is spatially distributed: the electrode‐averaged current density can obscure whether ${\mathrm{CO}}_{2}$ remains available along the full electrode or is depleted locally near the gas–liquid interface. The transition to high current density therefore does not occur uniformly along the electrode. Figure 4 shows the local ${\mathrm{CO}}_{2}$‐to‐CO current density, the corresponding ${\mathrm{CO}}_{2}$ partial pressure at the gas side of the interface, the dissolved ${\mathrm{CO}}_{2}$ concentration at the catalyst surface, and the dimensionless ${\mathrm{CO}}_{2}$ utilization ratio.

**FIGURE 4:**
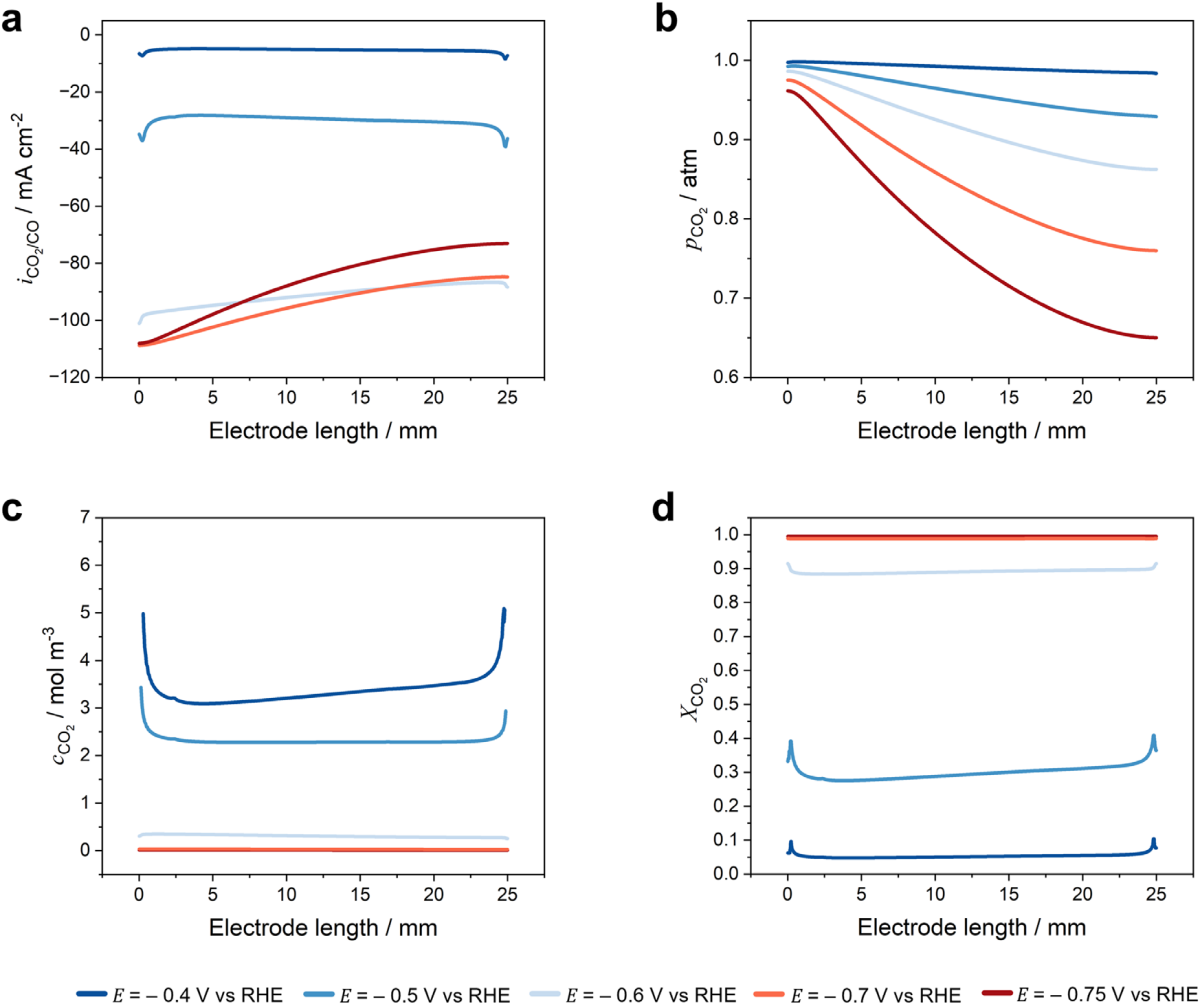
Spatial transition of the ${\mathrm{CO}}_{2}$‐to‐CO pathway from kinetic control to interfacial transport limitation. (a) Local partial current density for ${\mathrm{CO}}_{2}$‐to‐CO. (b) ${\mathrm{CO}}_{2}$ partial pressure at the gas side of the gas–liquid interface. (c) Dissolved ${\mathrm{CO}}_{2}$ concentration at the GDE surface. (d) Dimensionless ${\mathrm{CO}}_{2}$ utilization ratio, $X_{{\text{CO}}_{2}}$, defined as the ratio between the local ${\mathrm{CO}}_{2}$‐to‐CO current density and the maximum current density that would be supported if the interfacial ${\mathrm{CO}}_{2}$ flux were Faradaically converted to CO. Both gas and electrolyte inlets are located at electrode length =0 mm.

At mild cathodic potentials, the ${\mathrm{CO}}_{2}$‐to‐CO current density is relatively uniform along the GDE surface, indicating that the reaction remains under apparent kinetic control (Figure 4a). Only localized increases in current are observed near corner regions, which is consistent with edge effects and locally stagnant electrolyte flow [26]. Under these conditions, the amount of ${\mathrm{CO}}_{2}$ transported across the gas–liquid interface is larger than the amount electrochemically consumed, so dissolved ${\mathrm{CO}}_{2}$ remains available for homogeneous reactions in the electrolyte.

As the cathode potential becomes more negative, ${\mathrm{CO}}_{2}$ consumption increases and a longitudinal gradient develops in the gas phase (Figure 4b). The ${\mathrm{CO}}_{2}$ partial pressure decreases downstream, while the dissolved ${\mathrm{CO}}_{2}$ concentration at the catalyst surface approaches very low values. At the highest overpotentials, this depletion begins near the entrance of the gas flow channel, rather than only after substantial downstream conversion. This behavior is consistent with the inlet‐region velocity field (Figure 2b): gaseous products generated at the GDE surface, mainly $H_{2}$ from HER together with CO and ethylene, locally dilute ${\mathrm{CO}}_{2}$ and displace it from the gas–liquid interface (Figures S4, S5 and S6). As a result, the effective ${\mathrm{CO}}_{2}$ supply to the catalyst surface is altered even though the imposed inlet flow rate is fixed (Figure 4c). This indicates that the surface reaction consumes ${\mathrm{CO}}_{2}$ rapidly enough for interfacial gas‐to‐liquid transport to become the controlling process. The corresponding averaged current density no longer follows the Tafel‐like trend and instead approaches the limiting current estimated from the interfacial ${\mathrm{CO}}_{2}$ flux (Figure 3a).

The ${\mathrm{CO}}_{2}$ utilization ratio, $X_{{\text{CO}}_{2}}$, makes this transition clearer (Figure 4d). At mild potentials, $X_{{\text{CO}}_{2}}$ remains far below unity, indicating that only a small fraction of the ${\mathrm{CO}}_{2}$ crossing the gas–liquid interface is reduced to CO. A significant fraction of the dissolved ${\mathrm{CO}}_{2}$ can therefore participate in homogeneous bicarbonate/carbonate chemistry. At higher overpotentials, $X_{{\text{CO}}_{2}}$ approaches unity over most of the electrode length. In that regime, most of the ${\mathrm{CO}}_{2}$ transported across the interface is consumed electrochemically, and the homogeneous conversion of dissolved ${\mathrm{CO}}_{2}$ near the cathode becomes comparatively less important than at mild overpotentials.

This result also explains why the current response under mass transport control does not behave as a simple classical limiting‐current plateau (Figure 3a). In conventional electrochemical systems where the reactant is transported from the bulk electrolyte to the electrode surface, the limiting current is set by the corresponding bulk‐to‐surface mass‐transfer rate. Here, the gas phase is modified by the electrochemical reactions themselves. At high overpotentials, gaseous product formation changes the local gas composition, density and velocity field, while also displacing ${\mathrm{CO}}_{2}$ from the gas–liquid interface near the inlet. The limiting process is therefore not a fixed transport boundary condition, but a coupled consequence of ${\mathrm{CO}}_{2}$ consumption, gaseous product formation, gas composition and flow.

### 2.3 CO Availability Links ${\mathrm{CO}}_{2}$ Conversion to $C_{2+}$ Formation

The consequences of ${\mathrm{CO}}_{2}$ limitation are especially important for multicarbon products because CO is both generated from ${\mathrm{CO}}_{2}$ and consumed through downstream $C_{2+}$ pathways. The relevant quantity is therefore not simply how much CO is formed, but whether it remains locally available for further reduction along the electrode. The spatial availability of CO provides the link between ${\mathrm{CO}}_{2}$ transport and $C_{2+}$ selectivity. Figure 5 shows the local CO‐to‐ethylene current density together with the CO partial pressure, dissolved CO concentration, and CO usage ratio. The figure highlights that ethylene formation depends on a balance between CO generation from ${\mathrm{CO}}_{2}$ and CO consumption through $C_{2+}$ pathways.

**FIGURE 5:**
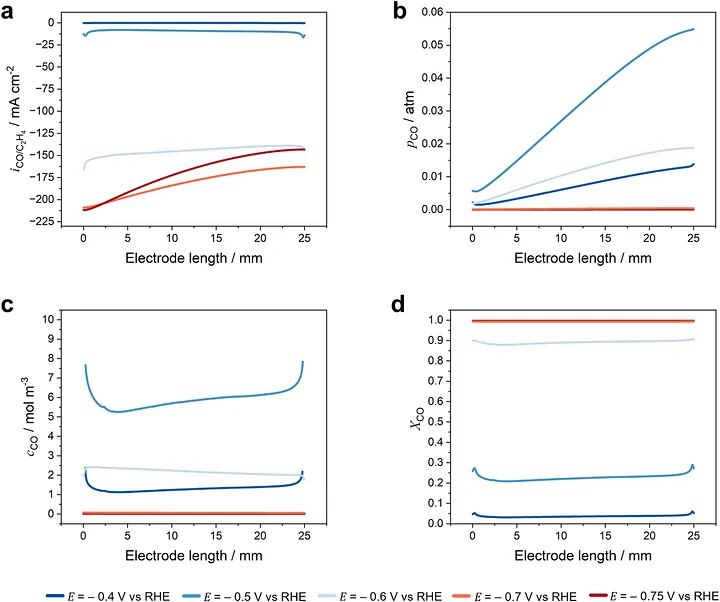
CO availability as the link between ${\mathrm{CO}}_{2}$ conversion and ethylene formation. (a) Local partial current density for the reduction of CO to ethylene ($C_{2}H_{4}$). (b,c) CO partial pressure at the gas–liquid interface and dissolved CO concentration at the GDE surface. (d) Dimensionless CO usage ratio, $X_{CO}$, which compares the rate of CO consumption toward $C_{2+}$ products with the rate of CO generation from ${\mathrm{CO}}_{2}$. Both gas and electrolyte inlets are located at electrode length =0 mm.

At intermediate cathode potentials, ${\mathrm{CO}}_{2}$‐to‐CO is active but CO consumption toward $C_{2+}$ products is not yet strong enough to fully deplete the intermediate (Figure 5a). As a result, CO accumulates in the gas phase and remains available at the catalyst surface (Figure 5b,c). This creates the operating window in which ethylene formation becomes favored. The dominance of ethylene over other CO‐derived products is not caused by CO availability alone; it also reflects the kinetic parameters of the competing CO‐reduction pathways. In other words, CO availability enables $C_{2+}$ formation, but the intrinsic pathway kinetics determine how the available CO is distributed among products.

At more negative potentials, the behavior changes. The ${\mathrm{CO}}_{2}$‐to‐CO pathway approaches transport limitation (Figure 3a), while the consumption of CO toward $C_{2+}$ products becomes stronger (Figures 3c and 5a). The CO partial pressure (Figure 5b) and dissolved CO concentration (Figure 5c) no longer increase monotonically, indicating that CO is consumed nearly as rapidly as it is generated. This is reflected by $X_{CO}$ (Figure 5d), which approaches unity in regions where the rate of CO consumption becomes comparable to the rate of CO production. Under these conditions, $C_{2+}$ formation becomes increasingly constrained by intermediate availability, as reflected by the local CO‐to‐$C_{2}H_{4}$ rate (Figure 5a) and the additional CO‐derived product distributions (Figure S8).

These results show that the highest ethylene rates are obtained in a coupled operating window. The system must generate enough CO to sustain C–C coupling, but not be driven so strongly that upstream ${\mathrm{CO}}_{2}$ depletion and rapid CO consumption make the intermediate limiting. At the same time, HER must remain sufficiently controlled so that $H_{2}$ formation does not dominate the gas composition or reduce the Faradaic efficiency toward carbon products (Figure S7). Beyond this window, increasing the overpotential mainly intensifies ${\mathrm{CO}}_{2}$ depletion, CO limitation, and HER, rather than improving carbon‐product formation.

The comparison with $C_{2}H_{5}\mathrm{OH}$ defines the boundary of this interpretation. Ethanol does not follow the same reduced CO‐availability response as ethylene, confirming that CO availability is necessary for $C_{2+}$ formation but not sufficient to determine product‐specific branching between $C_{2}H_{4}$, $C_{2}H_{5}\mathrm{OH}$ and other CO‐derived products (Figure S3). This behavior is consistent with the 1D MEA model of Weng et al. [17], where ethylene and ethanol were assigned distinct product‐specific kinetic parameters; in one kinetic parameterization, ethanol has a larger cathodic transfer coefficient and lower ${\mathrm{CO}}_{2}$ reaction order than ethylene, allowing ethanol to respond differently to overpotential and reactant depletion. It is also consistent with recent evidence that, despite sharing CO as an upstream intermediate, ethylene and ethanol formation can diverge through different downstream adsorbed intermediates and Cu active sites [5]. Beyond the availability of CO for C–C coupling, ethanol selectivity can also depend on the local hydrogenation environment, including proton/electron transfer, adsorbed hydrogen availability and sites that stabilize oxygenated $C_{2}$ intermediates. The comparison with ethanol therefore marks the boundary between reactor‐scale CO availability and product‐specific surface chemistry.

### 2.4 Local Electrolyte Chemistry Reflects the Competition Between Carbonation and ${\mathrm{OH}}^{-}$ Generation

The transition from kinetically favoured ${\mathrm{CO}}_{2}$‐to‐CO conversion to interfacial ${\mathrm{CO}}_{2}$ transport limitation also affects the electrolyte environment. Electrolyte effects are known to influence electrocatalytic kinetics, selectivity and stability through interfacial electrolyte–surface interactions [27]; Figure 6 shows how the local ${\mathrm{OH}}^{-}$ concentration, pH and carbon speciation evolve across the electrolyte flow channel as the cathode potential becomes more negative. Local variations in ${\mathrm{OH}}^{-}$ concentration do arise near the GDE surface and in stagnant corner regions (Figure 6a). Nevertheless, the electrolyte remains strongly alkaline throughout the electrolyte flow channel (Figure 6b) under the 1 M KOH conditions considered here. At mild overpotentials, a larger fraction of the ${\mathrm{CO}}_{2}$ transported across the gas–liquid interface remains unreacted electrochemically. This dissolved ${\mathrm{CO}}_{2}$ is rapidly converted into bicarbonate and, predominantly under strongly alkaline conditions, carbonate species, consuming ${\mathrm{OH}}^{-}$ in the process [28, 29]. In relative terms, this lowers the local pH near the GDE surface with respect to the strongly alkaline bulk, although the electrolyte remains alkaline throughout and the bulk pH barely changes.

**FIGURE 6:**
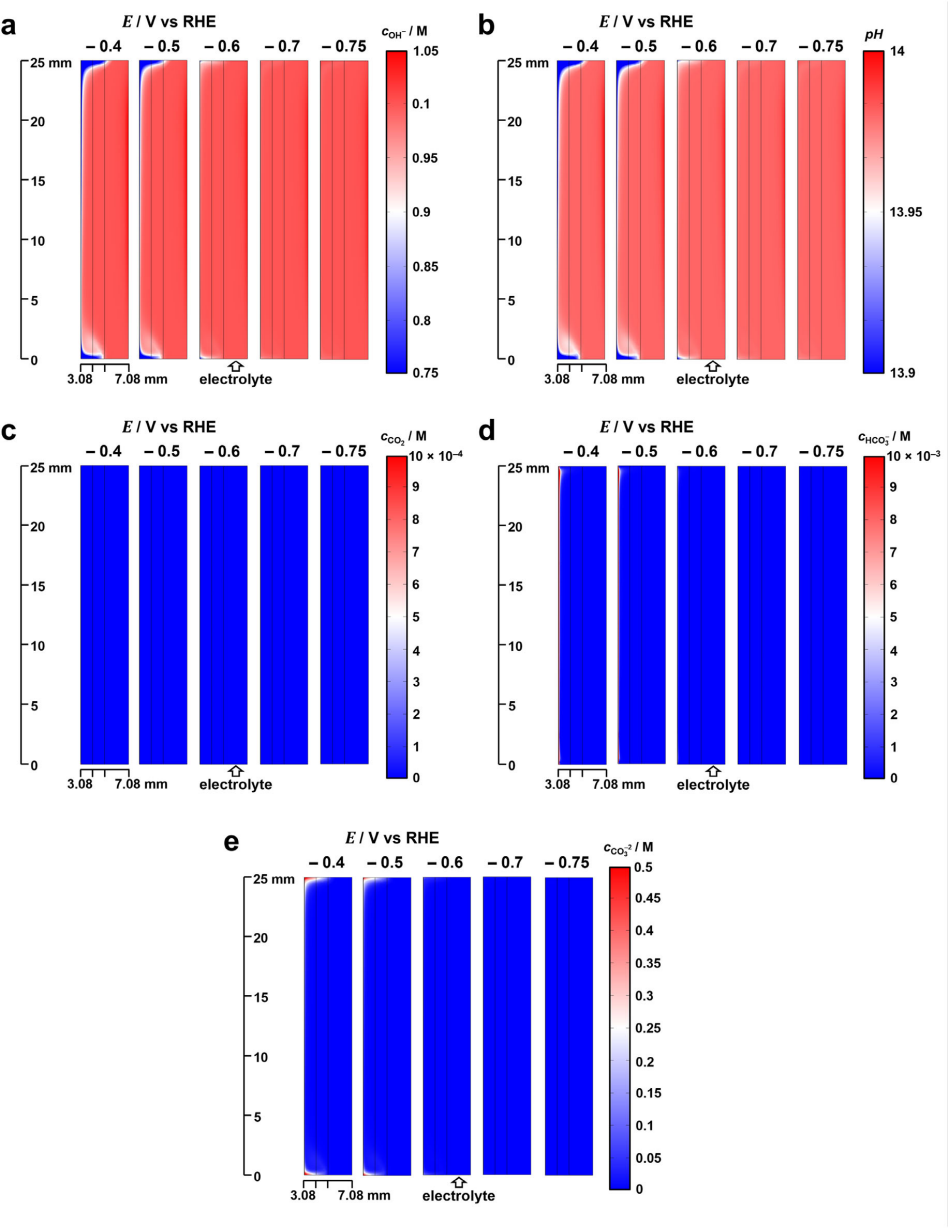
Local electrolyte chemistry across the catholyte channel at different applied cathode potentials. (a) ${\mathrm{OH}}^{-}$ concentration and (b) pH, showing that the electrolyte remains strongly alkaline while local variations develop close to the GDE/electrolyte interface and in stagnant regions. (c) Dissolved ${\mathrm{CO}}_{2}$ concentration, (d) ${\mathrm{HCO}}_{3}^{-}$ concentration and (e) ${\mathrm{CO}}_{3}^{2-}$ concentration, showing the redistribution of absorbed ${\mathrm{CO}}_{2}$ through homogeneous carbonate chemistry. The strongest changes occur close to the GDE/electrolyte interface, while the bulk electrolyte remains comparatively unaffected.

At higher overpotentials, the local pH gradient becomes less pronounced. This occurs because most of the ${\mathrm{CO}}_{2}$ transported across the interface is consumed electrochemically to form CO, leaving less dissolved ${\mathrm{CO}}_{2}$ available for homogeneous carbonation near the cathode. At the same time, HER and the other cathodic pathways generate ${\mathrm{OH}}^{-}$ at the electrode surface. The local electrolyte environment therefore shifts from one where absorbed ${\mathrm{CO}}_{2}$ can lower the local pH relative to the bulk, to one where electrochemical ${\mathrm{OH}}^{-}$ generation becomes increasingly dominant. A similar tendency toward local alkalization at greater overpotential was reported by Kas et al. in bicarbonate electrolyte [18]. This interpretation is also consistent with the operando Raman measurements of Lu et al. [30], who observed a near‐GDE pH gradient arising from ${\mathrm{CO}}_{2}$–${\mathrm{OH}}^{-}$ neutralization in 1 M KOH that became less pronounced as the ${\mathrm{CO}}_{2}$‐reduction current increased. They also showed that the extent of this gradient depends strongly on both the ${\mathrm{CO}}_{2}$ and electrolyte flow rates. The absolute pH values and spatial gradients are not directly comparable with those predicted here because of differences in reactor configuration, operating conditions, and, in the case of Kas et al., electrolyte composition. Nevertheless, the same underlying competition is observed: ${\mathrm{CO}}_{2}$ absorption and homogeneous reactions consume alkalinity, while cathodic reactions generate ${\mathrm{OH}}^{-}$.

The carbon speciation fields also show that free dissolved ${\mathrm{CO}}_{2}$ remains very low throughout the electrolyte flow channel (Figure 6c). The more informative changes are observed in ${\mathrm{HCO}}_{3}^{-}$ red (Figure 6d) and especially ${\mathrm{CO}}_{3}^{2-}$ (Figure 6e), which accumulate near the GDE surface and in stagnant regions of the electrolyte channel. At the full‐channel scale of Figure 6, much of the electrolyte therefore appears nearly uniform, but the corresponding through‐plane profiles (Figures S9, S10 and S11) show the local gradients much more clearly. At a given position along the electrode, these profiles could be described using a one‐dimensional through‐plane model: ${\mathrm{HCO}}_{3}^{-}$ remains concentrated mainly close to the GDE, whereas the pH and ${\mathrm{CO}}_{3}^{2-}$ gradients can extend farther into the electrolyte, particularly at mild overpotentials. However, both the size of these gradients and how far they extend into the electrolyte change with position along the electrode. This complements the gas‐phase behavior, where the through‐plane gradients across the GDL are comparatively weak, and the much larger changes develop along the flow direction (Figures S5 and S6). The 2D description is therefore important because it connects these local through‐plane electrolyte gradients with the longitudinal evolution of gas composition, ${\mathrm{CO}}_{2}$ delivery and electrochemical conversion along the reactor. Homogeneous carbonation consequently remains important for shaping the local catholyte environment, particularly at mild overpotentials, even though its relative importance near the cathode decreases as the system approaches interfacial ${\mathrm{CO}}_{2}$ transport limitation. These results suggest that the operating window identified from ${\mathrm{CO}}_{2}$ and CO utilisation may also be relevant to cathode‐side carbonate management, although precipitation and water‐balance effects are not explicitly resolved here.

It is worth noting that the acid–base and carbonate chemistry implemented in the model can be applied over a wider pH range; extending the present analysis to acidic or near‐neutral electrolytes would, however, require electrochemical kinetic parameters obtained under those conditions.

### 2.5 Utilisation Diagnostics Identify the Operating Window for $C_{2+}$ Formation

The spatial maps discussed above reveal where ${\mathrm{CO}}_{2}$ utilization and CO consumption become limiting, but scale‐up also requires compact diagnostics that summarize these local fields at the reactor level. This is important because operating decisions are often based on averaged current density or conversion, whereas the preceding results show that local ${\mathrm{CO}}_{2}$ depletion and CO consumption vary strongly along the electrode. Figure 7 addresses this point by averaging the local ${\mathrm{CO}}_{2}$ utilization and CO usage ratios along the GDE boundary. These averaged quantities condense the spatial profiles shown in Figures 4 and 5 into a compact description of the transition from excess ${\mathrm{CO}}_{2}$ supply to stronger interfacial utilization and increasing CO consumption. By plotting the corresponding ethylene partial current density in this space, the figure shows where ${\mathrm{CO}}_{2}$ supply is sufficient to sustain CO generation while CO consumption has not yet become strongly limiting.

**FIGURE 7:**
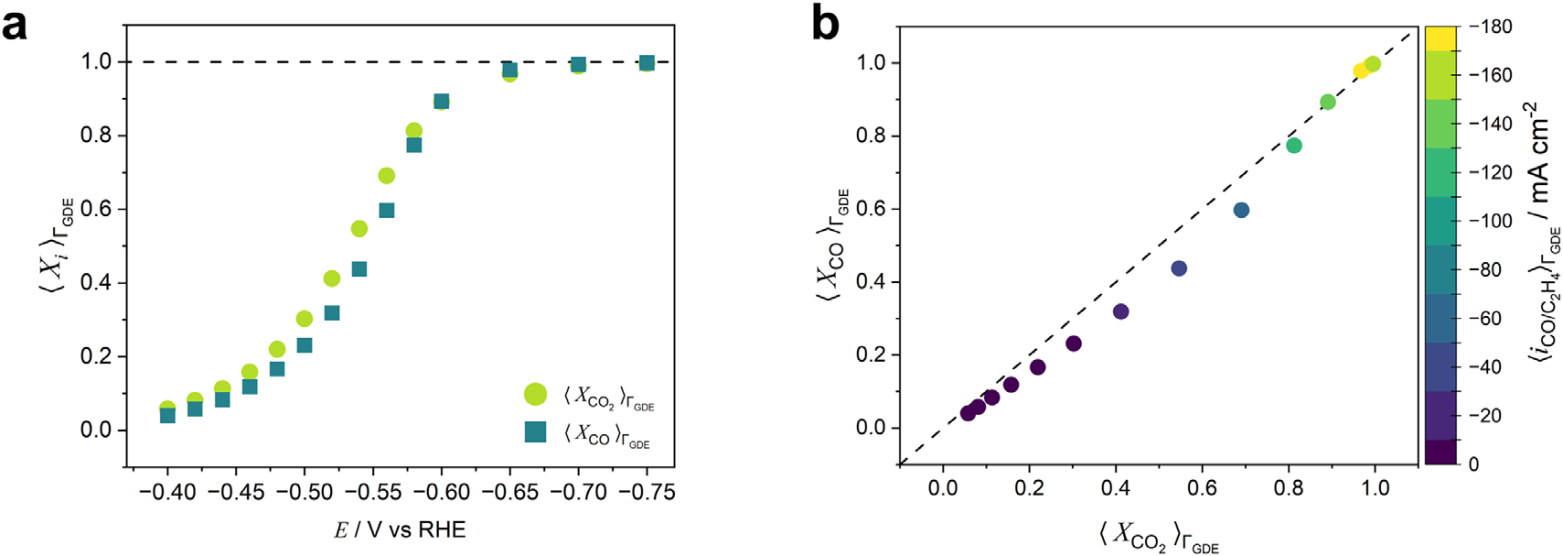
Utilization diagnostics for $C_{2+}$ formation in a flow reactor equipped with a GDE containing Cu‐based catalysts. (a) Electrode‐averaged ${\mathrm{CO}}_{2}$ utilization ratio, ${\left\langle X_{{\mathrm{CO}}_{2}}\right\rangle }_{\Gamma_{\mathrm{GDE}}}$, and CO usage ratio, ${\left\langle X_{\mathrm{CO}}\right\rangle }_{\Gamma_{\mathrm{GDE}}}$, as a function of applied cathode potential. The dashed horizontal line indicates the upper bound ${\left\langle X_{i}\right\rangle }_{\Gamma_{\mathrm{GDE}}}=1$. (b) Utilization map showing ${\left\langle X_{\mathrm{CO}}\right\rangle }_{\Gamma_{\mathrm{GDE}}}$ as a function of ${\left\langle X_{{\mathrm{CO}}_{2}}\right\rangle }_{\Gamma_{\mathrm{GDE}}}$, with color indicating the corresponding ethylene partial current density. The dashed diagonal marks ${\left\langle X_{\mathrm{CO}}\right\rangle }_{\Gamma_{\mathrm{GDE}}}={\left\langle X_{{\mathrm{CO}}_{2}}\right\rangle }_{\Gamma_{\mathrm{GDE}}}$; points below this line indicate that CO consumption has not yet become comparable to CO generation from ${\mathrm{CO}}_{2}$.

At low overpotential, both ${\left\langle X_{{\text{CO}}_{2}}\right\rangle }_{\Gamma_{\mathrm{GDE}}}$ and ${\left\langle X_{CO}\right\rangle }_{\Gamma_{\mathrm{GDE}}}$ remain well below unity (Figure 7a). The ${\mathrm{CO}}_{2}$‐to‐CO pathway is therefore close to apparent kinetic control, CO formation is limited, and a substantial fraction of the ${\mathrm{CO}}_{2}$ crossing the gas–liquid interface remains available for homogeneous bicarbonate/carbonate chemistry in the electrolyte.

At intermediate overpotential, ${\left\langle X_{{\text{CO}}_{2}}\right\rangle }_{\Gamma_{\mathrm{GDE}}}$ increases while ${\left\langle X_{CO}\right\rangle }_{\Gamma_{\mathrm{GDE}}}$ remains below the equality line (Figure 7b). This indicates that ${\mathrm{CO}}_{2}$ utilization is high enough to sustain CO generation, but that CO consumption has not yet caught up with CO production. This is the regime in which ethylene formation is favored in the system studied here.

At high overpotential, both ratios approach unity (Figure 7a) and the operating points move toward the upper‐right region of the map (Figure 7b). Under these conditions, most of the ${\mathrm{CO}}_{2}$ transported across the interface is consumed electrochemically, while CO consumption becomes increasingly comparable to CO generation. Further increases in overpotential are therefore expected to give diminishing returns for carbon‐product formation, particularly as HER also becomes more important and changes the gas composition and velocity field.

Together, these diagnostics show that the ethylene‐forming window is bounded by different limitations at low and high overpotential. At low overpotential, $C_{2+}$ formation remains limited by the modest driving force for downstream CO reduction together with the limited local build‐up of CO generated from ${\mathrm{CO}}_{2}$. As the overpotential increases, ${\mathrm{CO}}_{2}$ utilization increases while CO usage remains below CO generation, creating a favorable regime in which CO is generated and remains locally available as downstream $C_{2+}$ formation becomes more active. At high overpotential, interfacial ${\mathrm{CO}}_{2}$ utilization and CO usage both approach unity, so ${\mathrm{CO}}_{2}$ delivery and CO availability become increasingly constrained. The favorable regime therefore corresponds to an intermediate operating window in which ${\mathrm{CO}}_{2}$ delivery, CO generation, and controlled CO consumption remain spatially aligned, highlighting the broader value of the utilization‐based diagnostics defined in this work for interpreting and comparing gas‐fed ${\mathrm{CO}}_{2}$ electrolysers.

## 3 Discussion

These results can be interpreted within the classical framework of electrochemical reactor engineering, where reaction and transport are intrinsically coupled through reactant consumption and product generation [31]. The relevant scale‐up problem is therefore not only whether a catalyst, membrane or cell configuration can achieve a target current density or Faradaic efficiency under a given inlet composition, but whether the reactor can preserve the local reacting environment required for selectivity as current, electrode length and conversion increase. In a multi‐product GDE flow reactor, the gas phase is not simply a ${\mathrm{CO}}_{2}$ supply boundary; it becomes part of the electrochemical response, acting simultaneously as the feedstock reservoir, the product‐removal pathway and an evolving hydrodynamic domain. At high current density, electrochemical conversion changes the gas composition, density and velocity field, and this evolving gas phase feeds back into local ${\mathrm{CO}}_{2}$ delivery, CO availability and product selectivity. Gas‐phase hydrodynamics therefore become dynamically coupled to the electrochemical response. The key implication is that, in scaled GDE operation, the local reaction environment is generated in part by the reactor itself, rather than imposed only by inlet flow rate, applied potential or bulk electrolyte composition. This reactor‐scale interpretation is summarized schematically in Figure 8, which shows how the low‐, intermediate‐ and high‐overpotential regimes differ in ${\mathrm{CO}}_{2}$ transport, CO availability, gaseous product formation and gas‐phase hydrodynamic feedback.

**FIGURE 8:**
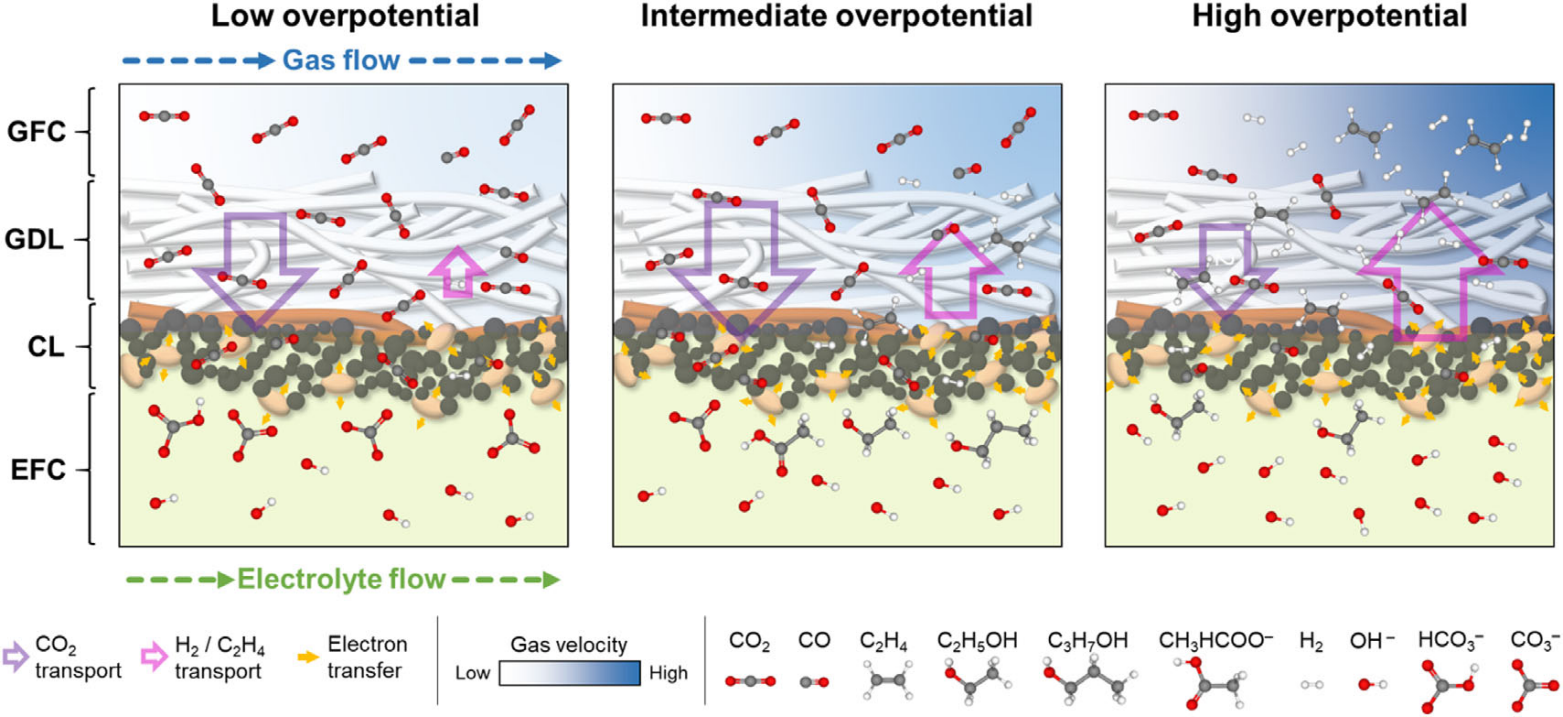
Reactor‐scale interpretation of the operating regimes identified by the utilization diagnostics. At low overpotential, interfacial ${\mathrm{CO}}_{2}$ supply exceeds electrochemical consumption, and CO formation is limited. At intermediate overpotential, ${\mathrm{CO}}_{2}$ utilization increases while CO usage remains lower, favoring $C_{2+}$ formation. At high overpotential, ${\mathrm{CO}}_{2}$ utilization approaches transport limitation, CO availability becomes increasingly constrained and product‐induced gas‐phase composition changes feed back into reactant delivery. The schematic highlights that the relevant scale‐up objective is to preserve the spatial overlap between ${\mathrm{CO}}_{2}$ delivery, CO generation, controlled CO consumption and limited dilution of the reacting gas phase.

This coupling is not captured by global performance metrics such as electrode‐averaged current density, single‐pass conversion or overall Faradaic efficiency. A reactor can reach high current density or high average ${\mathrm{CO}}_{2}$ conversion while redistributing reaction rates and selectivity along the electrode. At mild overpotentials, the ${\mathrm{CO}}_{2}$‐to‐CO pathway remains close to apparent kinetic control and a substantial fraction of transferred ${\mathrm{CO}}_{2}$ can still participate in homogeneous electrolyte chemistry. At high overpotentials, most of the ${\mathrm{CO}}_{2}$ crossing the interface is consumed electrochemically, the system approaches interfacial transport limitation, and gaseous product formation reshapes the gas phase. The transition away from kinetic control is therefore not equivalent to a classical limiting‐current plateau set by a fixed hydrodynamic boundary condition. It emerges from the coupled evolution of ${\mathrm{CO}}_{2}$ consumption, product formation, gas composition, and flow.

The utilization diagnostics introduced here provide a compact way to identify this redistribution. The relevant limitation is not only how much ${\mathrm{CO}}_{2}$ is consumed on average, but where that consumption occurs and how it changes the local reaction environment. If ${\mathrm{CO}}_{2}$ consumption is concentrated upstream, downstream regions operate under lower reactant availability, while CO availability, gas composition, and local selectivity also change along the reactor. The ${\mathrm{CO}}_{2}$ utilization ratio identifies where interfacial ${\mathrm{CO}}_{2}$ supply is being consumed electrochemically, while the CO usage ratio identifies where CO consumption approaches CO generation. These ratios also provide dimensionless measures of the local reactor response without relying on a fixed reference current. This is important here because the interfacial ${\mathrm{CO}}_{2}$ transport limit changes as the reacting gas phase evolves, while CO is generated locally rather than supplied at a fixed bulk concentration. Together, these quantities define whether the reactor is operating under excess ${\mathrm{CO}}_{2}$ supply, within a favorable ${\mathrm{CO}}_{2}$‐delivery/CO‐availability window, or close to a regime in which interfacial ${\mathrm{CO}}_{2}$ utilization and CO usage constrain further $C_{2+}$ formation. Although calculated locally in the present model, the same utilization‐based logic can guide experimental interpretation through coupled reactant‐consumption, product‐formation and outlet‐composition balances.

This interpretation is consistent with the trade‐off identified by Kas et al. [18], who showed that, at high single‐pass conversions, both ${\mathrm{CO}}_{2}$ and buffer species can be progressively depleted along the channel while homogeneous electrolyte reactions further limit the maximum achievable conversion. The present results extend this process‐level argument to a multi‐product GDE cathode operating in strongly alkaline medium: strong local ${\mathrm{CO}}_{2}$ consumption does not only affect outlet conversion, but also changes where CO is generated, where it remains available for $C_{2+}$ formation, and how gas‐phase hydrodynamics evolve along the electrode. The need to interpret ${\mathrm{CO}}_{2}$ electroreduction using coupled measures of reactant utilization, product formation and outlet composition also aligns with recent reports arguing that practically relevant product streams cannot be assessed from conversion alone, and that product concentration and reactor utilization are more meaningful scale‐up targets [32].

The novelty of this work lies not simply in including these effects within the same numerical framework, but in showing that their coupling changes how reactor performance is interpreted. Earlier continuum models addressed the central questions of their respective systems by resolving how ${\mathrm{CO}}_{2}$ supply, electrolyte chemistry, membrane crossover, CO management or catalyst‐layer transport shape local current density and product formation [16, 17, 18, 21, 33]. Here, multi‐product operation makes the transport environment itself part of the response: ${\mathrm{CO}}_{2}$‐to‐CO formation, downstream CO consumption and gaseous product evolution reshape the gas composition, density and velocity along the electrode. The resulting hydrodynamic feedback changes local reactant delivery and defines the operating window for multicarbon formation.

The same interpretation may also inform cathode‐side carbonate management. In alkaline and near‐neutral ${\mathrm{CO}}_{2}$ electrolysers, homogeneous reaction of absorbed ${\mathrm{CO}}_{2}$ with ${\mathrm{OH}}^{-}$ forms bicarbonate and carbonate species, causing carbon and energy losses and, under suitable water‐balance and cation‐transport conditions, contributing to salt accumulation and GDE degradation [34, 35]. Operation within the intermediate ${\mathrm{CO}}_{2}$‐delivery/CO‐availability window identified here could reduce unnecessary ${\mathrm{CO}}_{2}$ absorption into the catholyte by favoring electrochemical conversion at the GDE surface, while avoiding the severe ${\mathrm{CO}}_{2}$ starvation that promotes local alkalinization, HER and salt precipitation [36]. Quantifying this secondary implication would require explicit treatment of salt precipitation and water balance.

The role of CO illustrates why this coupling is important for selectivity. Ethylene formation is favored only when ${\mathrm{CO}}_{2}$ transport is sufficient to sustain CO generation while CO consumption remains below CO production. At more negative potentials, stronger ${\mathrm{CO}}_{2}$ utilization, faster CO consumption and increasing HER narrow this window. CO availability is therefore a necessary reactor‐scale condition for $C_{2+}$ formation, but not a complete descriptor of product branching among ethylene, ethanol and other CO‐derived pathways. The comparison with ethanol therefore separates reactor‐scale intermediate availability from catalyst‐level control over pathway‐specific branching.

The cathode‐side framework and reduced kinetic representation are matched to the reactor‐scale question addressed here. The main conclusions follow from conservation constraints rather than from a complete microkinetic description of every CO‐derived product or a full‐device description of every MEA process: ${\mathrm{CO}}_{2}$ must be transported to the interface before it can form CO, CO must remain locally available before it can support $C_{2+}$ formation, and gaseous products alter gas composition, density and velocity through mass continuity. More detailed descriptions of membrane transport, carbonate/bicarbonate and product crossover, water management or microscopic catalyst‐layer structure may be required to address other device‐specific questions or operating regimes. Gas–liquid transfer and dissolved $H_{2}$ are included in the model, with the corresponding saturation behavior discussed further in the Supporting Information. However, the electrolyte is treated as a single‐phase liquid; subsequent nucleation, growth and transport of discrete gas bubbles are not explicitly resolved. At high HER rates, such bubbles could further affect liquid‐phase transport, local wetting and the electrochemically active area. All of these phenomena would refine the quantitative operating point, which will depend on device architecture, flow field, catalyst‐layer structure and parameterization. However, they would not remove the conservation‐driven feedback identified here once interfacial reactant transport becomes controlling; in fact, any gas‐fed electrochemical reactor in which gaseous reactants are consumed and gaseous products are generated must develop changes in gas composition, density and velocity through mass conservation. In small cells, short residence times or excess ${\mathrm{CO}}_{2}$ supply may partly hide this effect; as electrode length, conversion and gaseous product formation increase, the same feedback necessarily becomes more important. Consistent with this scale‐up interpretation, recent stack‐level analysis has identified pressure, concentration and flow‐distribution gradients as central challenges for CO and ${\mathrm{CO}}_{2}$ electrolysis [13]. The present results provide a cathode‐side, cell‐level mechanism by which such gradients can arise from the electrochemical conversion itself, linking local interfacial transport limitation to stack‐relevant mass‐transport non‐uniformity.

## 4 Conclusions

Overall, this work establishes multi‐product ${\mathrm{CO}}_{2}$ electroreduction in GDE flow reactors as a coupled hydrodynamic–electrochemical reactor‐engineering problem in which the reactor generates part of the local reaction environment that controls its own performance. Scaling to $C_{2+}$ products therefore requires maintaining the spatial overlap between ${\mathrm{CO}}_{2}$ delivery, CO generation, controlled CO consumption and limited product‐driven dilution of the reacting gas phase, rather than pursuing current density or conversion without regard to where these processes occur. This shifts the scale‐up objective from maximizing global performance metrics alone to designing gas‐fed electrolysers that preserve favorable reaction environments over extended electrode areas. The modeling framework provides a route to identify when this overlap is lost and to guide gas‐channel, flow‐field architecture and operating‐window design so that mass‐transport uniformity is preserved as electrode area and stack complexity increase.

## 5 Methods

### 5.1 Model Domain and Validation System

The model represents the cathodic compartment of a modified flow electrochemical reactor equipped with a commercial anion exchange membrane and a GDE previously reported experimentally [11]. The simulated GDE consisted of a PTFE‐based gas diffusion layer, a sputtered Cu film and a porous catalyst layer containing mixed copper oxide particles dispersed in carbon black.

The 2D geometry represents a longitudinal cross‐section including the gas flow channel, GDE and electrolyte flow channel. The corresponding cell orientation and model section are shown in Figure 1. The model therefore resolves variations along the electrode length (y) and in the through‐plane direction (x) across the gas channel, GDE and electrolyte channel, while assuming uniformity across the electrode width (z). The GDE was represented as an 80μm‐thick porous gas diffusion layer coupled to a reactive catalyst‐layer boundary. The 3.6μm‐thick catalyst layer was represented by this boundary rather than explicitly resolved as a separate domain, so variations in reaction rate through its thickness are not captured. This treatment avoids explicitly meshing the catalyst layer while retaining the longitudinal and through‐plane transport gradients relevant to the reactor‐scale phenomena discussed in the main text. The resulting through‐plane gas‐phase profiles show that composition gradients across the 80μm GDL are comparatively small under the conditions considered here, whereas substantially larger changes develop along the electrode length. This choice prioritizes the reactor‐scale gradients that are central to this study, particularly the evolution of gas composition, density, velocity, ${\mathrm{CO}}_{2}$ partial pressure, CO availability and reaction rate along the electrode length.

Model parameterization and validation were based on the same experimental platform used to define the simulated reactor, for which geometry, flow conditions, electrolyte composition, GDE structure and product‐current data were available. This provides a controlled basis for quantitative interpretation of the ${\mathrm{CO}}_{2}$‐to‐CO transition and HER response, trend‐level assessment of CO‐to‐$C_{2}H_{4}$, and qualitative/order‐of‐magnitude interpretation of additional CO‐derived products.

### 5.2 Gas‐Phase Transport

Gas flow in the gas flow channel was described as laminar, ideal and compressible under isothermal conditions. The gas continuity and momentum balances were

(1) $$\frac{\partial \rho }{\partial t}+\nabla \cdot \left(\rho u\right)=0,$$

and

(2) $$\rho \frac{Du}{Dt}=-\nabla p+\nabla \cdot \tau ,$$

where ρ is the gas density, t is time, u is the free‐flow gas velocity in the gas flow channel, D/Dt is the material derivative, p is pressure and τ is the viscous stress tensor.

Flow through the porous GDE was described using Brinkman equations, which account for viscous shear and porous‐media resistance. In the porous GDE, u denotes the superficial, or Darcy, velocity averaged over the total cross‐sectional area of the porous medium; the corresponding interstitial pore velocity is $u/\varepsilon_{\mathrm{GDE}}$. The porous‐domain continuity and momentum balances were

(3) $$\frac{\partial }{\partial t}\left(\varepsilon_{\mathrm{GDE}}\rho \right)+\nabla \cdot \left(\rho u\right)=0,$$

and

(4) $$\frac{\rho }{\varepsilon_{\mathrm{GDE}}}\left[\frac{\partial u}{\partial t}+\left(u\cdot \nabla \right)\left(\frac{u}{\varepsilon_{\mathrm{GDE}}}\right)\right]=-\nabla p+\nabla \cdot \left(\frac{1}{\varepsilon_{\mathrm{GDE}}}\tau \right)-\frac{\mu }{\kappa_{\mathrm{GDE}}}u.$$

Here $\varepsilon_{\mathrm{GDE}}$ is the GDE porosity, μ is the gas viscosity and $\kappa_{\mathrm{GDE}}$ is the GDE permeability. Gas consumption, generation and gas–liquid exchange were imposed through interfacial species fluxes at the catalyst‐layer boundary.

The conservation equation for gas species i was written in terms of mass fraction. In the gas flow channel,

(5) $$\frac{\partial (\rho \omega_{i})}{\partial t}+\nabla \cdot \left(\rho \omega_{i}u\right)=-\nabla \cdot j_{i},$$

whereas in the porous GDE,

(6) $$\frac{\partial (\varepsilon_{\mathrm{GDE}}\rho \omega_{i})}{\partial t}+\nabla \cdot \left(\rho \omega_{i}u\right)=-\nabla \cdot j_{i}.$$

Here $\omega_{i}$ is the gas‐phase mass fraction of species i and $j_{i}$ is its diffusive mass flux. No volumetric gas‐phase reaction source was applied in the gas flow channel or porous GDE. Species exchange with the electrolyte and gaseous product generation were imposed through boundary fluxes at the catalyst‐layer interface.

Multicomponent diffusion was described using Maxwell–Stefan theory. In the porous GDE, effective binary diffusivities and Knudsen diffusivities were corrected using the Bruggeman correlation. Gas density and viscosity were updated iteratively based on the local composition.

### 5.3 Liquid‐Phase Transport and Homogeneous Chemistry

The electrolyte was treated as an incompressible single‐phase liquid with laminar flow:

(7) ∇·u=0,

where u is the electrolyte velocity. Dissolved species were transported by diffusion, migration and convection using the Nernst–Planck flux:

(8) $$N_{i}=-D_{i}\nabla c_{i}-z_{i}u_{m,i}Fc_{i}\nabla \phi_{L}+c_{i}u,$$

where $N_{i}$ is the total molar flux of dissolved species i, $D_{i}$ is its diffusivity, $c_{i}$ is its concentration, $z_{i}$ is its charge number, $u_{m,i}$ is its mobility, F is Faraday's constant and $\phi_{L}$ is the electrolyte potential. Electroneutrality was imposed in the electrolyte:

(9) $$\sum_{i}z_{i}c_{i}=0,$$

where the summation is over all charged dissolved species.

Homogeneous reactions among dissolved ${\mathrm{CO}}_{2}$, ${\mathrm{HCO}}_{3}^{-}$, ${\mathrm{CO}}_{3}^{2-}$, $H^{+}$ and ${\mathrm{OH}}^{-}$ were included to capture carbonate–water chemistry and local pH changes. The net rate of each reversible homogeneous reaction was defined using the law of mass action:

(10) $$R_{j}=k_{j,f}\prod_{i\in \mathrm{reactants}}c_{i}^{|\nu_{j,i}|}-k_{j,r}\prod_{i\in \mathrm{products}}c_{i}^{|\nu_{j,i}|}.$$

where $R_{j}$ is the net rate of homogeneous reaction j, $k_{j,f}$ and $k_{j,r}$ are the forward and reverse rate constants, respectively, and $\nu_{j,i}$ is the stoichiometric coefficient of species i in reaction j. The formal carbonate–water reaction set, stoichiometric dependencies and finite‐rate implementation are described in the Supporting Information (Table S2). These reactions account for the rapid redistribution of absorbed ${\mathrm{CO}}_{2}$ into ${\mathrm{HCO}}_{3}^{-}$ and ${\mathrm{CO}}_{3}^{2-}$ under alkaline conditions, and therefore provide the basis for interpreting the local ${\mathrm{OH}}^{-}$, pH and carbon‐speciation fields discussed in the Results section.

### 5.4 Gas–Liquid Mass Transfer

Transport of ${\mathrm{CO}}_{2}$, CO, $C_{2}H_{4}$ and $H_{2}$ across the gas–liquid interface was described using a thin‐film approximation:

(11) $$J_{i,G-L}=-D_{i,G-L}\frac{c_{i,G-L}^{t}-c_{i,G-L}^{*}}{\delta_{G-L}},$$

where $J_{i,G-L}$ is the interfacial molar flux of species i, $D_{i,G-L}$ is the effective diffusivity across the interfacial region, $c_{i,G-L}^{t}$ is the electrolyte‐side concentration, $c_{i,G-L}^{*}$ is the equilibrium concentration at the gas–liquid interface and $\delta_{G-L}$ is the effective transfer length. The equilibrium concentration was calculated from Henry's law, $c_{i,G-L}^{*}=p_{i}H_{i}$, where $p_{i}$ is the gas‐side partial pressure and $H_{i}$ is Henry's constant for species i. The effective transfer length was taken as $\delta_{G-L}=3.6\;\mu m$, corresponding to the catalyst‐layer thickness. This value should not be interpreted as a microscopic liquid‐film thickness. Rather, it provides an effective transfer length of the same order as the catalyst‐layer thickness, representing gas–liquid exchange through a wet and tortuous interfacial region in which active sites may be distributed at different distances from the gas–liquid interface.

### 5.5 Electrochemical Reaction Network and Kinetics

The electrochemical reaction network consisted of HER, ${\mathrm{CO}}_{2}$‐to‐CO, and the subsequent reduction of CO to ${\mathrm{CH}}_{3}{\mathrm{COO}}^{-}$, $C_{2}H_{4}$, $C_{2}H_{5}\mathrm{OH}$ and $C_{3}H_{7}\mathrm{OH}$. The reactions were represented as reduced lumped pathways:

- ${\mathrm{CO}}_{2}$ + $H_{2}O$ + 2$e^{-}$ → CO + 2${\mathrm{OH}}^{-}$
- 2CO + 3$H_{2}O$ + 4$e^{-}$ → ${\mathrm{CH}}_{3}\mathrm{COO}$‐ + 3${\mathrm{OH}}^{-}$
- 2CO + 6$H_{2}O$ + 8$e^{-}$ → $C_{2}H_{4}$ + 8${\mathrm{OH}}^{-}$
- 2CO + 7$H_{2}O$ + 8$e^{-}$ → $C_{2}H_{5}\mathrm{OH}$ + 8${\mathrm{OH}}^{-}$
- 3CO + 10$H_{2}O$ + 12$e^{-}$ → $C_{3}H_{7}\mathrm{OH}$ + 12${\mathrm{OH}}^{-}$
- 2$H_{2}O$ + 2$e^{-}$ → $H_{2}$ + 2${\mathrm{OH}}^{-}$

This reaction network is used as a reduced kinetic representation of the reactor‐scale coupling between ${\mathrm{CO}}_{2}$ transport, CO generation, CO consumption and product selectivity, rather than as a microkinetic description of all adsorbed intermediates or elementary steps on Cu‐based surfaces.

The cathodic current density of electrochemical reaction j was calculated from a simplified Butler–Volmer/Tafel expression:

(12) $$i_{j}=-i_{j,\mathrm{ref}}^{e}g_{j,c}\exp\left(-\frac{\alpha_{j,c}F}{RT}\eta_{j}\right),$$

where $i_{j}$ is the partial current density of electrochemical pathway j, $i_{j,\mathrm{ref}}^{e}$ is the exchange current density under reference conditions, $g_{j,c}$ is the cathodic concentration‐dependence term, $\alpha_{j,c}$ is the cathodic transfer coefficient, $\eta_{j}$ is the overpotential, and F, R and T are Faraday's constant, the ideal gas constant and temperature, respectively. The concentration‐dependence term was defined as

(13) $$g_{j,c}=\prod_{i\in O}\frac{c_{i}}{c_{i}+c_{i}^{*}},$$

where $c_{i}$ is the local concentration of reactant i, $c_{i}^{*}$ is a threshold concentration that determines the scale at which local reactant limitation becomes significant, and O denotes the oxidized‐side reactants in the corresponding cathodic pathway. For both ${\mathrm{CO}}_{2}$ and CO, $c_{i}^{*}=0.33$ mM was used.

The term $g_{j,c}$ was interpreted in the sense of the practical Butler–Volmer formulation, in which concentration‐dependent factors are defined relative to chosen reference conditions and recover unity at those conditions [37]. For the reduced pathways used here, this reference condition was taken to correspond to local non‐limiting reactant availability, rather than to a universal standard concentration. The bounded form in Equation (13) therefore gives $g_{j,c}\approx 1$ under apparent kinetic control and decreases smoothly only when the local reactant concentration becomes comparable to $c_{i}^{*}$. This captures local reactant availability without forcing the fitted kinetic prefactor to absorb an arbitrary concentration‐reference penalty, and without imposing full stoichiometric reaction orders on lumped product‐forming pathways.

The overpotential was defined as

(14) $$\eta_{j}=E-E_{j,\mathrm{ref}}^{e},$$

where E is the local electrode potential and $E_{j,\mathrm{ref}}^{e}$ is the equilibrium potential under the chosen reference conditions for pathway j. Equilibrium potentials were referenced to the alkaline HER potential using Nernst‐type expressions. Kinetic parameters were obtained by fitting Equation 12 to experimental partial current densities measured under apparent kinetic control for the same GDE platform [11]. The equilibrium potentials and fitted kinetic parameters are reported in Tables S3 and S4, respectively.

### 5.6 Diagnostic utilization Ratios

Two diagnostic ratios were defined to interpret transport limitation and intermediate availability. The maximum current density supported by interfacial ${\mathrm{CO}}_{2}$ transfer for the ${\mathrm{CO}}_{2}$‐to‐CO pathway was estimated from

(15) $$i_{{\mathrm{CO}}_{2}/\mathrm{CO},\lim}=-\frac{n_{{\mathrm{CO}}_{2}/\mathrm{CO}}FJ_{{\mathrm{CO}}_{2},G-L}}{|\nu_{{\mathrm{CO}}_{2},{\mathrm{CO}}_{2}/\mathrm{CO}}|}.$$

The corresponding ${\mathrm{CO}}_{2}$ utilization ratio was

(16) $$X_{{\mathrm{CO}}_{2}}=\frac{|i_{{\mathrm{CO}}_{2}/\mathrm{CO}}|}{|i_{{\mathrm{CO}}_{2}/\mathrm{CO},\lim}|}\leq 1.$$

The CO usage ratio was defined as

(17) $$X_{\mathrm{CO}}=\frac{\sum_{k}\frac{|\nu_{\mathrm{CO}/k}\left|\;\right|i_{\mathrm{CO}/k}|}{n_{\mathrm{CO}/k}F}}{\frac{|\nu_{\mathrm{CO},{\mathrm{CO}}_{2}/\mathrm{CO}}\left|\;\right|i_{{\mathrm{CO}}_{2}/\mathrm{CO}}|}{n_{{\mathrm{CO}}_{2}/\mathrm{CO}}F}}\leq 1.$$

Here, $i_{{\mathrm{CO}}_{2}/\mathrm{CO},\lim}$ is the local limiting current density associated with interfacial ${\mathrm{CO}}_{2}$ transfer, $n_{{\mathrm{CO}}_{2}/\mathrm{CO}}$ and $n_{\mathrm{CO}/k}$ are the numbers of electrons transferred in the corresponding pathways, $J_{{\mathrm{CO}}_{2},G-L}$ is the interfacial ${\mathrm{CO}}_{2}$ molar flux, $\nu_{i,j}$ is the stoichiometric coefficient of species i in pathway j, $i_{j}$ is the partial current density of pathway j, and k indexes the CO‐derived product pathways included in the reduced network. $X_{{\text{CO}}_{2}}$ was used to quantify the degree to which ${\mathrm{CO}}_{2}$ transferred across the gas–liquid interface is consumed electrochemically, whereas $X_{CO}$ was used to compare the rate of CO consumption toward $C_{2+}$ products with the rate of CO generation from ${\mathrm{CO}}_{2}$. These quantities were evaluated during post‐processing and were not imposed as hard constraints in the numerical solution.

### 5.7 Numerical Implementation

The model was implemented in COMSOL Multiphysics 6.2. The gas‐phase porous‐media transport was solved using Brinkman flow, with the porous‐domain velocity interpreted as the superficial gas velocity, and transport of concentrated species in porous media. Electrolyte transport and charge conservation were solved using the Tertiary Current Distribution, Nernst–Planck formulation. The simulations were solved through a staged procedure in which open‐circuit flow and transport fields were first established and electrochemical reactions were then enabled at the applied cathode potential. Transient simulations were run until quasi‐steady partial current densities were obtained. Gas density and viscosity were updated iteratively based on local composition, and the final quasi‐steady solution was used for analysis. Further details of the model parameters (Table S1), boundary conditions (Table S5 and Figure S1), mesh (Figure S2) and supplementary diagnostics are provided in the Supporting Information.

## Author Contributions

A.A.A. and I.T. conceived the modelling study and developed the modelling methodology. A.A.A. implemented the model, performed the simulations, analysed the results and prepared the figures under the supervision of I.T. and T.K. T.K. contributed to formalising the governing equations, the physical interpretation of the transport framework and the rigorous mathematical and physical assessment of the modelling approach. M.S.T.‐O. initiated simulation work in the group and provided early COMSOL training, modelling guidance and technical support to A.A.A. B.G.M. contributed to translating the modelling framework into Python and to discussions on numerical implementation. S.M.‐L. contributed to the interpretation of the Cu‐based electrocatalysis, product‐selectivity trends and experimental validation context. M.E.‐E. contributed to the interpretation of the Cu‐based electrocatalysis, product‐selectivity trends, mechanistic interpretation of the reduced kinetic framework and refinement of the manuscript framing. I.T. led the reactor‐scale interpretation, conceptual framing and overall manuscript development. I.T. wrote the manuscript with contributions from A.A.A. and critical input from all authors. All authors discussed the results, reviewed and edited the manuscript, and approved the final version.

## Funding

A.A.A., T.K. and I.T. are grateful to King Abdulaziz City for Science and Technology (KACST) in Saudi Arabia for sponsoring A.A.A.'s PhD research at the University of Edinburgh. M.S.T.‐O. and I.T. thank Consejo Nacional de Humanidades, Ciencias y Tecnologías (CONAHCYT) in Mexico for supporting M.S.T.‐O. through an overseas PhD scholarship. B.G.M. acknowledges financial support from the Beit Trust and The University of Edinburgh through a joint scholarship to pursue postgraduate studies in the UK. S.M.‐L. gratefully acknowledges grant RYC2022‐036510‐I funded by MICIU/AEI/10.13039/501100011033 and by ESF+. M.E.‐E. acknowledges Grant Ref. PID2023‐151780OB‐I00 funded by the Spanish Ministry of Science, Innovation and Universities, MICIU/AEI/10.13039/501100011033 and the European Regional Development Fund (FEDER) from the European Union. ICN2 is funded by the CERCA programme/Generalitat de Catalunya and is supported by the Severo Ochoa Centres of Excellence programme, Grant CEX2021‐001214‐S, funded by MCIU/AEI/10.13039/501100011033. M.S.T.‐O., S.M.‐L. and I.T. are grateful to the European Commission and UK Research and Innovation (UKRI) for their financial support to the SolDAC project funded through Horizon Europe grant agreement 101069359 and Innovation Service Funding 10039331, respectively.

## Conflicts of Interest

The authors declare no conflicts of interest.

## Supporting information


**Supporting File**: advs78274‐sup‐0001‐SuppMat.pdf

## Data Availability

All data generated in this study are provided in the manuscript and its Supplementary Information as tabulated values, parameter values or graphical simulation outputs. The experimental datasets used for model validation were reported previously and are available through the cited publication and its associated Supporting Information [11].
