# Supplementary material for: Product‐Reshaped Gas‐Phase Hydrodynamics Govern CO2 Electrolysis in Gas Diffusion Electrodes
Source: Adv Sci (Weinh). 2026 Oct 14:e78274. Online ahead of print. doi: 10.1002/advs.78274 (PMC13669241; doi:10.1002/advs.78274)
Supplement: Supplementary file 1 [file ADVS-9999-e78274-s001.pdf]

SUPPORTING INFORMATION

Product-reshaped gas-phase hydrodynamics govern CO_2 electrolysis in gas diffusion electrodes

Abdullah A. Alyahya[1,2], Mayra S. Tovar-Oliva[1], Blessings G. Malimusi[1], Sebastián Murcia-López[3], María Escudero-Escribano[3,4], Timm Krüger[5], Ignacio Tudela[1]

[1]School of Engineering, Institute for Materials and Processes, Edinburgh Electrochemical Engineering Group (e3 Group), The University of Edinburgh, Robert Stevenson Road, Edinburgh, EH9 3FB, United Kingdom
[2]Centre of Excellence for Nanomaterials for Clean Energy Applications, King Abdulaziz City for Science and Technology (KACST), King Abdullah Road, Al Raed District, Riyadh, 12354, Saudi Arabia
[3]Catalan Institute of Nanoscience and Nanotechnology (ICN2), Edifici ICN2, CSIC and Barcelona Institute of Science and Technology, UAB Campus, 08193, Bellaterra, Barcelona, Spain
[4]Catalan Institution for Research and Advanced Studies (ICREA), Passeig de Lluís Companys 23, Barcelona, 08010, Spain
[5]School of Engineering, Institute for Multiscale Thermofluidics, The University of Edinburgh, Peter Guthrie Tait Road, Edinburgh, EH9 3FD, United Kingdom

---

Contents

S1 Modelling framework **2**
- S1.1 Purpose and scope . . . 2
- S1.2 Validation hierarchy . . . 3
- S1.3 Assumptions and physical interpretation . . . 3
  - S1.3.1 Treatment of the catalyst layer . . . 4
  - S1.3.2 Gas–liquid transfer length . . . 4
  - S1.3.3 Single-phase electrolyte approximation and hydrogen supersaturation . . . 4
- S1.4 Geometry and operating parameters . . . 5
- S1.5 Gas-phase transport . . . 5
  - S1.5.1 Mass continuity and momentum balance . . . 5
  - S1.5.2 Conservation of gas species . . . 6
- S1.6 Liquid-phase transport . . . 7
  - S1.6.1 Mass continuity and momentum balance . . . 7
  - S1.6.2 Conservation of dissolved species . . . 8
- S1.7 Homogeneous reactions in the electrolyte . . . 8
- S1.8 Conservation of charge . . . 9
- S1.9 Gas–liquid mass transfer . . . 10
- S1.10 Electrochemical reactions and kinetic formulation . . . 10
  - S1.10.1 Reduced reaction network . . . 10
  - S1.10.2 Interpretation and limitations of the reduced kinetic network . . . 11
  - S1.10.3 Butler–Volmer and concentration dependence . . . 11
  - S1.10.4 Equilibrium potentials . . . 13
  - S1.10.5 Kinetic parameters . . . 13
- S1.11 Diagnostic utilisation ratios . . . 14
- S1.12 Boundary conditions . . . 14
- S1.13 Numerical method . . . 16

S2 Additional results **18**
- S2.1 Additional model validation and product-current trends . . . 18
- S2.2 Additional gas-phase composition fields . . . 19
- S2.3 Through-plane gas-phase profiles across the GDL . . . 19
- S2.4 Hydrogen evolution and local supersaturation . . . 20

S2.5 Additional CO-to-product spatial distributions 22
S2.6 Through-plane electrolyte composition profiles 22

S1 Modelling framework

S1.1 Purpose and scope

The modelling framework developed in this work was designed to resolve cathode-side transport and reaction processes during CO_2 electroreduction in a flow reactor equipped with a GDE containing Cu-based catalysts. The model includes the gas flow channel (GFC), gas diffusion electrode (GDE) and electrolyte flow channel (EFC), while the anion exchange membrane (AEM) is represented through boundary conditions at the electrolyte-side boundary. This scope was selected because the main objective of the work was to analyse the coupled cathodic phenomena governing CO_2 availability, CO intermediate formation, gas-phase composition, gas-phase hydrodynamics and C_{2+} product formation.

The model is general in form, but the specific implementation and validation were constructed around a single experimental system for which geometric, operating, electrochemical and transport parameters were available in a sufficiently consistent way [1]. This choice is deliberate. Combining kinetic parameters, transport properties and geometric inputs from different experimental platforms can make model–experiment deviations difficult to interpret. By using data from the same experimental system, the present work establishes a controlled validation context in which agreement for the CO_2-to-CO pathway and deviations for downstream products can be interpreted physically rather than attributed to inconsistent inputs.

The present framework is best viewed as a cathode-side, flow-direction-resolved reactor model. It is designed to capture the reacting gas environment generated during GDE operation: how CO_2 conversion, CO formation and consumption, and gaseous product evolution reshape gas composition, density and velocity along the electrode. This focus is distinct from full-device MEA modelling, whose primary objective would be to resolve membrane transport, water management, crossover, anode-side processes and whole-cell performance. Here, these effects are represented only insofar as they are needed to close the cathode-side transport and charge balances.

A two-dimensional formulation was required because the key phenomena analysed in this study evolve primarily along the flow direction. As CO_2 is consumed and gaseous products are generated, gas composition, density, velocity, CO_2 partial pressure, CO availability and partial current densities progressively change over the electrode length. A one-dimensional through-plane model can resolve local interfacial transport, but it cannot capture this downstream redistribution of reactants, intermediates and products. The through-plane profiles reported confirm that, under the conditions considered here, the gas-phase gradients across the 80 μm GDL are comparatively small relative to the much larger changes that develop along the 25 mm electrode length. This use of spatially resolved flow modelling is consistent with electrochemical reactor-engineering approaches in which hydrodynamics, residence time and transport non-uniformity are treated as part of reactor performance [2].

This reactor-scale objective is also distinct from microstructure-derived transport-property calculations. Such calculations are valuable for relating catalyst-layer morphology to effective diffusivity, permeability or conductivity, but they do not by themselves resolve how electrochemical conversion changes gas composition, density and velocity along the reactor. In the present framework, catalyst-layer morphology enters through effective interfacial and transport parameters, whereas the primary modelling target is the reaction-reshaped cathode gas environment over the electrode length.

S1.2 Validation hierarchy

The validation hierarchy used in the main text is intentionally conservative. The CO_2-to-CO pathway and HER are treated as quantitatively validated because the model reproduces their product-current responses over the relevant potential range. The CO_2-to-CO pathway provides the strongest constraint on the transport–reaction coupling central to the manuscript, because it captures both the apparent kinetic region and the deviation from kinetic behaviour as interfacial CO_2 transport becomes limiting. HER provides an additional quantitative constraint on the gaseous product source term because H_2 formation directly contributes to product-driven changes in gas composition, density and velocity.

The CO-to-C_2H_4 pathway is interpreted at the level of trend and product-dominance agreement. It is used to evaluate whether the model captures the experimentally observed transition from CO-dominated operation toward C_{2+}-dominated operation. The additional CO-derived products, particularly C_2H_5OH and C_3H_7OH, are interpreted qualitatively and at the level of correct order of magnitude. This distinction is necessary because the reduced kinetic network does not resolve all surface intermediates, adsorbate coverages, site competition or branching pathways on Cu.

The contribution of the present model lies in resolving how these effects become coupled during multi-product GDE operation, rather than in any single physical component. Previous studies have examined along-channel gradients in CO_2-to-CO GDEs [3], through-plane MEA transport, crossover and water-management trade-offs [4, 5], Cu-catalysed MEA product-distribution non-uniformity [6], spatially resolved GDE activity measured experimentally [7], CO transport constraints in CO-reduction GDEs [8], and morphology-derived transport properties in catalyst layers [9]. Here, CO_2-to-CO formation, downstream CO consumption, gaseous product evolution and composition-dependent gas-phase hydrodynamics are resolved together along the same electrode in a validated, flow-direction-resolved GDE framework. This distinction is important because the gas phase is not only depleted of CO_2, but is progressively reshaped by gaseous product formation, which changes local density, velocity and reactant delivery.

S1.3 Assumptions and physical interpretation

The model is based on the following assumptions:

- The system is isothermal, with the flow cell operating at constant and uniform temperature. Heat generation and heat transfer associated with electrochemical and homogeneous reactions are therefore neglected [10].
- The gas phase is ideal and compressible, with laminar flow and density variations driven by changes in local gas composition. The low-Mach-number condition is satisfied under the operating conditions considered.
- The electrolyte is treated as an incompressible, single-phase liquid with constant density and viscosity. Gas bubble nucleation and two-phase electrolyte transport are not explicitly resolved.
- The PTFE-based GDL is considered hydrophobic, consistent with the absence of gross flooding in the experimental system used for validation [1].
- The catalyst layer (CL) is treated as effectively wet at the reactor scale. This does not imply that the CL is uniformly filled with electrolyte at the microscopic level, but rather that gas–liquid transfer and electrochemical reactions occur through an effective wetted interfacial region.
- The CL is represented as a boundary between the GDL and electrolyte domain. This avoids explicitly meshing a thin physical layer that is orders of magnitude smaller than the surrounding gas and liquid domains.

- Gravity and other external body forces are neglected because they do not significantly affect the transport and reaction phenomena considered.

S1.3.1 Treatment of the catalyst layer

The GDE used for validation consisted of a PTFE-based GDL, a sputtered Cu film and a porous CL containing mixed Cu oxide particles dispersed in carbon black [1]. The CL thickness is approximately 3.6 μm, whereas the adjacent flow channels are millimetre-scale. Explicitly resolving the CL as a porous domain would therefore require a much finer mesh and would substantially increase computational cost and convergence difficulty. For this reason, the CL was represented as a reactive boundary where gas–liquid transfer, electrochemical reactions and charge transfer occur. Because the CL is represented as an effective boundary, the model does not explicitly resolve spatial variations in gas–liquid interfacial area, pore-scale morphology or dynamic wetting within the catalyst layer. Instead, gas–liquid exchange is represented through an effective interfacial transfer resistance, which enables tractable coupling between gas-phase transport, electrolyte chemistry and electrochemical kinetics at the reactor scale.

This approach is a reactor-scale approximation. It does not attempt to resolve pore-scale gas/liquid distributions, local wetting heterogeneity or microscopic catalyst utilisation within the CL. Instead, it preserves the dominant spatial gradients along the electrode and through the adjacent gas and liquid domains, which are the focus of the present work. This is particularly important because the main conclusions arise from longitudinal changes in CO_2 partial pressure, CO availability, gas velocity and local reaction rates.

S1.3.2 Gas–liquid transfer length

Transport of CO_2, CO, C_2H_4 and H_2 across the gas–liquid interface was described using a thin-film approximation. Previous studies have sometimes assumed very short diffusion lengths corresponding to ideal partially wetted pores [11, 12, 13]. However, the GDE considered here contains a tortuous porous carbon-based CL that undergoes changes in apparent hydrophobicity during operation [1]. For this reason, the effective diffusion length was taken as the CL thickness, $\delta_{G-L} \approx 3.6\ \mu$m.

This value should not be interpreted as a resolved microscopic liquid-film thickness. Instead, it represents an effective gas–liquid transfer resistance of the same order as the unresolved catalyst-layer thickness. In a straight pore, this length corresponds to the order of magnitude of the diffusion distance from the gas–liquid interface to active sites located near the opposite side of the CL. In practice, some active sites may lie closer to the gas–liquid interface, whereas tortuosity, non-straight pores, partial wetting and distributed reaction sites can increase the effective transport path. The use of $\delta_{G-L} \approx 3.6\ \mu$m therefore avoids imposing an unrealistically short gas–liquid diffusion distance relative to the physical thickness and morphology of the catalyst layer, while retaining a tractable effective description of interfacial mass transfer.

This approximation does not resolve the pore-scale gas–liquid interfacial area, local wetting state or distribution of active sites. However, it captures the dominant reactor-scale limitation associated with interfacial CO_2 supply and is consistent with the ability of the model to reproduce the CO_2-to-CO pathway across kinetic and transport-limited regimes.

S1.3.3 Single-phase electrolyte approximation and hydrogen supersaturation

The electrolyte was modelled as a single liquid phase. At the most negative cathode potentials, the predicted dissolved H_2 concentration near the electrode surface exceeds the equilibrium solubility expected under comparable conditions [14]. In practice, such supersaturation would likely promote hydrogen bubble nucleation and disengagement. These two-phase phenomena were not explicitly included because the present work focuses on the coupled gas-side and interfacial transport processes controlling carbon selectivity, and because adding two-phase electrolyte transport would substantially increase model complexity and computational cost.

The present framework nevertheless captures the influence of HER on gas composition and gas-side hydrodynamics through the generation of H_2 at the GDE/electrolyte interface. A future extension including two-phase electrolyte transport would be valuable for resolving bubble formation, liquid displacement and possible local changes in effective transport near the electrode.

S1.4 Geometry and operating parameters

The simulated geometry represents the cathodic compartment of a flow electrochemical reactor equipped with a GDE and an AEM. The geometry consisted of three explicitly resolved domains: the gas flow channel (GFC), gas diffusion layer (GDL) and electrolyte flow channel (EFC). The CL was represented as a reactive boundary between the GDL and EFC, while the electrolyte-facing surface of the AEM was represented through boundary conditions. The simplified cell architecture, coordinate system and corresponding two-dimensional model section are shown in Figure 1 of the main manuscript. The main geometric, operating and material parameters are listed in Table S1.

S1.5 Gas-phase transport

S1.5.1 Mass continuity and momentum balance

The gas phase in the gas flow channel was described using the compressible Navier–Stokes equations under isothermal laminar-flow conditions. Mass continuity is given by

$$\frac{\partial \rho}{\partial t} + \nabla \cdot (\rho \mathbf{u}) = 0, \tag{S1}$$

where ρ is the gas density and $\mathbf{u}$ is the free-flow gas velocity in the gas flow channel. Momentum conservation is described by

$$\rho \frac{\mathrm{D}\mathbf{u}}{\mathrm{D}t} = -\nabla p + \nabla \cdot \boldsymbol{\tau}, \tag{S2}$$

where p is pressure and $\boldsymbol{\tau}$ is the viscous stress tensor. The material derivative and stress tensor are

$$\frac{\mathrm{D}\mathbf{u}}{\mathrm{D}t} = \frac{\partial \mathbf{u}}{\partial t} + \mathbf{u} \cdot \nabla \mathbf{u}, \tag{S3}$$

and

$$\boldsymbol{\tau} = \mu \left(\nabla \mathbf{u} + (\nabla \mathbf{u})^{\top} \right) - \frac{2}{3} \mu (\nabla \cdot \mathbf{u}) \mathbf{I}. \tag{S4}$$

The gas phase was closed using an ideal gas equation of state. The gas viscosity was updated from the local gas composition using the Wilke method [17].

Flow through the porous GDE was described using Brinkman equations. In the porous GDE, $\mathbf{u}$ denotes the superficial, or Darcy, gas velocity averaged over the total cross-sectional area of the porous medium consistent with the porous-flow formulation used in COMSOL. The corresponding interstitial gas velocity is $\mathbf{u}/\varepsilon_{\mathrm{GDE}}$. The porous-domain continuity and momentum balances are

$$\frac{\partial}{\partial t} (\varepsilon_{\mathrm{GDE}} \rho) + \nabla \cdot (\rho \mathbf{u}) = 0, \tag{S5}$$

and

$$\frac{\rho}{\varepsilon_{\mathrm{GDE}}} \left[\frac{\partial \mathbf{u}}{\partial t} + (\mathbf{u} \cdot \nabla) \left(\frac{\mathbf{u}}{\varepsilon_{\mathrm{GDE}}} \right) \right] = -\nabla p + \nabla \cdot \left(\frac{1}{\varepsilon_{\mathrm{GDE}}} \boldsymbol{\tau} \right) - \frac{\mu}{\kappa_{\mathrm{GDE}}} \mathbf{u}, \tag{S6}$$

where $\varepsilon_{\mathrm{GDE}}$ and κ_{GDE} are the porosity and permeability of the GDE, respectively. No volumetric mass source was applied in either the gas flow channel or the porous GDE. Gas consumption, generation and gas–liquid exchange were imposed through interfacial fluxes at the catalyst-layer boundary.

TABLE S1 | Input model parameters.

**Parameter**	**Value**	**Reference**
Dimensions of cathode compartment		
Height	25 mm	[1]
Width	20 mm	[1]
EFC thickness	4 mm	[1]
EFC inlet thickness	2 mm	[1]
EFC outlet thickness	2 mm	[1]
GFC thickness	3 mm	[1]
GFC inlet thickness	2 mm	[1]
GFC outlet thickness	2 mm	[1]
GDL thickness	80 μm	[1]
CL thickness	3.6 μm	[1]
Operating conditions		
Temperature	25°C	[1]
Pressure	1 atm	[1]
Gas conditions/properties		
Inlet flow rate	12 ml min^{-1}	[1]
Inlet CO_2 purity	100%	[1]
Density	1.98 kg m^{-3}	[13]
Dynamic viscosity	1.5×10^{-5} Pa s	[13]
Electrolyte conditions/properties		
Inlet flow rate	15 ml min^{-1}	[1]
Inlet KOH concentration	1 M	[1]
Density	1046 kg m^{-3}	[15]
Dynamic viscosity	0.00099 Pa s	[16]
GDE properties		
GDL porosity	0.5	assumed
GDL pore size	0.45 μm	[1]
GDL tortuosity	1.5	assumed
GDL permeability	8.4×10^{-12} m^2	[1]
GDL conductivity	2×10^7 S m^{-1}	[1]
CL porosity	0.5	assumed
CL tortuosity	1.5	assumed
AEM properties		
Thickness (dry)	50 μm	[1]
Weight per unit area	7.25 mg cm^{-2}	[1]
Ion exchange capacity	1.85 mmol g^{-1}	[1]
Water uptake	17.5%	[1]

This homogenised porous-medium representation does not resolve pore-scale heterogeneity or phase-dependent permeability effects, but captures the dominant coupling between gas flow, gas composition and electrochemical reaction at the electrode scale.

S1.5.2 Conservation of gas species

The conservation equation for gas species i was written in terms of mass fraction:

$$\frac{\partial(\rho\omega_i)}{\partial t} + \nabla \cdot (\rho\omega_i \mathbf{u}) = -\nabla \cdot \mathbf{j}_i. \tag{S7}$$

where ω_i is the gas-phase mass fraction of species i and $\mathbf{j}_i$ is its diffusive mass flux. Using mass continuity, this can be written as

$$\rho \frac{\mathrm{D}\omega_i}{\mathrm{D}t} = -\nabla \cdot \mathbf{j}_i. \tag{S8}$$

The diffusive mass flux was obtained from a Maxwell–Stefan description of multicomponent diffusion:

$$\sum_{k \neq i} \frac{x_i x_k}{D_{ik}} (\mathbf{v}_k - \mathbf{v}_i) = \mathbf{d}_i, \tag{S9}$$

where D_{ik} is the binary diffusivity, estimated using the Fuller method [18], and $\mathbf{d}_i$ is the diffusional driving force. After inversion of the Maxwell–Stefan system, the mass flux can be written as

$$\mathbf{j}_i = -\rho \omega_i \sum_{k=1}^{N} \tilde{D}_{ik} \mathbf{d}_k. \tag{S10}$$

For ideal gas mixtures,

$$\mathbf{d}_i = \nabla x_i + \frac{x_i - \omega_i}{p} \nabla p. \tag{S11}$$

Within the porous GDE, where $\mathbf{u}$ is the superficial gas velocity, the porosity-corrected conservation equation becomes

$$\varepsilon_{\mathrm{GDE}} \rho \frac{\partial \omega_i}{\partial t} + \rho \mathbf{u} \cdot \nabla \omega_i = -\nabla \cdot \mathbf{j}_i. \tag{S12}$$

Interactions between gas molecules and pore walls were included through the porous-media Maxwell–Stefan formulation:

$$\sum_{k \neq i} \frac{x_i x_k}{D_{ik}^{\mathrm{eff}}} (\mathbf{v}_k - \mathbf{v}_i) - \frac{x_i}{D_i^{\mathrm{W,eff}}} (\mathbf{v}_i + \mathbf{u}_{\mathrm{W}}) = \mathbf{d}_i. \tag{S13}$$

The effective binary and Knudsen diffusivities were estimated using the Bruggeman correction:

$$D_{ik}^{\mathrm{eff}} = \varepsilon_{\mathrm{GDE}}^{1.5} D_{ik}, \qquad D_i^{\mathrm{W,eff}} = \varepsilon_{\mathrm{GDE}}^{1.5} D_i^{\mathrm{W}}. \tag{S14}$$

The Knudsen diffusivity was calculated as

$$D_i^{\mathrm{W}} = \frac{d_p}{3} \sqrt{\frac{8RT}{\pi M_i}}, \tag{S15}$$

where d_p is the pore diameter and M_i is the molar mass of species i.

S1.6 Liquid-phase transport

S1.6.1 Mass continuity and momentum balance

The electrolyte was treated as an incompressible laminar liquid phase:

$$\nabla \cdot \mathbf{u} = 0. \tag{S16}$$

The electrolyte momentum balance was described using the incompressible form of Equations S2–S4, with density and viscosity values listed in Table S1. Water losses through evaporation, flooding or electro-osmotic drag were neglected in the cathode-side domain.

S1.6.2 Conservation of dissolved species

The mass balance for dissolved species i in the electrolyte was

$$\frac{\partial c_i}{\partial t} + \nabla \cdot \mathbf{N}_i = S_i, \tag{S17}$$

with total molar flux

$$\mathbf{N}_i = -D_i \nabla c_i - z_i u_{m_i} F c_i \nabla \phi_{\mathrm{L}} + c_i \mathbf{u}. \tag{S18}$$

The first, second and third terms correspond to diffusion, migration and convection, respectively. The mobility u_{m_i} was defined using the Einstein relation, $u_{m_i} = D_i/(RT)$. The diffusion–migration flux is

$$\mathbf{J}_i = -D_i \nabla c_i - z_i u_{m_i} F c_i \nabla \phi_{\mathrm{L}}. \tag{S19}$$

For incompressible flow, the species balance becomes

$$\frac{\partial c_i}{\partial t} + \mathbf{u} \cdot \nabla c_i + \nabla \cdot \mathbf{J}_i = S_i. \tag{S20}$$

S1.7 Homogeneous reactions in the electrolyte

Dissolved CO_2 undergoes homogeneous reactions with water and hydroxide, forming bicarbonate and carbonate species. The carbonate–water chemistry was represented by the following formal reversible reactions:

- Reaction 1 (R1): $CO_2 + H_2O \underset{k_{r,1}}{\overset{k_{f,1}}{\rightleftharpoons}} HCO_3{}^- + H^+$
- Reaction 2 (R2): $CO_2 + OH^- \underset{k_{r,2}}{\overset{k_{f,2}}{\rightleftharpoons}} HCO_3{}^-$
- Reaction 3 (R3): $HCO_3{}^- \underset{k_{r,3}}{\overset{k_{f,3}}{\rightleftharpoons}} CO_3{}^{2-} + H^+$
- Reaction 4 (R4): $HCO_3{}^- + OH^- \underset{k_{r,4}}{\overset{k_{f,4}}{\rightleftharpoons}} CO_3{}^{2-} + H_2O$
- Water dissociation (W): $H_2O \overset{K_w}{\rightleftharpoons} H^+ + OH^-$

These reactions are necessary because local CO_2 absorption, carbonate formation and OH^- generation jointly determine the local electrolyte composition and pH. In the numerical implementation, R1, R2 and R3 were solved as finite-rate homogeneous reactions, while W was included through the water-dissociation equilibrium. Although the carbonate–water reaction network has stoichiometric rank three, the finite-rate implementation was not reduced simply to a minimal stoichiometric basis. R2 was retained because it is kinetically important under strongly alkaline conditions and dominates homogeneous CO_2 consumption. R1 and R3 were retained to preserve the direct hydration and bicarbonate/carbonate acid–base pathways in a numerically tractable form. The direct bicarbonate–hydroxide pathway R4 is shown for chemical completeness, but was not implemented as an independent finite-rate source term because it is stoichiometrically dependent on R3 and W and introduced severe numerical stiffness in COMSOL. Its equilibrium effect is retained through the coupled R3 chemistry, water-dissociation equilibrium W, electroneutrality and the local H^+/OH^- balance.

The equilibrium constant for each reversible reaction j is

$$K_j = \frac{k_{j,\mathrm{f}}}{k_{j,\mathrm{r}}}. \tag{S21}$$

Using the law of mass action, the net reaction rate was written as

$$R_j = k_{j,\mathrm{f}} \prod_{i\in\mathrm{reactants}} c_i^{|\nu_{j,i}|} - k_{j,\mathrm{r}} \prod_{i\in\mathrm{products}} c_i^{|\nu_{j,i}|}. \tag{S22}$$

For reactions where the forward rate constant and equilibrium constant were specified, the reverse rate constant was calculated as $k_{j,\mathrm{r}} = k_{j,\mathrm{f}}/K_j$. The equilibrium constants for the stoichiometrically dependent alkaline pathways are related to the acid–base equilibria according to $K_2 \approx K_1/K_w$ and $K_4 \approx K_3/K_w$, with small differences arising from rounding, concentration conventions and the literature sources used for the individual constants. The units reported in Table S2 therefore correspond to the concentration-based definitions used in the kinetic expressions.

TABLE S2 | Equilibrium and rate constants for the homogeneous carbonate–water reactions at T = 298.15 K. R1, R2 and R3 were implemented as finite-rate source terms, while W was included through the water-dissociation equilibrium. R4 is shown for chemical completeness but was not included as an independent finite-rate source term in the numerical model.

Reaction	Parameter	Value	Units	Reference
R1	K_1	1×10^{-6}	M	Calculated
	$k_{f,1}$	3.71×10^{-2}	s^{-1}	[19]
	$k_{r,1}$	3.71×10^{4}	$M^{-1}\,s^{-1}$	Calculated
R2	K_2	1×10^{8}	M^{-1}	[20]
	$k_{f,2}$	1×10^{3}	$M^{-1}\,s^{-1}$	[20]
	$k_{r,2}$	1×10^{-5}	s^{-1}	Calculated
R3	K_3	5×10^{-11}	M	Calculated
	$k_{f,3}$	2.5	s^{-1}	[21]
	$k_{r,3}$	5×10^{10}	$M^{-1}\,s^{-1}$	[21]
R4†	K_4	4.66×10^{3}	M^{-1}	[22]
	$k_{f,4}$	1×10^{8}	$M^{-1}\,s^{-1}$	[22]
	$k_{r,4}$	2.15×10^{4}	s^{-1}	[22]
W	K_w	1×10^{-14}	M^2	[23]

†R4 was not implemented as an independent finite-rate source term because it is stoichiometrically dependent on R3 and W and introduced severe numerical stiffness in COMSOL. Its equilibrium constant is thermodynamically related to the implemented equilibria by $K_4 \approx K_3/K_w$; the tabulated value reflects the rounded, literature-sourced constants used in the model.

S1.8 Conservation of charge

Charge transport within the GDE was governed by Ohm's law:

$$\mathbf{i}_{\mathrm{GDE}} = -\sigma_{\mathrm{GDE}} \nabla \phi_{\mathrm{GDE}}. \tag{S23}$$

Charge conservation gives

$$\nabla \cdot \mathbf{i}_{\mathrm{GDE}} = Q_{\mathrm{GDE}}. \tag{S24}$$

In the electrolyte, electroneutrality was assumed:

$$\sum_i z_i c_i = 0. \tag{S25}$$

The electrolyte current density was calculated as

$$\mathbf{i}_{\mathrm{L}} = F \sum_i z_i \mathbf{J}_i. \tag{S26}$$

Charge conservation in the electrolyte was

$$\nabla \cdot \mathbf{i}_L = Q_L. \tag{S27}$$

At the GDE/electrolyte interface, charge transfer was imposed through

$$\nabla \cdot \mathbf{i}_{GDE} = -\nabla \cdot \mathbf{i}_L = -a_{v,GDE} \sum_j i_j. \tag{S28}$$

S1.9 Gas–liquid mass transfer

Transport of CO_2, CO, C_2H_4 and H_2 across the gas–liquid interface was described using a thin-film approximation based on film theory [13, 24]:

$$J_{i,G-L} = -D_{i,G-L} \frac{dc_{i,G-L}}{dx} \approx -D_{i,G-L} \frac{c^t_{i,G-L} - c^*_{i,G-L}}{\delta_{G-L}}. \tag{S29}$$

At the gas–liquid interface, Henry's law was used to define $c^*_{i,G-L} = p_i H_i$. The effective film thickness was taken as $\delta_{G-L} \approx 3.6\ \mu$m, corresponding to the CL thickness. This value of δ_{G-L} is larger than the film thicknesses commonly assumed for ideal partially wetted pores, but it is more consistent with the fairly wet and tortuous catalyst layer considered here.

The effective diffusivity in the interfacial film was estimated with the Bruggeman correction.

$$D_{i,G-L} = \varepsilon_{CL}^{1.5} D_i. \tag{S30}$$

This approximation does not explicitly resolve spatial variations in gas–liquid interfacial area, pore-scale morphology or dynamic wetting, but provides an effective interfacial transport resistance that can be coupled to gas-phase transport, electrolyte chemistry and electrochemical kinetics.

S1.10 Electrochemical reactions and kinetic formulation

S1.10.1 Reduced reaction network

The electrochemical network was represented through a reduced set of lumped reactions:

- $CO_2 + H_2O + 2\,e^- \longleftrightarrow CO + 2\,OH^-$
- $2\,CO + 3\,H_2O + 4\,e^- \longleftrightarrow CH_3COO^- + 3\,OH^-$
- $2\,CO + 6\,H_2O + 8\,e^- \longleftrightarrow C_2H_4 + 8\,OH^-$
- $2\,CO + 7\,H_2O + 8\,e^- \longleftrightarrow C_2H_5OH + 8\,OH^-$
- $3\,CO + 10\,H_2O + 12\,e^- \longleftrightarrow C_3H_7OH + 12\,OH^-$
- $2\,H_2O + 2\,e^- \longleftrightarrow H_2 + 2\,OH^-$

This reduced network captures the main product families observed experimentally while allowing the model to resolve the reactor-scale coupling between CO_2 transport, CO intermediate availability and multi-product selectivity. CH_4 was not included because it was not observed as a significant product in the experimental dataset used for validation; the reduced network was therefore restricted to the dominant measured carbon products instead of attempting to explicitly resolve all adsorbed intermediates or elementary reaction steps on Cu-based surfaces [25, 26].

S1.10.2 Interpretation and limitations of the reduced kinetic network

The electrochemical reaction network used in this work should be interpreted as a reduced kinetic representation rather than as a microkinetic mechanism. The reactions listed above correspond to lumped overall pathways for HER, CO_2-to-CO and CO-to-product formation. They do not represent elementary steps and therefore do not explicitly resolve adsorbed intermediates, coverage effects, site competition, potential-dependent changes in rate-determining steps, or separate branching pathways for different C_{2+} products.

This limitation is particularly relevant for the quantitative prediction of individual CO-derived products, especially alcohols, whose formation is known to involve multiple surface intermediates and competing pathways. For this reason, the validation hierarchy adopted in the manuscript is deliberately conservative: the CO_2-to-CO pathway and HER are interpreted as quantitatively validated over the relevant potential range, CO-to-C_2H_4 is interpreted at the level of trend and product-dominance agreement, and the remaining CO-to-C_{2+} pathways are interpreted qualitatively or at the level of correct order of magnitude.

Importantly, the main conclusions of the study do not rely on the reduced kinetic network providing a complete microkinetic description of Cu-based CO_2 reduction. The central findings concern reactor-scale coupling: the transition of the CO_2-to-CO pathway from kinetic to interfacial transport control, the resulting changes in CO availability, and the feedback between product formation, gas composition and gas-phase hydrodynamics. These effects arise from conservation of mass, multicomponent transport, gas–liquid transfer and Faradaic coupling, and therefore remain meaningful even though the individual CO-to-product pathways are described using lumped kinetics.

A more detailed microkinetic treatment could improve quantitative prediction of the distribution among CH_3COO^-, C_2H_4, C_2H_5OH and C_3H_7OH, particularly under conditions where surface coverage or adsorbate competition becomes dominant. Such an extension would be valuable in future work, but it would also require additional experimentally constrained kinetic parameters that are not currently available for the specific GDE system considered here.

S1.10.3 Butler–Volmer and concentration dependence

The practical Butler–Volmer form used for electrochemical reaction j was [27]

$$i_j = i^e_{j,\mathrm{ref}} \left[g_{j,\mathrm{a}} \exp\left(\frac{\alpha_{j,\mathrm{a}} F}{RT}\eta_j\right) - g_{j,\mathrm{c}} \exp\left(-\frac{\alpha_{j,\mathrm{c}} F}{RT}\eta_j\right)\right]. \tag{S31}$$

For the cathodic conditions considered in this work, this expression was simplified to

$$i_j \approx -i^e_{j,\mathrm{ref}} g_{j,\mathrm{c}} \exp\left(-\frac{\alpha_{j,\mathrm{c}} F}{RT}\eta_j\right). \tag{S32}$$

In the practical Butler–Volmer formulation, the exchange current density, overpotential and concentration-dependent factors are all defined relative to chosen reference conditions [27]. The concentration factors are therefore not neutral normalising terms. At the reference state, they must recover unity; otherwise, the fitted value of $i^e_{j,\mathrm{ref}}$ necessarily absorbs the penalty associated with the chosen concentration normalisation. This point is especially important when kinetic parameters are obtained by fitting apparent Tafel regions. If $g_{j,\mathrm{c}}$ is substantially smaller than unity in the potential range interpreted as kinetic control, the fitted kinetic prefactor is no longer separable from the arbitrary reference concentration used in the concentration term.

This issue is non-trivial for gas-fed CO_2 electroreduction in alkaline GDEs. The relevant reactants, CO_2 and CO, are not supplied from a well-defined bulk dissolved reservoir. Their local concentrations depend on gas composition, pressure, gas–liquid transfer, electrolyte chemistry, homogeneous carbonate reactions, position along the electrode and the extent of electrochemical conversion. A fixed reference concentration such as 1 M is therefore not representative of the dissolved CO_2 concentrations expected in this environment. Even

the aqueous solubility of CO_2 under acidic or unbuffered conditions, approximately 33 mM at 1 atm, is not an appropriate universal reference state in strongly alkaline electrolyte, where free dissolved CO_2 is rapidly converted into bicarbonate and carbonate species. Such reference choices can still be made empirically, but the resulting kinetic prefactors then depend on that choice and should not be interpreted independently of it.

This ambiguity is reflected in the existing continuum-modelling literature. In some studies, the reference concentration used for CO_2 in the cathodic concentration term is not explicitly reported [4, 28, 29, 30]. In others, $c_{CO_2,\mathrm{ref}}$ has been set to 1 M [11, 12, 13, 5, 31], which is much larger than the aqueous solubility of CO_2 at 1 atm. Values closer to the Henry-law solubility of CO_2 in water, approximately 33 mM, have also been used [32, 33, 34]. These choices may be defensible within specific empirical parameterisations, but they are not interchangeable. In alkaline GDE operation, free dissolved CO_2 is rapidly redistributed through bicarbonate/carbonate chemistry, while the local reactant concentration at the catalyst layer varies with gas composition, interfacial transfer and position along the electrode. The selected concentration normalisation therefore affects not only the numerical value of the fitted kinetic prefactor, but also its physical interpretation and comparability across models. Unless the concentration factor recovers $g_{j,\mathrm{c}} \approx 1$ in the apparent kinetic region used for fitting, the fitted kinetic parameter necessarily includes an implicit penalty associated with the chosen reference concentration.

The concentration terms in Equation S31 can also, in principle, include empirical reaction orders or stoichiometric dependencies [27, 35, 36]. However, the reactions used here are lumped overall pathways rather than elementary steps. Imposing the full stoichiometric orders of the overall reactions would therefore not be mechanistically justified, particularly for CO-derived products whose formation involves multiple adsorbed intermediates, branching pathways and active-site requirements.

For these reasons, the cathodic concentration term was defined as a bounded local availability factor:

$$g_{j,\mathrm{c}} = \prod_{i \in \mathrm{O}} \frac{c_i}{c_i + c_i^*}. \tag{S33}$$

Here, c_i is the local concentration of reactant i at the catalyst-layer boundary, O denotes the oxidised-side reactants in the corresponding cathodic pathway, and c_i^* is a threshold concentration that sets the scale at which local reactant availability begins to affect the rate. The parameter c_i^* should not be interpreted as a universal standard concentration. Instead, it defines the transition between a local non-limiting state, where $c_i \gg c_i^*$ and $g_{j,\mathrm{c}} \approx 1$, and an availability-limited state, where c_i becomes comparable to or smaller than c_i^* and $g_{j,\mathrm{c}}$ decreases smoothly toward zero.

This formulation preserves the reference-state meaning of the practical Butler–Volmer expression. In the apparent kinetic region used to fit the kinetic parameters, the relevant reactants remain locally available and $g_{j,\mathrm{c}} \approx 1$, so the fitted $i^e_{j,\mathrm{ref}}$ is not forced to compensate for an arbitrary concentration-reference penalty. As the local concentration decreases, the same expression introduces a smooth reduction in the reaction rate associated with local reactant availability, without imposing unjustified full stoichiometric reaction orders on lumped product-forming pathways.

For both CO_2 and CO, $c_i^* = 0.33$ mM was used. This value is two orders of magnitude lower than the solubility of CO_2 in water under acidic or unbuffered conditions at 1 atm, but is consistent with the low free CO_2 concentrations expected near alkaline interfaces where absorbed CO_2 is rapidly redistributed into bicarbonate and carbonate species. The same threshold was used for CO because, in the present CO_2-fed system, local CO availability is generated upstream by CO_2-to-CO conversion and should not be assigned a substantially larger non-limiting concentration scale than the CO_2 supply from which it derives.

Finally, Equation S33 is not used as a hard conservation constraint. Under severe CO_2 limitation, the physically relevant upper bound is instead set by the interfacial CO_2 flux and the corresponding Faradaic limiting current, as defined below. Similarly, the availability of CO for downstream C_{2+} formation is evaluated through the CO usage ratio. The bounded concentration term therefore regularises the transition from apparent kinetic control to local availability limitation, while the utilisation diagnostics provide the mass-balance interpretation of the

transport-limited regimes.

S1.10.4 Equilibrium potentials

The overpotential for reaction j was defined as

$$\eta_j = E - E^e_{j,\mathrm{ref}}. \tag{S34}$$

The equilibrium potential under reference conditions was estimated from Nernst-type relations:

$$E^e_{j,\mathrm{ref}} = E^\circ_j - \frac{RT}{n_j F} \ln Q_{j,\mathrm{ref}}. \tag{S35}$$

For HER in alkaline electrolyte,

$$E^e_{\mathrm{H_2O/H_2,ref}} \approx E^\circ_{\mathrm{H_2O/H_2}} - \frac{RT}{2F} \ln a^2_{\mathrm{OH^-}}, \tag{S36}$$

which at 298.15 K gives

$$E^e_{\mathrm{H_2O/H_2,ref}} \approx -0.059\, pH \quad \text{V vs SHE}. \tag{S37}$$

The equilibrium potentials used in the model are listed in Table S3. Full Nernst-type expressions were also evaluated during the simulations as quality checks, but the HER-referenced formulation was used for the kinetic calculations because it improved numerical stability and did not significantly change the resulting equilibrium potentials.

TABLE S3 | Equilibrium potential under chosen reference conditions for electrochemical half reactions at $T = 298.15$ K.

Electrochemical half reaction	$E^e_{j,\mathrm{ref}}$ V *vs* SHE
H_2O/H_2	$-0.059\, pH$
CO_2/CO	$-0.059\, pH - 0.100$
CO/CH_3COOH	$-0.059\, pH + 0.320$
CO/C_2H_4	$-0.059\, pH + 0.185$
CO/C_2H_5OH	$-0.059\, pH + 0.170$
CO/C_3H_7OH	$-0.059\, pH + 0.200$

S1.10.5 Kinetic parameters

TABLE S4 | Kinetic parameters used for HER, CO_2-to-CO and CO-to-C_{2+} products over the GDE used for validation [1].

Electrochemical half reaction	$\alpha_{j,\mathrm{c}}$	$i^e_{j,\mathrm{ref}}$ mA cm^{-2}
H_2O/H_2	0.3806	4.141×10^{-3}
CO_2/CO	0.4668	2.151×10^{-2}
CO/CH_3COOH	0.4359	3.148×10^{-7}
CO/C_2H_4	0.8806	1.305×10^{-9}
CO/C_2H_5OH	0.5470	1.634×10^{-6}
CO/C_3H_7OH	0.5313	1.380×10^{-6}

Kinetic parameters were obtained by fitting the electrochemical model to experimental data acquired under apparent kinetic control for the GDE used for validation [1]. The fitting was carried out using a generalised reduced gradient method, with residuals weighted to minimise both vertical and perpendicular offsets [37].

S1.11 Diagnostic utilisation ratios

The limiting current density for the CO_2-to-CO pathway was estimated from a mass balance at the surface of the GDE by combining the interfacial CO_2 flux in Equation S29 with Faraday's Laws of Electrolysis [38, 39]:

$$i_{CO_2/CO,\mathrm{lim}} = -\frac{n_{CO_2/CO} F J_{CO_2,G-L}}{|\nu_{CO_2,CO_2/CO}|}. \tag{S38}$$

The dimensionless CO_2 utilisation ratio was then defined as

$$X_{CO_2} = \frac{|i_{CO_2/CO}|}{|i_{CO_2/CO,\mathrm{lim}}|} \leq 1. \tag{S39}$$

When $X_{CO_2} \ll 1$, only a small fraction of the CO_2 crossing the gas–liquid interface is electrochemically consumed. When $X_{CO_2} \approx 1$, most of the transported CO_2 is consumed to form CO, indicating that the pathway is approaching interfacial mass transport control.

The CO usage ratio was defined from a local CO mass balance using again Faraday's Laws of Electrolysis [38, 39]:

$$X_{CO} = \frac{\sum_k \frac{|\nu_{CO/k}|\,|j_{CO/k}|}{n_{CO/k} F}}{\frac{|\nu_{CO,CO_2/CO}|\,|j_{CO_2/CO}|}{n_{CO_2/CO} F}} \leq 1. \tag{S40}$$

This ratio compares the rate of CO consumption toward C_{2+} products with the rate of CO generation from CO_2.

All three parameters, $i_{CO_2/CO,\mathrm{lim}}$, X_{CO_2} and X_{CO}, were used as diagnostic quantities and were not imposed as hard constraints in the numerical solution.

S1.12 Boundary conditions

Table S5 summarises the boundary conditions used in this study and their mathematical definitions, while Figure S1 shows their placement within the modelling domains.

At the GFC inlet (boundary ①), pure CO_2 was supplied at the experimental gas flow rate of 12 ml min^{-1}, whereas the GFC outlet (boundary ②) was assigned atmospheric pressure and zero diffusive flux for the gas species. At the EFC inlet (boundary ③), a 1 M KOH electrolyte was supplied at the experimental liquid flow rate of 15 ml min^{-1}, while the EFC outlet (boundary ④) was similarly defined using atmospheric pressure and zero diffusive flux for dissolved species. The gas-facing side of the GDE (boundary ⑤) was treated as the electronically conducting electrode boundary, where the applied cathode potential was prescribed. The GDE/electrolyte interface (boundary ⑥) represents the reactive boundary of the cathode. At this boundary, gas–liquid transfer, electrochemical reactions and interfacial charge transfer were coupled. This boundary therefore connects the gas-phase transport problem in the GDE with the electrolyte transport and reaction problem in the EFC. Within the EFC, the boundary corresponding to the experimental reference-electrode location (boundary ⑦) was used to define the electrolyte potential reference. The opposite side of the EFC (boundary ⑧) represents the electrolyte-facing surface of the AEM. Because membrane transport and the anodic compartment were not explicitly resolved in this cathode-side model, this boundary was treated as an ionically saturated interface that allows the cathodic charge balance to be closed without resolving through-membrane species transport. All remaining external walls were treated as no-slip and no-flux boundaries, with electrical insulation where appropriate.

TABLE S5 | Boundary conditions for flow, transport of species, electrochemical reactions and charge conservation as numbered in Figure S1.

Boundary	Description	Conditions	Equation
①	GFC inlet	Pure CO_2	$\omega_{CO_2} = 1$
		Flowrate = 12 ml min^{-1}; uniform velocity normal to boundary	$\mathbf{u} \cdot \mathbf{n} = 0.005$ m s^{-1}, $\mathbf{u} \cdot \mathbf{t} = 0$
②	GFC outlet	Constant atmospheric pressure	$p = 1$ atm
		Zero-diffusive flux of species	$\mathbf{n} \cdot (-\rho D_i \nabla \omega_i) = 0$
③	EFC inlet	1 M KOH aqueous electrolyte	$c_{K^+} = 1$ M, $c_{OH^-} = 1$ M
		Flow rate = 15 ml min^{-1}; uniform velocity normal to boundary	$\mathbf{u} \cdot \mathbf{n} = 0.00625$ m s^{-1}, $\mathbf{u} \cdot \mathbf{t} = 0$
④	EFC outlet	Constant atmospheric pressure	$p = 1$ atm
		Zero-diffusive flux of species	$\mathbf{n} \cdot (-D_i \nabla c_i) = 0$
⑤	GDE gas-facing side	Applied cathode potential	$\phi_{GDE} = -0.4$ to -0.75 V *vs* RHE
⑥	GDE/electrolyte interface	Slip wall condition	$\mathbf{u} \cdot \mathbf{n} = 0$, $\mathbf{t} \cdot \boldsymbol{\tau} \cdot \mathbf{n} = 0$
		Gas–liquid transfer	Equation S29
		Reaction kinetics	Equation S32
		Applied potential at surface	$E = \phi_{GDE} - \phi_L$
		Interfacial charge transfer	Equation S28
⑦	Reference electrode location	SHE potential	$\phi_L = 0$ V *vs* SHE
⑧	Electrolyte–AEM interface	Saturated with charge-carrying species	$c_{CO_3^{2-}} \approx 1$ M
		AEM surface potential	$\phi_{AEM} = \phi_L$
⑨–⑬	External GFC walls	No-slip wall condition	$\mathbf{u} = \mathbf{0}$
		No flux	$\mathbf{n} \cdot (\mathbf{j}_i + \rho \omega_i \mathbf{u}) = 0$
		Charge insulation	$\mathbf{n} \cdot \mathbf{i}_{GDE} = 0$
⑭–⑰	External EFC walls	No-slip wall condition	$\mathbf{u} = \mathbf{0}$
		No flux	$\mathbf{n} \cdot \mathbf{N}_i = 0$
		Charge insulation	$\mathbf{n} \cdot \mathbf{i}_L = 0$

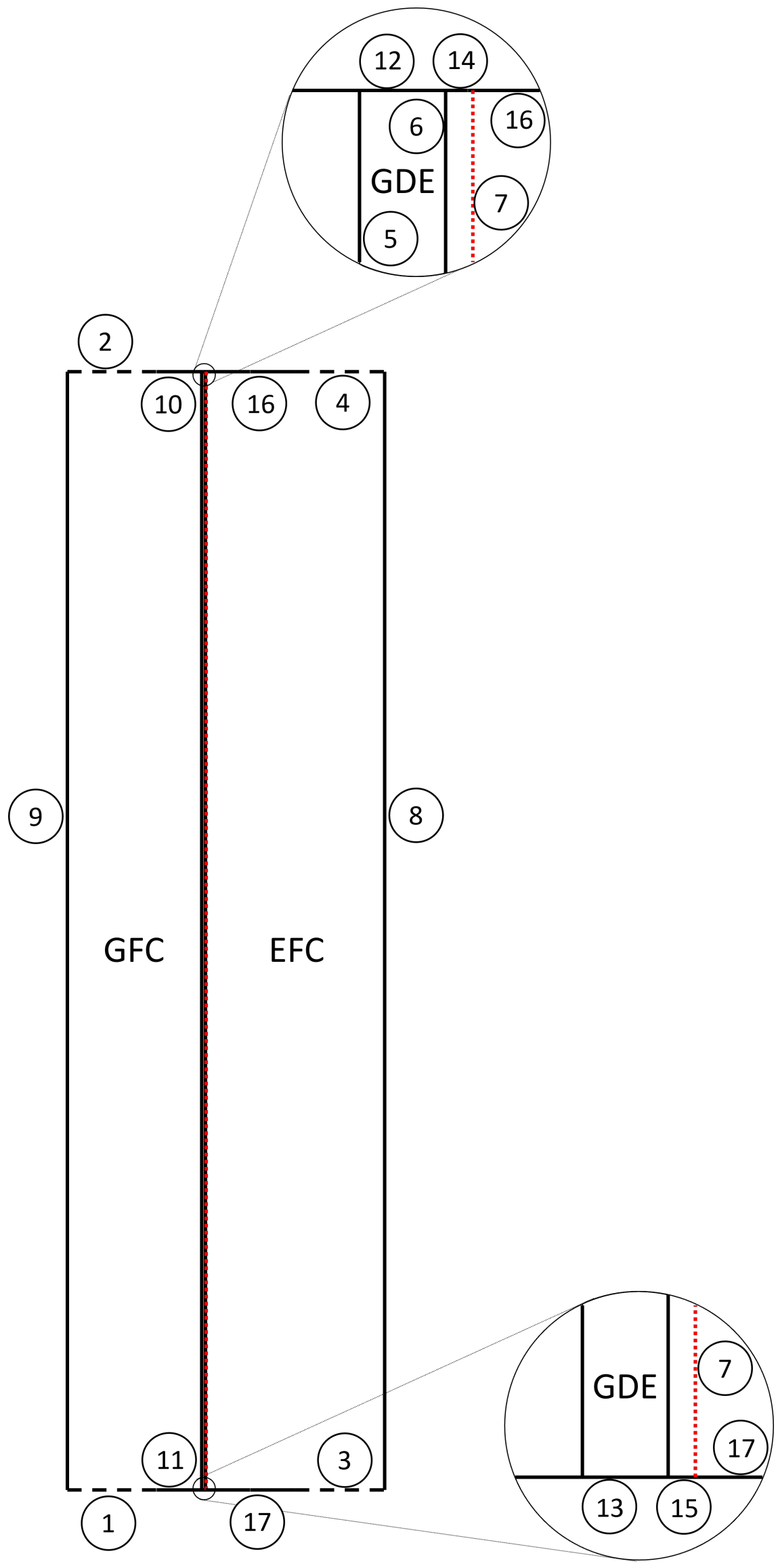

FIGURE S1 | Boundary-condition map for the two-dimensional cathode-side model domain. Boundary numbers correspond to the flow, species-transport, electrochemical and charge-conservation conditions listed in Table S5.

S1.13 Numerical method

The geometry, governing equations and boundary conditions were implemented in COMSOL Multiphysics 6.2. The gas-phase porous-media transport was solved using Brinkman flow and transport of concentrated species in porous media. Electrolyte transport and charge conservation were solved using the Tertiary Current Distribution, Nernst–Planck formulation.

COMSOL's own meshing tool was used to generate the mesh within the geometry simulated. A mapped mesh was defined for the subdomain between the surface of the electrode and the reference electrode delimited by boundaries ⑥, ⑦, ⑭ and ⑮, where fixed numbers of elements, 4 and 400, were defined along boundaries ⑥/⑦ and ⑭/⑮, respectively. A triangular mesh calibrated for fluid dynamics was defined in the GDE domain with a maximum element growth size of 1.08 and curvature factor of 0.25. A triangular mesh calibrated for fluid dynamics was also defined for the GFC domain and the remaining regions of the EFC, with a maximum element growth rate of 1.2 and a curvature factor of 0.4. The total number of finite elements was 16874, with 15274 triangular elements, 1600 quadrilateral elements, 1705 edge elements and 16 vertex elements (Figure S2).

The model equations were solved in two coupled stages embedded in an outer iterative loop. First, a steady-state

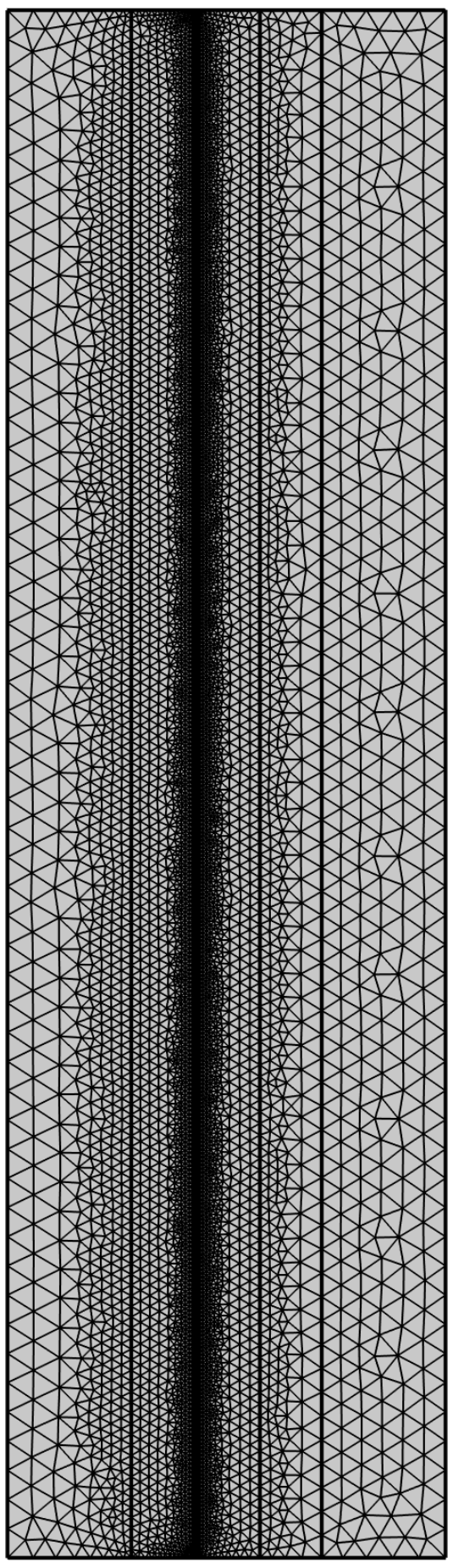

FIGURE S2 | Finite-element mesh used in the 2D geometry simulated in this study.

solution was obtained under open-circuit conditions to provide physically realistic initial values for flow and species fields. Second, the cathode potential was applied and electrochemical reactions were enabled. The model was then solved transiently for 30 s, which was sufficient for all partial current densities to reach quasi-steady values. Because gas density and viscosity depend on gas composition and influence both flow and transport, the flow and electrochemistry stages were repeated with updated gas properties. Three global iterations were sufficient for changes in the flow field and partial current densities to become negligible.

S2 Additional results

S2.1 Additional model validation and product-current trends

The main text focuses on the CO_2-to-CO, HER and CO-to-C_2H_4 pathways because these are the most relevant pathways for establishing the transition from kinetic control to transport limitation and intermediate-limited C_{2+} formation. Additional product-current trends are shown in Figure S3. The model captures the correct order of magnitude and qualitative trends for several CO-derived products, but the disagreement is most pronounced for C_2H_5OH. This deviation shows that CO availability, while necessary for C_{2+} formation, is not sufficient to determine ethanol-specific branching. Previous Cu-MEA simulations reproduced different ethylene and ethanol trends by assigning product-specific kinetic parameters, while recent mechanistic work indicates that ethylene and ethanol can proceed through distinct surface intermediates and Cu active sites [6, 40]. The C_2H_5OH mismatch therefore defines the mechanistic boundary of the present reduced kinetic network: the model is designed to resolve reactor-scale transport, CO_2 utilisation, CO intermediate availability and gas-phase hydrodynamic feedback, not to provide a complete microkinetic description of alcohol selectivity.

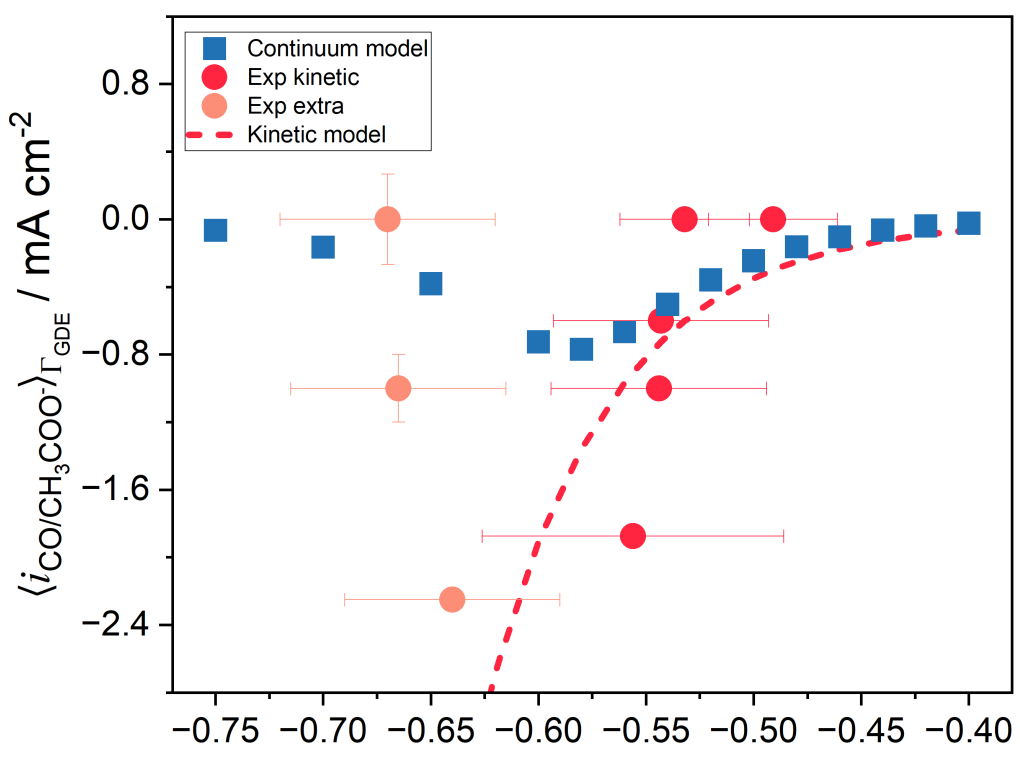

(a) CO-to-CH_3COO^-

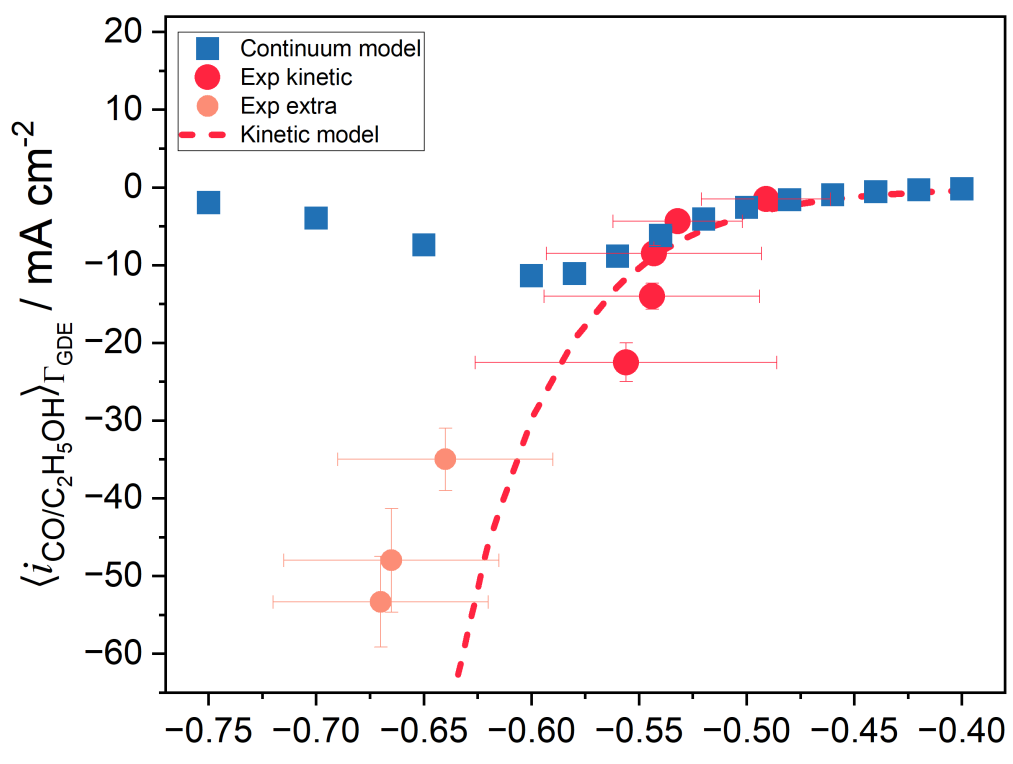

(b) CO-to-C_2H_5OH

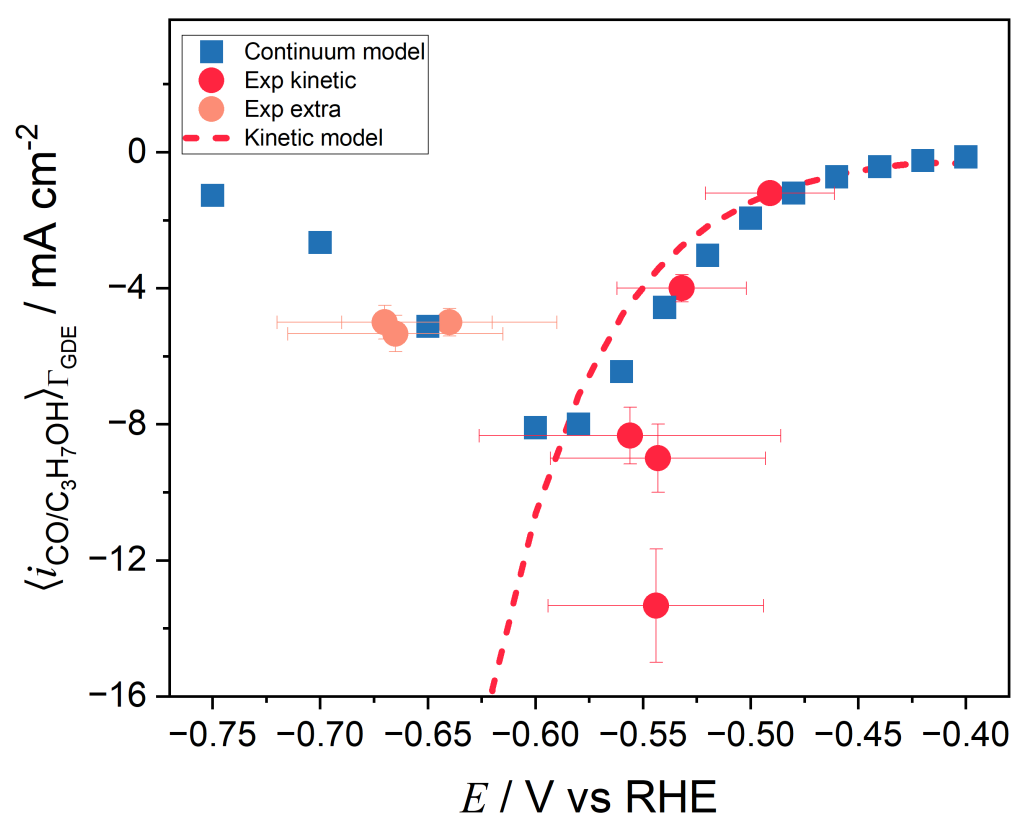

(c) CO-to-C_3H_7OH

FIGURE S3 | Additional experimental and simulated partial current densities for CO-derived products. The CO-to-C_2H_4 pathway is shown in the main text because it is the dominant C_{2+} product in the validation system. The remaining products are interpreted at the level of qualitative trend and order of magnitude, consistent with the reduced kinetic network used in the model.

These additional comparisons should therefore be interpreted as a test of whether the reduced model captures the correct direction and approximate magnitude of multi-product formation, not as a claim of complete

microkinetic predictability. The reactor-scale conclusions in the main text rely primarily on quantities that are directly constrained by conservation and transport: CO_2 utilisation, CO generation, CO consumption, gaseous product formation, composition-dependent gas-density changes and gas-velocity feedback.

S2.2 Additional gas-phase composition fields

Figure S4 shows the gas-phase distributions of CO and C_2H_4. The CO molar fraction does not increase monotonically with increasingly negative cathode potential. At intermediate potentials, CO accumulates because CO_2-to-CO is active while subsequent CO consumption remains comparatively limited. At more negative potentials, CO consumption toward C_{2+} products increases and CO is depleted more strongly. By contrast, C_2H_4 increases over the potential range in which CO availability and CO-to-C_2H_4 kinetics are favourably balanced, before the growth of HER becomes increasingly important at the most negative potentials.

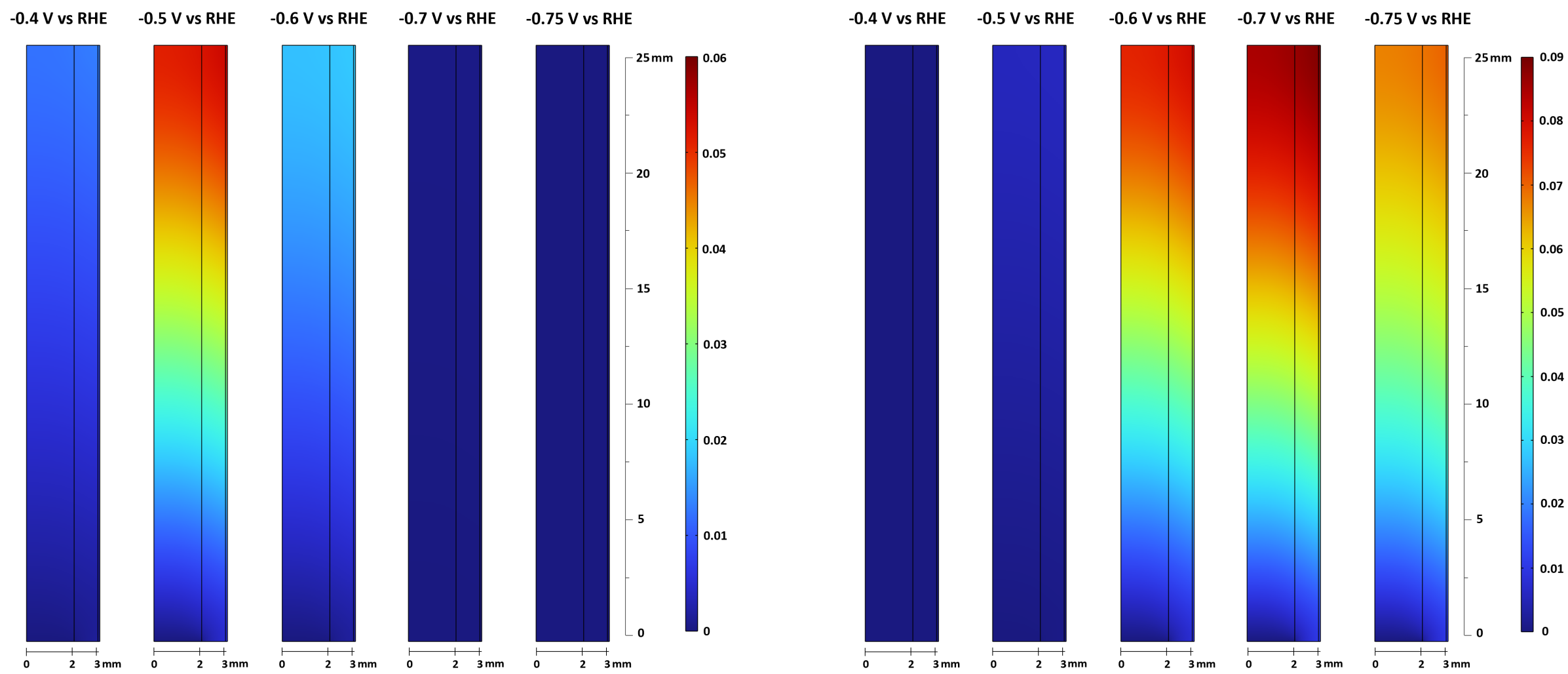

(a) x_{CO} in GFC and GDE **(b)** $x_{C_2H_4}$ in GFC and GDE

FIGURE S4 | Gas-phase molar fractions of CO and C_2H_4 at various applied cathode potentials.

S2.3 Through-plane gas-phase profiles across the GDL

To quantify the gas-phase gradients across the porous electrode, through-plane partial-pressure profiles were evaluated across the full 80 μm GDL thickness at electrode positions y = 0.5, 2, 8, 16 and 24 mm. Profiles were obtained at cathode potentials of −0.4, −0.5, −0.6, −0.7 and −0.75 V vs RHE. The selected positions span the region from close to the gas inlet to close to the outlet, enabling the direct comparison of through-plane gradients with the longitudinal evolution of the reacting gas phase.

Figures S5 and S6 show the corresponding CO_2 and H_2 partial-pressure profiles. At each position along the electrode, the variation across the GDL thickness is comparatively small. The CO_2 partial pressure decreases slightly from the gas-channel side towards the catalyst-layer boundary, consistent with consumption and transfer at the reactive interface. The opposite trend is observed for H_2, whose partial pressure increases towards the catalyst-layer boundary as H_2 is generated electrochemically at the interface.

The larger variation occurs along the electrode length rather than across the GDL thickness. As the cathode potential becomes more negative, the absolute CO_2 partial pressure decreases progressively with increasing y, while the H_2 partial pressure increases. This longitudinal redistribution becomes increasingly pronounced at the most negative potentials. The profiles therefore confirm that, under the conditions considered here, the dominant gas-phase composition gradients arise along the flow direction, while comparatively weak gradients develop across the 80 μm GDL.

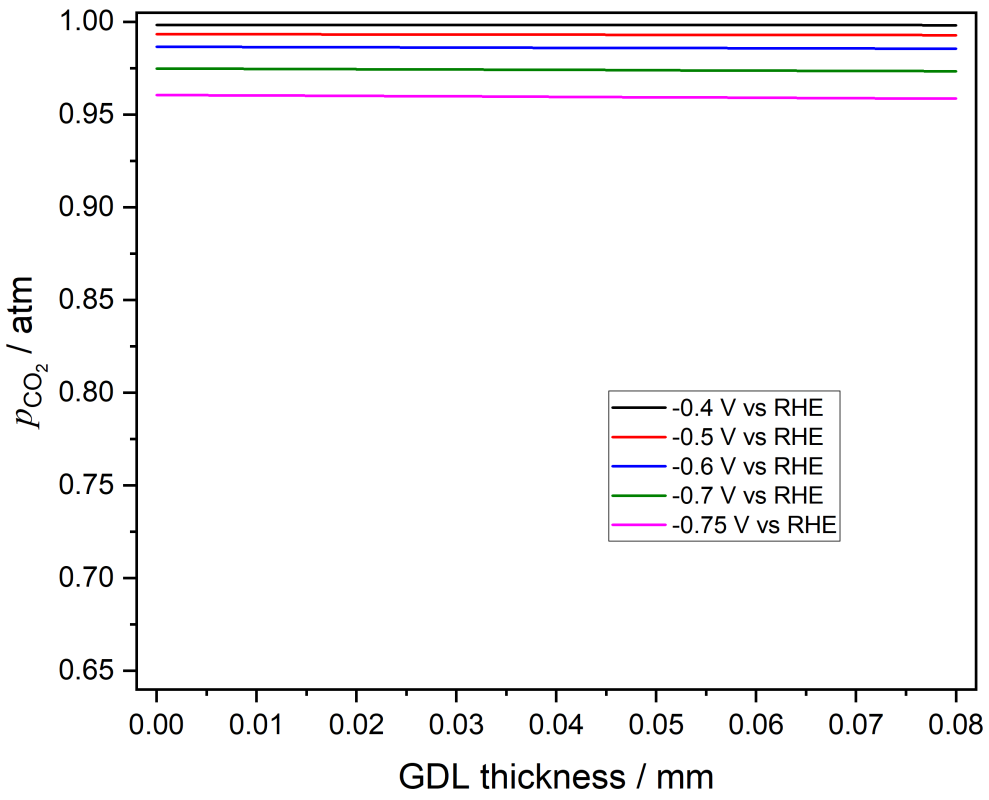

(**a**) y = 0.5 mm

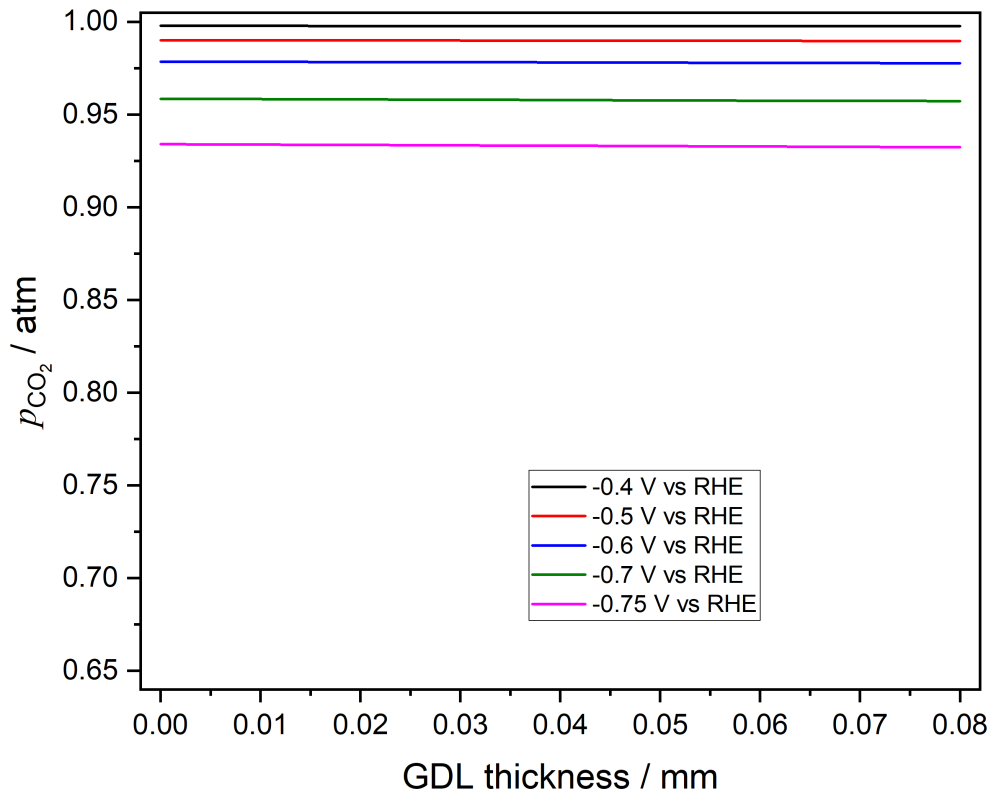

(**b**) y = 2 mm

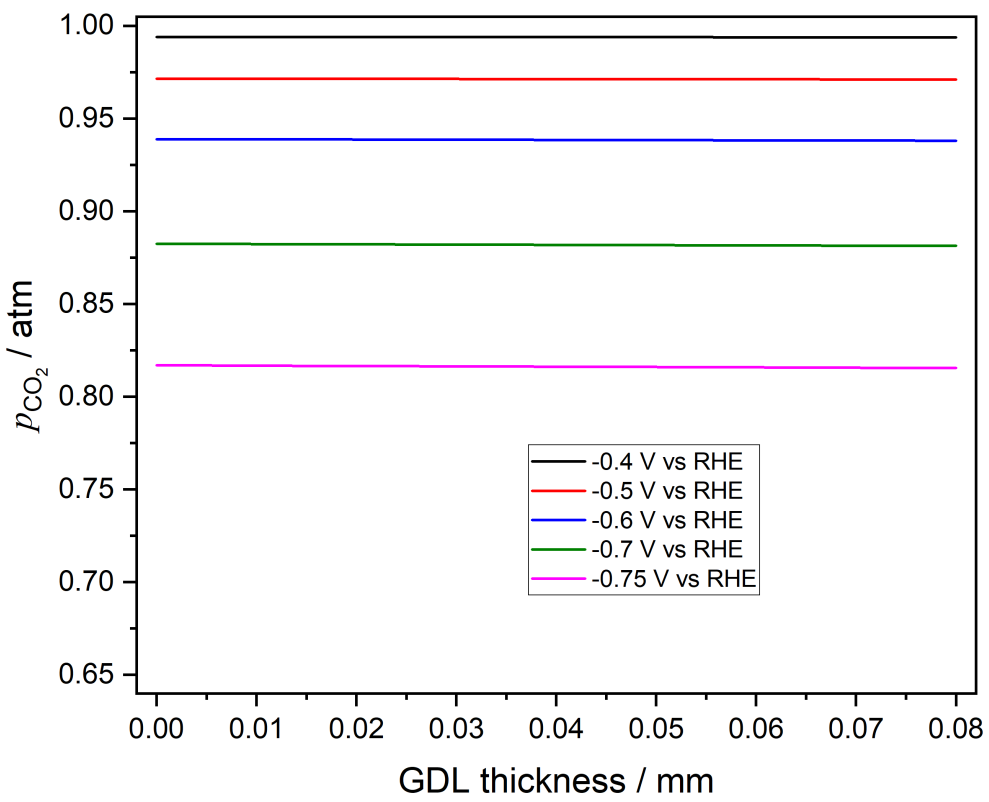

(**c**) y = 8 mm

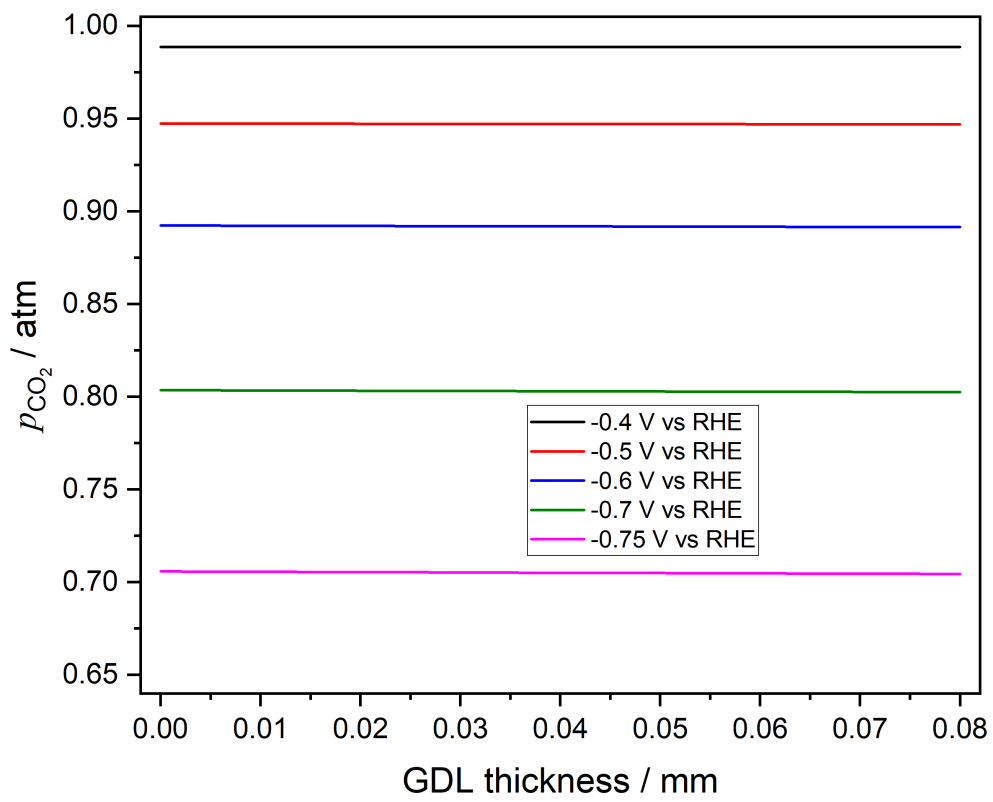

(**d**) y = 16 mm

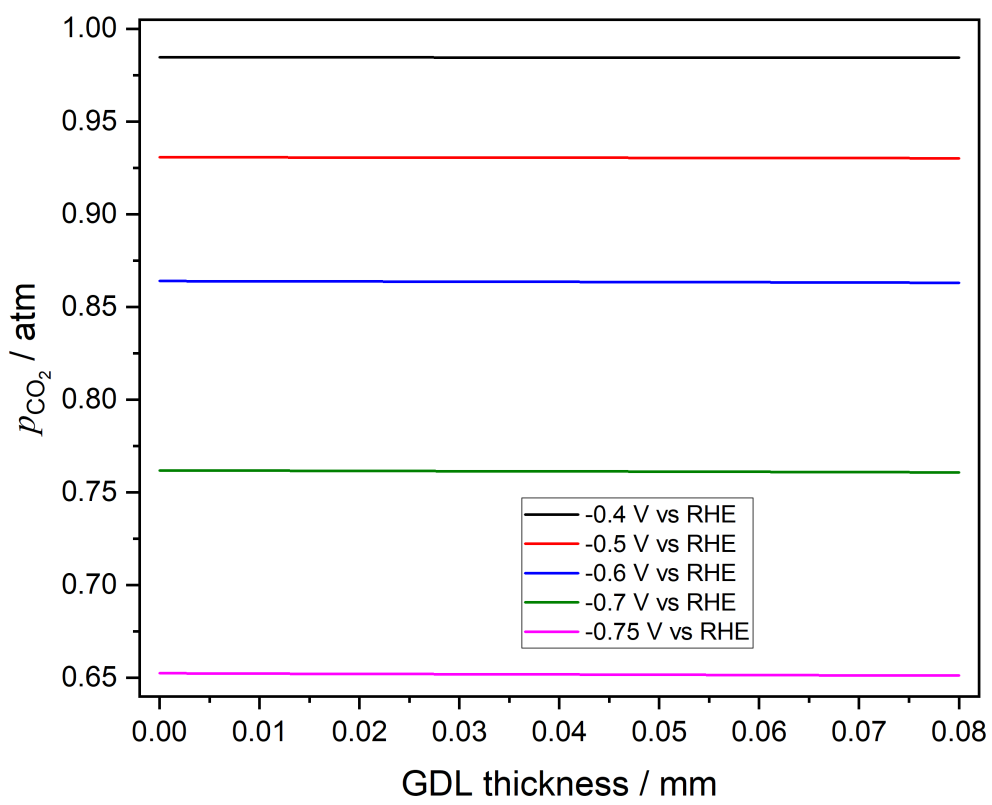

(**e**) y = 24 mm

FIGURE S5 | Through-plane CO_2 partial-pressure profiles across the 80 μm GDL at different positions along the electrode. Profiles are shown at y = 0.5, 2, 8, 16 and 24 mm for cathode potentials from −0.4 to −0.75 V vs RHE. The CO_2 partial pressure decreases slightly towards the catalyst-layer boundary across the GDL thickness (right), while substantially larger changes develop with position along the electrode and applied cathode potential.

S2.4 Hydrogen evolution and local supersaturation

Figure S7 shows the spatial distribution of HER current density, H_2 partial pressure and dissolved H_2 concentration at the GDE surface. The HER current density is comparatively uniform along the electrode

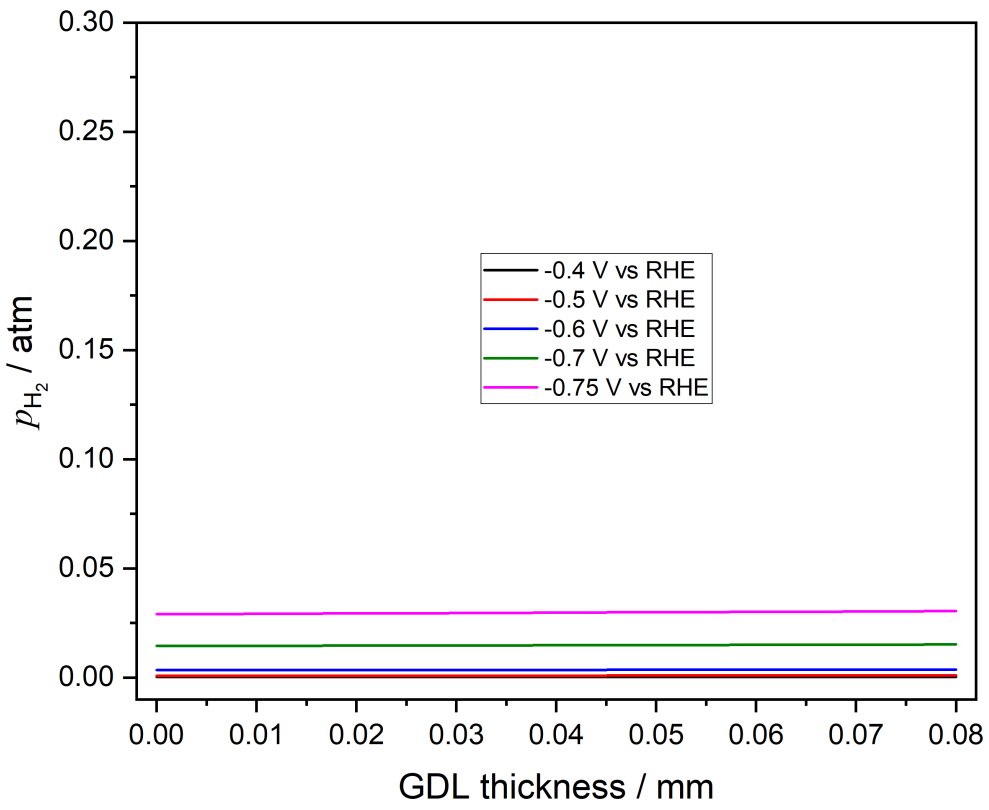

(a) y = 0.5 mm

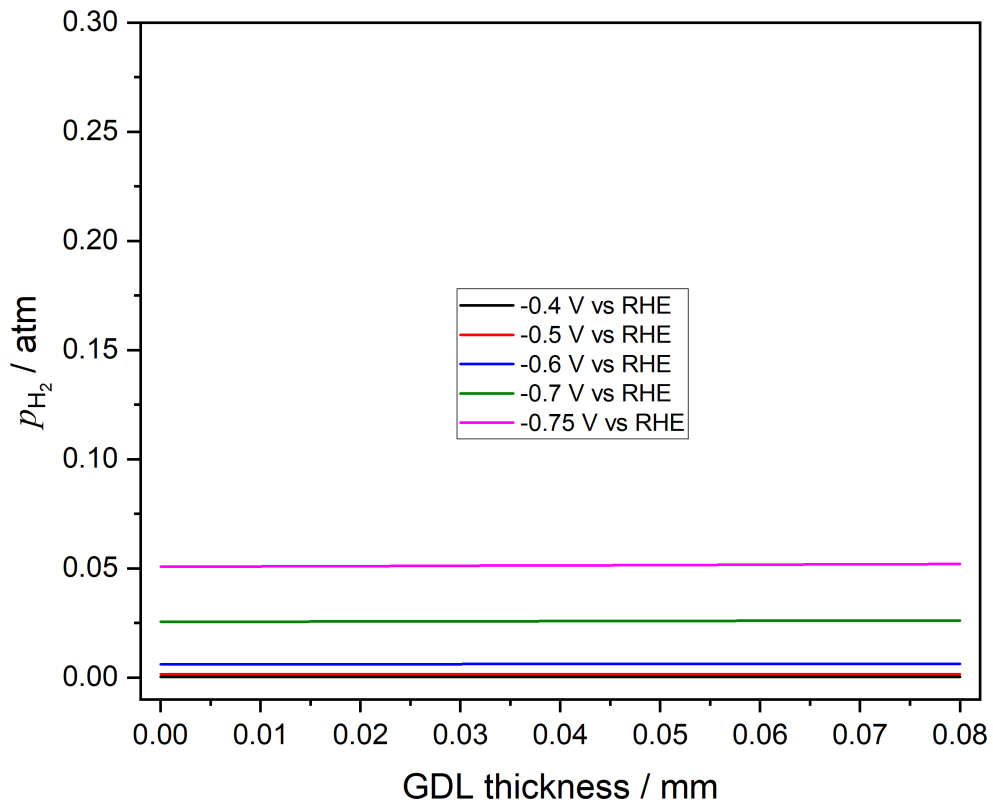

(b) y = 2 mm

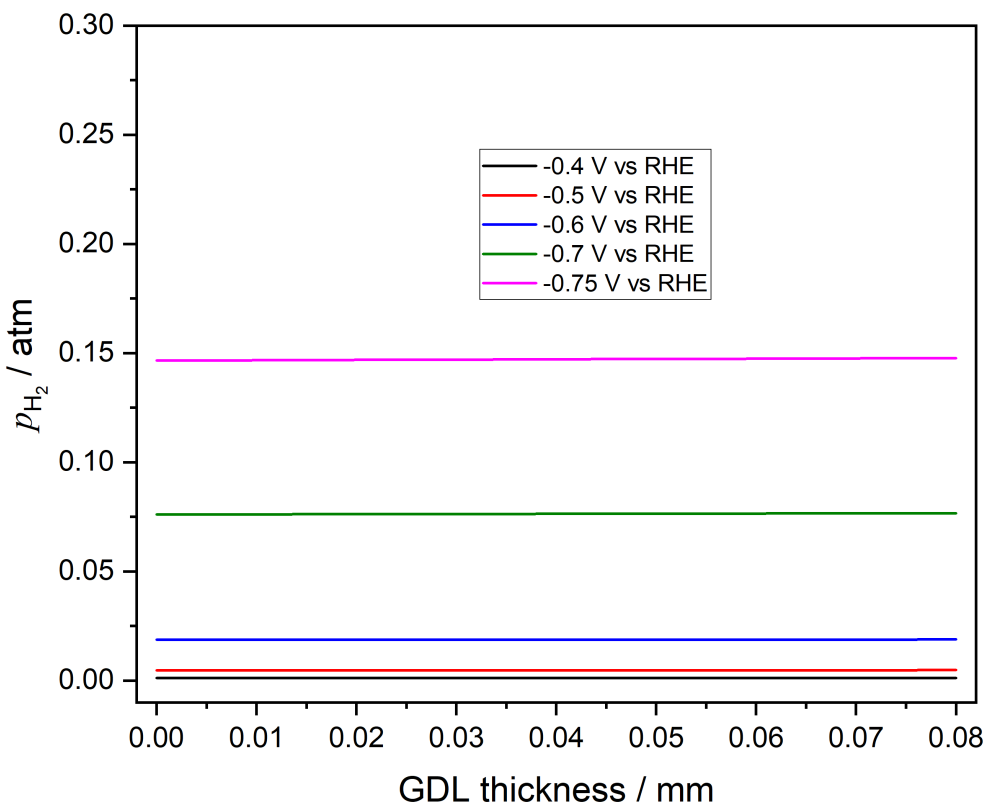

(c) y = 8 mm

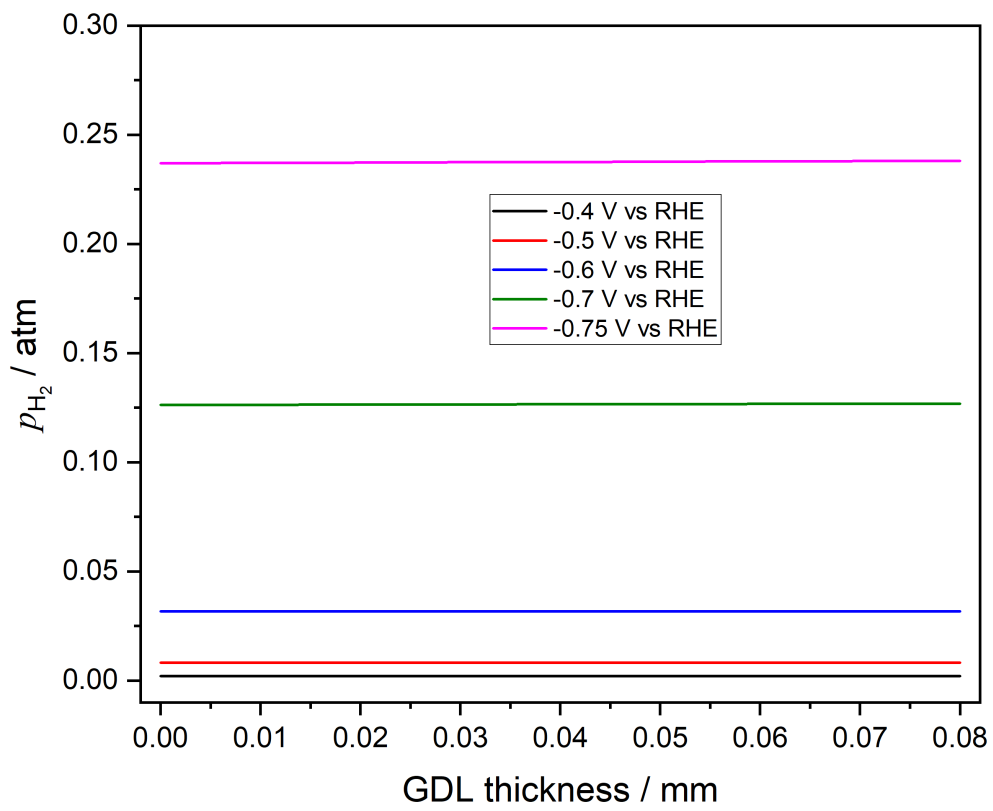

(d) y = 16 mm

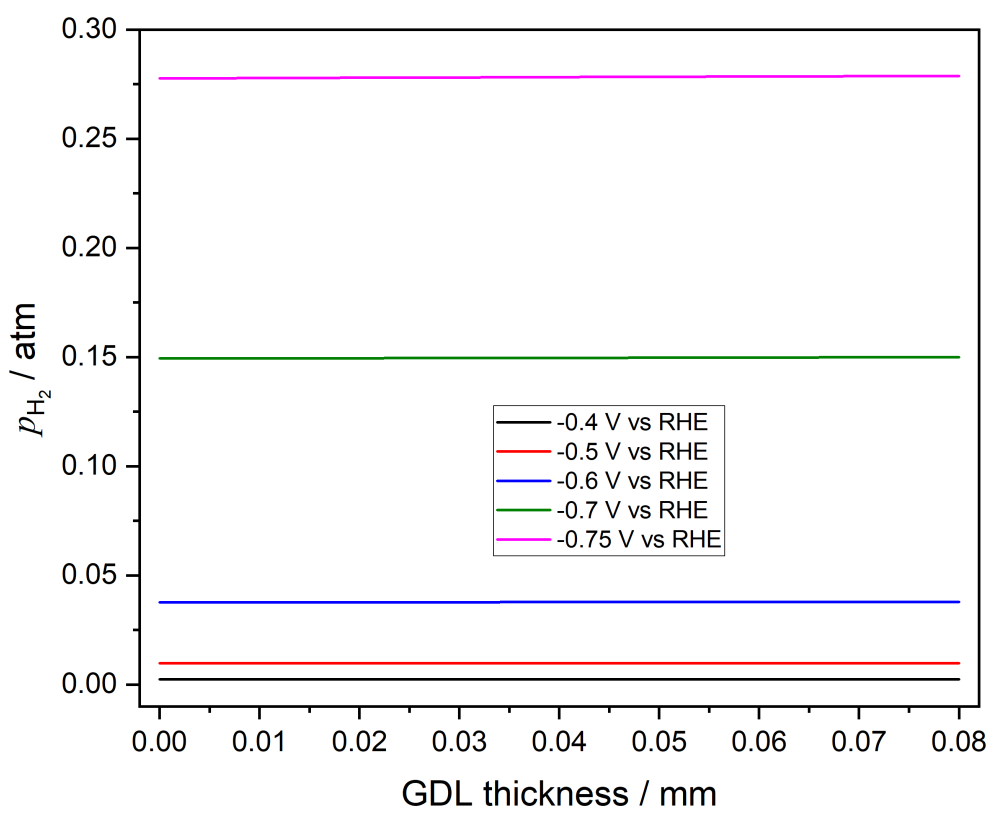

(e) y = 24 mm

FIGURE S6 | Through-plane H_2 partial-pressure profiles across the 80 μm GDL at different positions along the electrode. Profiles are shown at y = 0.5, 2, 8, 16 and 24 mm for cathode potentials from −0.40 to −0.75 V vs RHE. The H_2 partial pressure increases slightly towards the catalyst-layer boundary (right), consistent with electrochemical generation at the reactive interface, while its absolute value increases much more strongly along the electrode length and at increasingly negative cathode potentials.

length at a given potential, reflecting the absence of a gaseous-reactant supply constraint analogous to that affecting the CO_2-to-CO pathway. The predicted dissolved H_2 concentration near the electrode becomes high

at the most negative potentials, indicating local supersaturation within the single-phase electrolyte description. In practice, such conditions would favour nucleation and growth of discrete gas bubbles. These subsequent two-phase processes are not explicitly resolved in the present model, but could further affect local liquid transport, wetting and electrochemically active area under HER-dominated conditions.

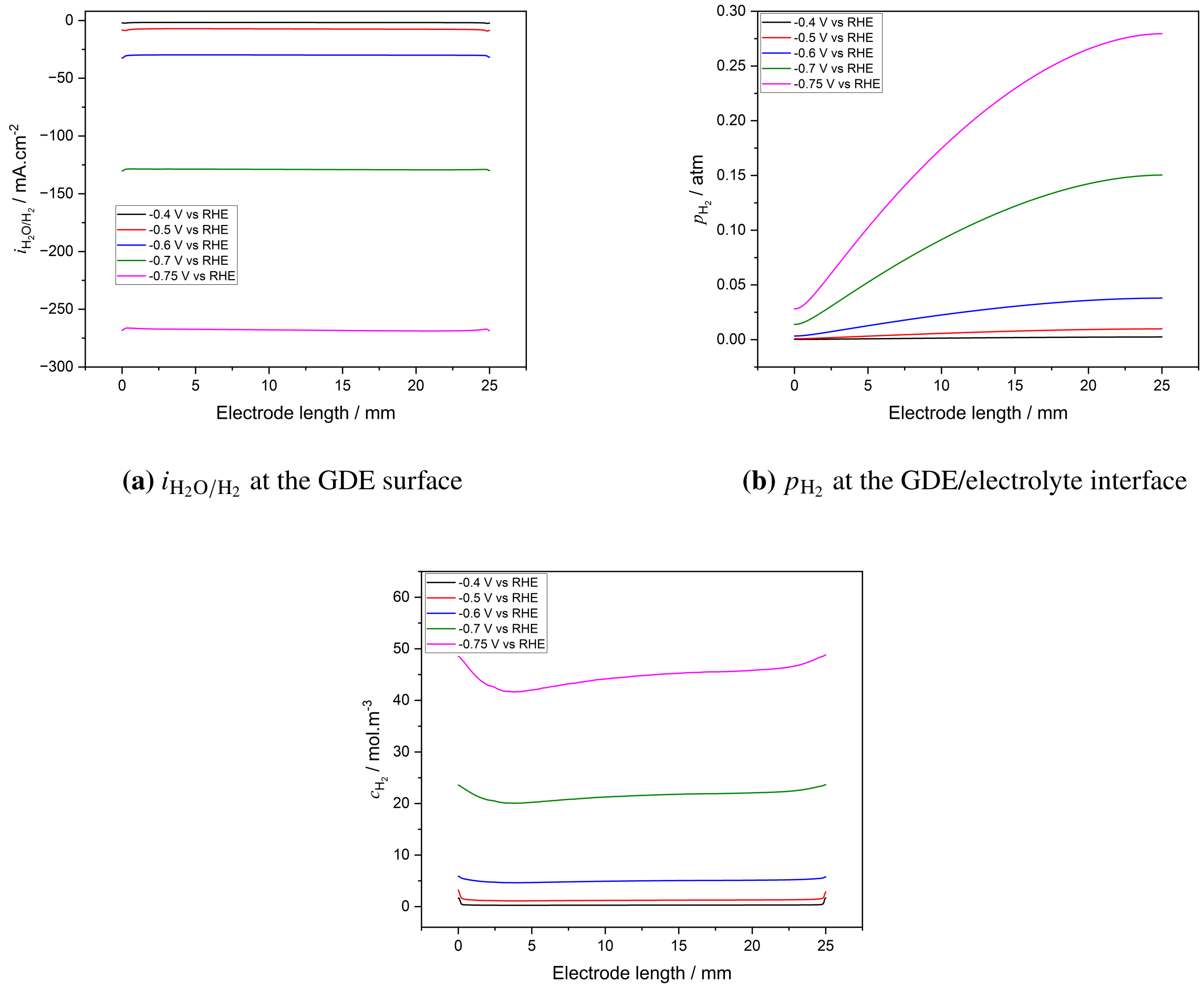

(a) i_{H_2O/H_2} at the GDE surface

(b) p_{H_2} at the GDE/electrolyte interface

(c) c_{H_2} at the GDE surface

FIGURE S7 | Spatial distribution of HER current density and corresponding H_2 partial pressure and dissolved concentration.

S2.5 Additional CO-to-product spatial distributions

Figure S8 shows the spatial distributions of the CO-to-CH_3COO^-, CO-to-C_2H_5OH and CO-to-C_3H_7OH partial current densities. These pathways follow the same general intermediate-availability constraint discussed in the main text, although their magnitudes and potential dependences differ because of the fitted kinetic parameters. The larger deviation observed for alcohols reinforces the limitation of describing all CO-derived products through lumped overall reactions.

S2.6 Through-plane electrolyte composition profiles

The two-dimensional electrolyte pH, OH^- and carbon-speciation fields are shown in Figure 6 of the main manuscript. To quantify the through-plane gradients underlying these fields, local profiles were evaluated from the GDE/electrolyte interface into the first 2 mm of the electrolyte channel at electrode positions $y = 0.5$, 2, 8, 16 and 24 mm. Profiles were obtained at cathode potentials of −0.40, −0.50, −0.60, −0.70 and −0.75 V

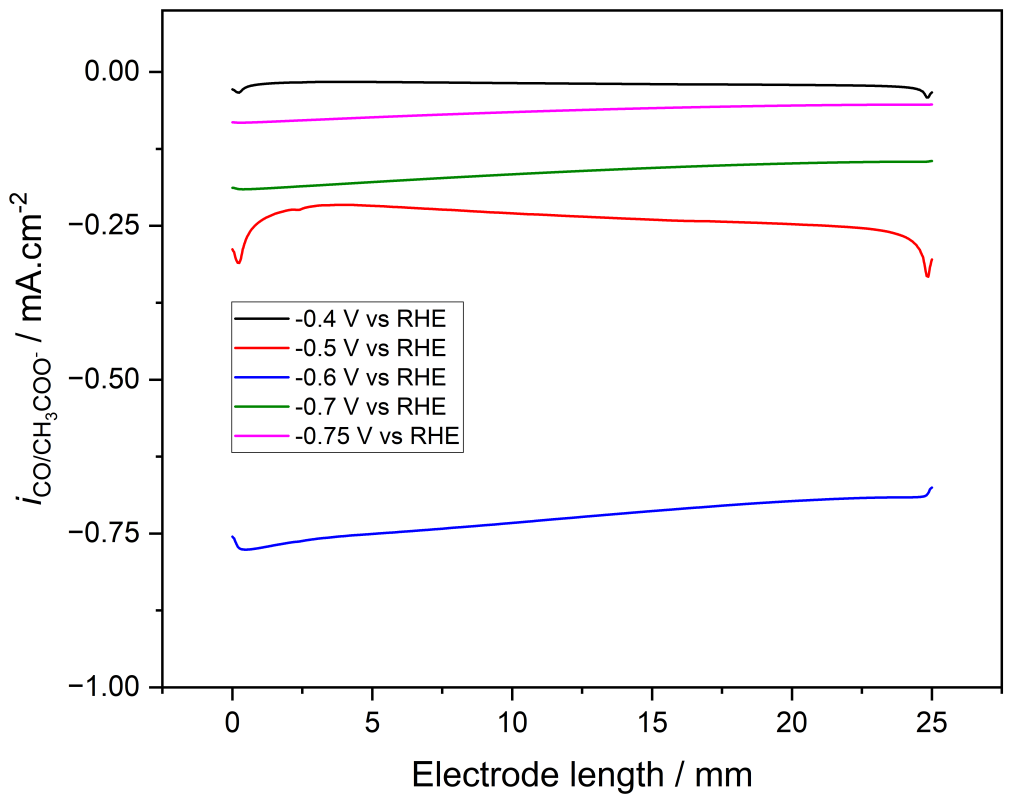

(a) i_{CO/CH_3COO^-}

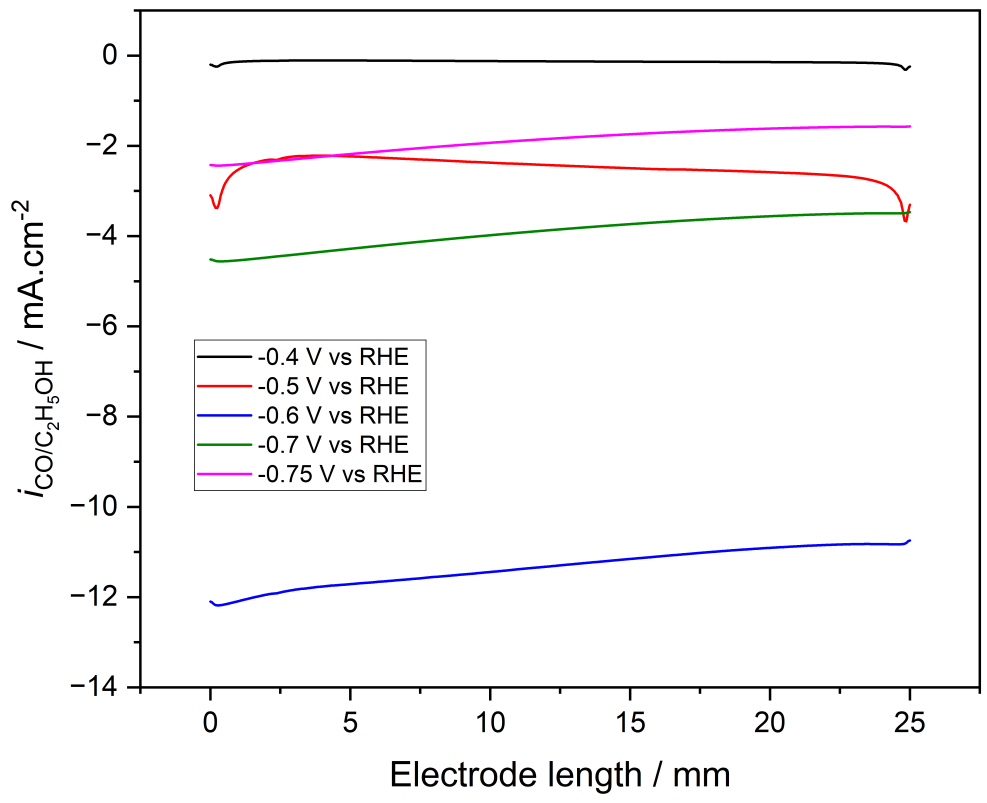

(b) i_{CO/C_2H_5OH}

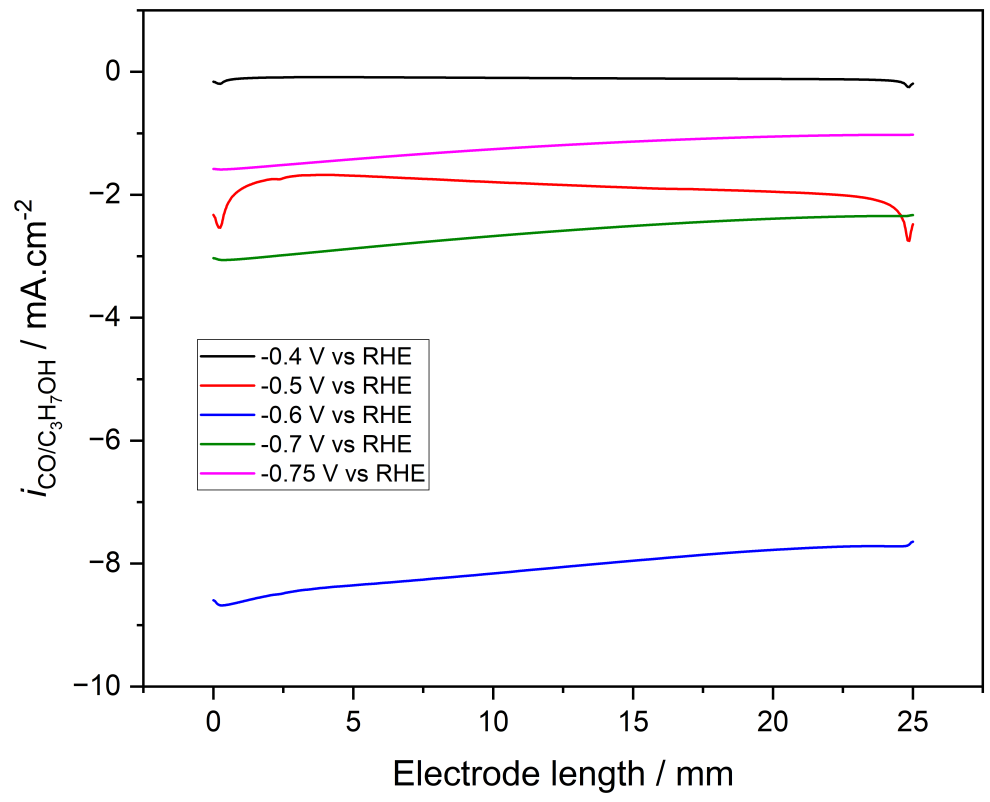

(c) i_{CO/C_3H_7OH}

FIGURE S8 | Spatial distribution of additional CO-to-product partial current densities at the GDE surface. The CO-to-C_2H_4 pathway and CO availability diagnostics are shown in the main text.

vs RHE.

Figures S9–S11 show the corresponding pH, HCO_3^- and CO_3^{2-} profiles. At mild cathodic potentials, a local decrease in pH develops close to the GDE surface, accompanied by substantial bicarbonate and carbonate concentrations in the near-electrode region. This behaviour reflects the consumption of OH^- through homogeneous reactions of absorbed CO_2. As the cathode potential becomes more negative, the pH gradient becomes progressively less pronounced and the near-surface bicarbonate and carbonate concentrations decrease markedly. This is consistent with the increasing fraction of interfacially transferred CO_2 being consumed electrochemically, together with increasing cathodic OH^- generation.

The strongest composition changes occur close to the GDE/electrolyte interface, but their magnitude and penetration into the electrolyte vary with position along the electrode. A one-dimensional through-plane description can therefore represent the local electrolyte boundary layer at a specified longitudinal position, whereas the two-dimensional model additionally captures how this local profile changes along the flow direction as CO_2 delivery and electrochemical conversion evolve.

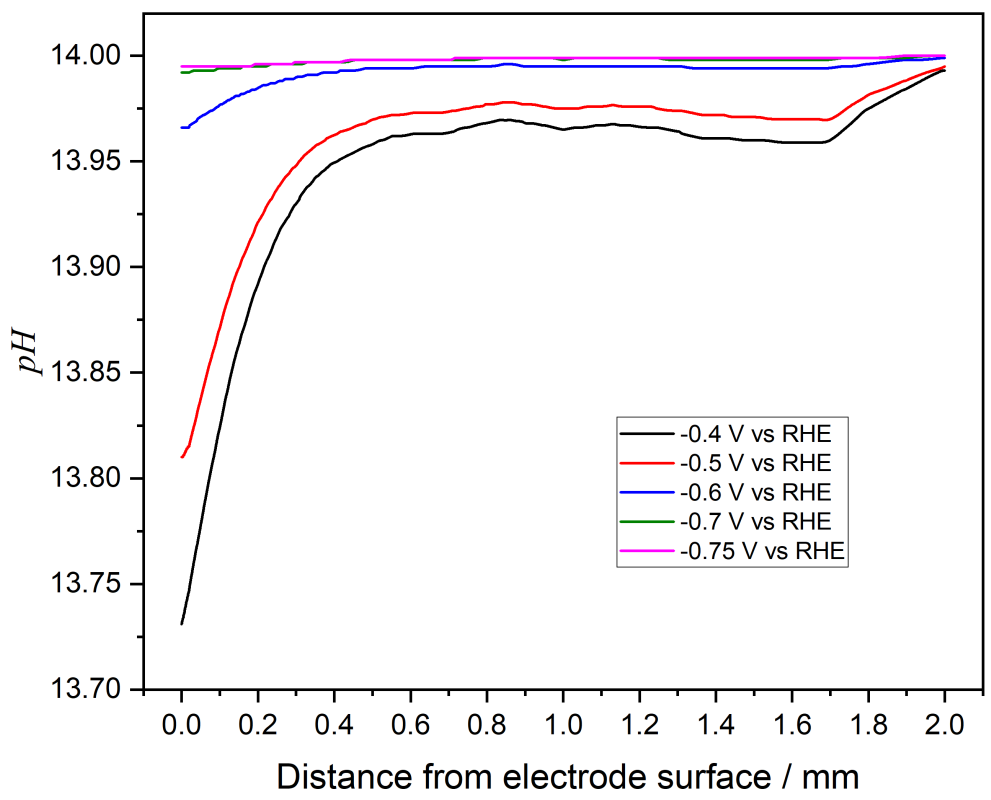

(a) y = 0.5 mm

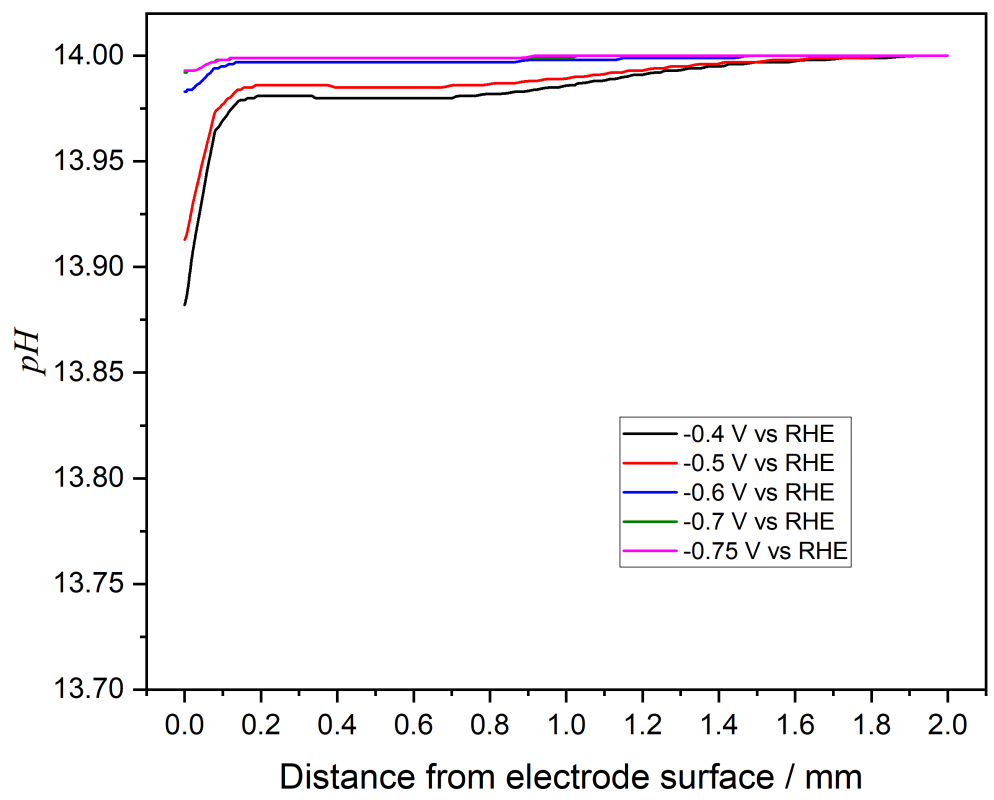

(b) y = 2 mm

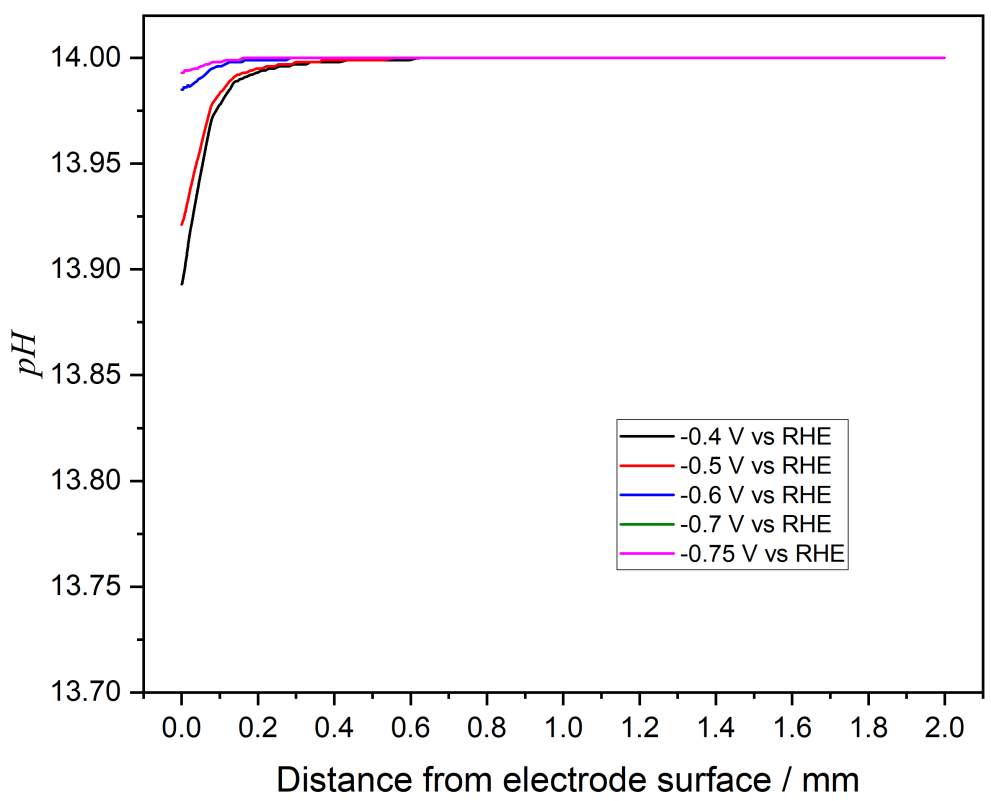

(c) y = 8 mm

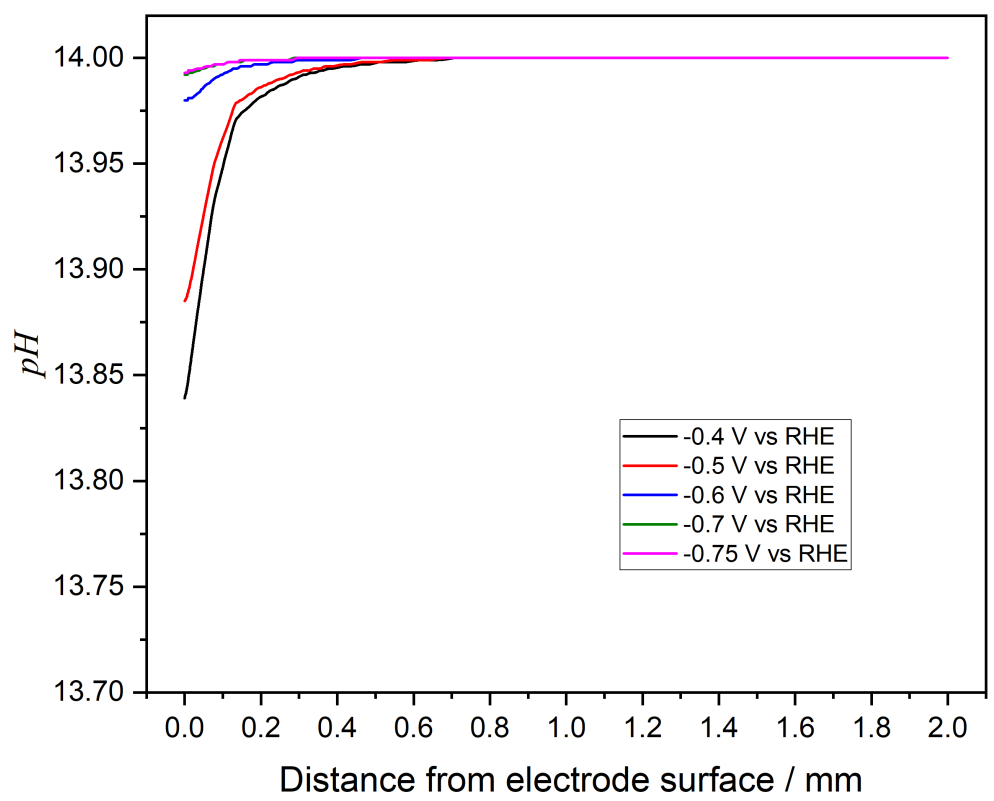

(d) y = 16 mm

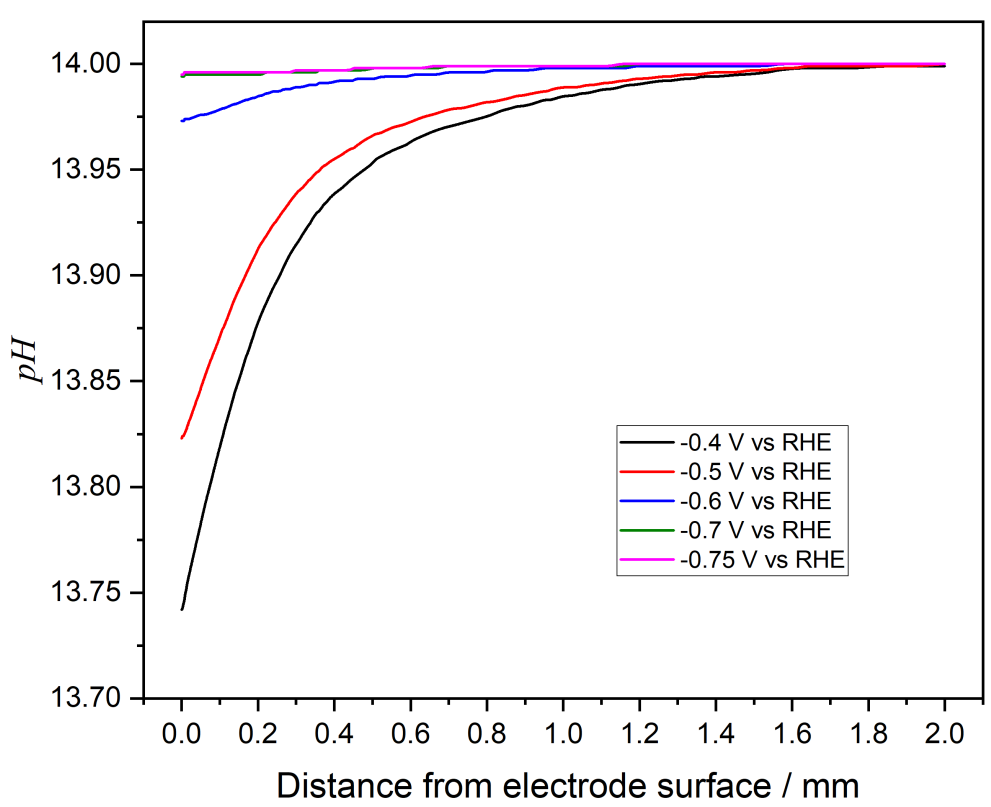

(e) y = 24 mm

FIGURE S9 | Through-plane pH profiles from the GDE/electrolyte interface into the first 2 mm of the electrolyte channel at different positions along the electrode. Profiles are shown at y = 0.5, 2, 8, 16 and 24 mm for cathode potentials from −0.40 to −0.75 V vs RHE. The local pH decrease is most pronounced at mild cathodic potentials and becomes progressively weaker as the cathode potential becomes more negative, while the bulk electrolyte remains strongly alkaline.

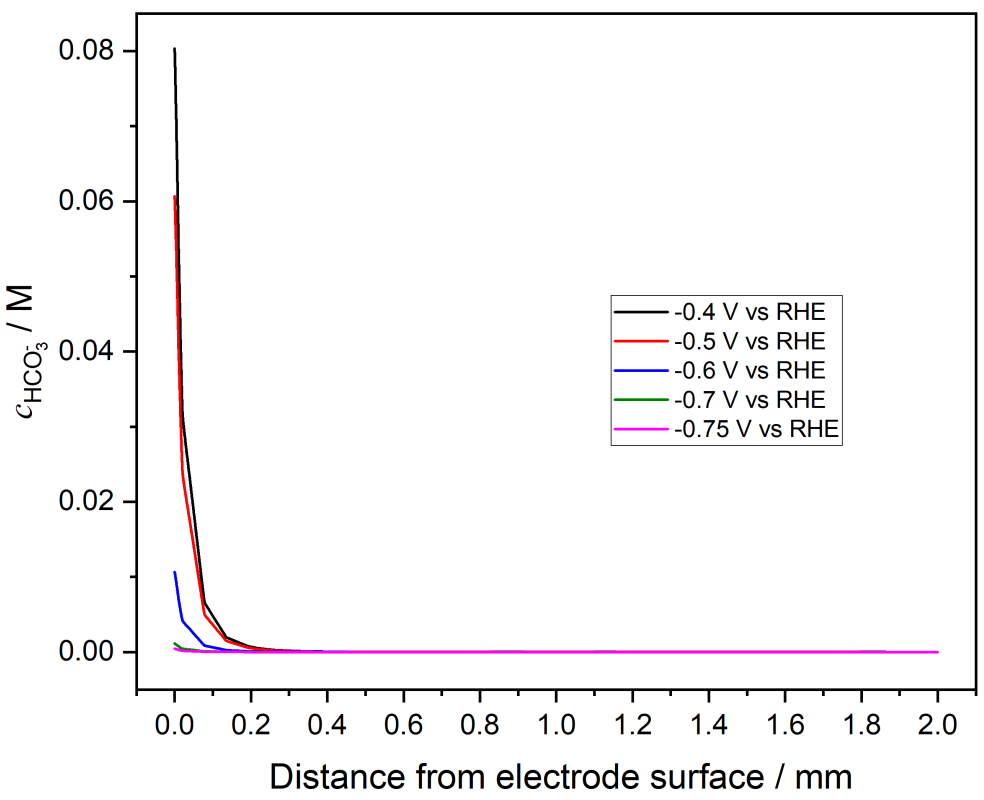

(a) y = 0.5 mm

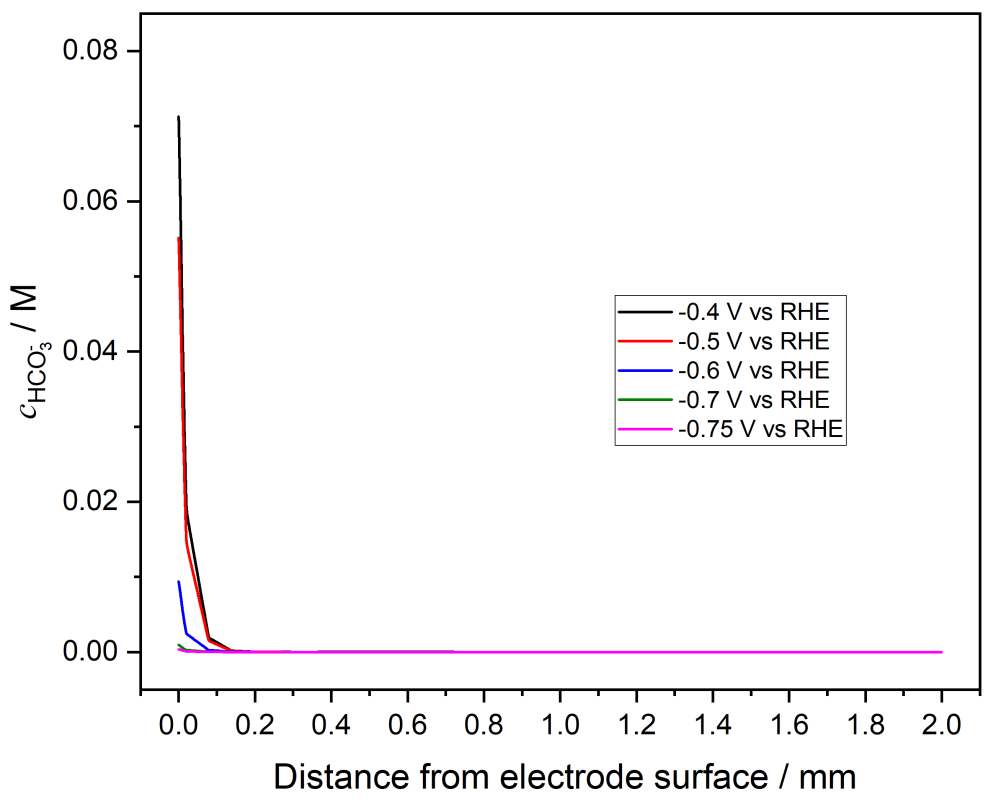

(b) y = 2 mm

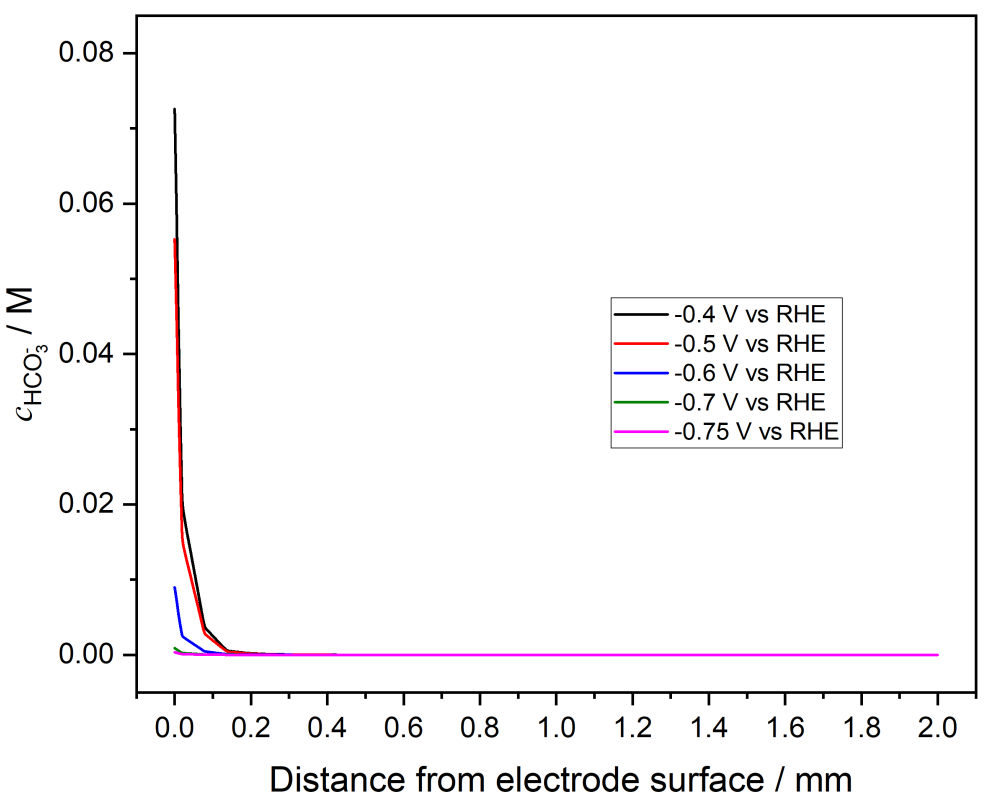

(c) y = 8 mm

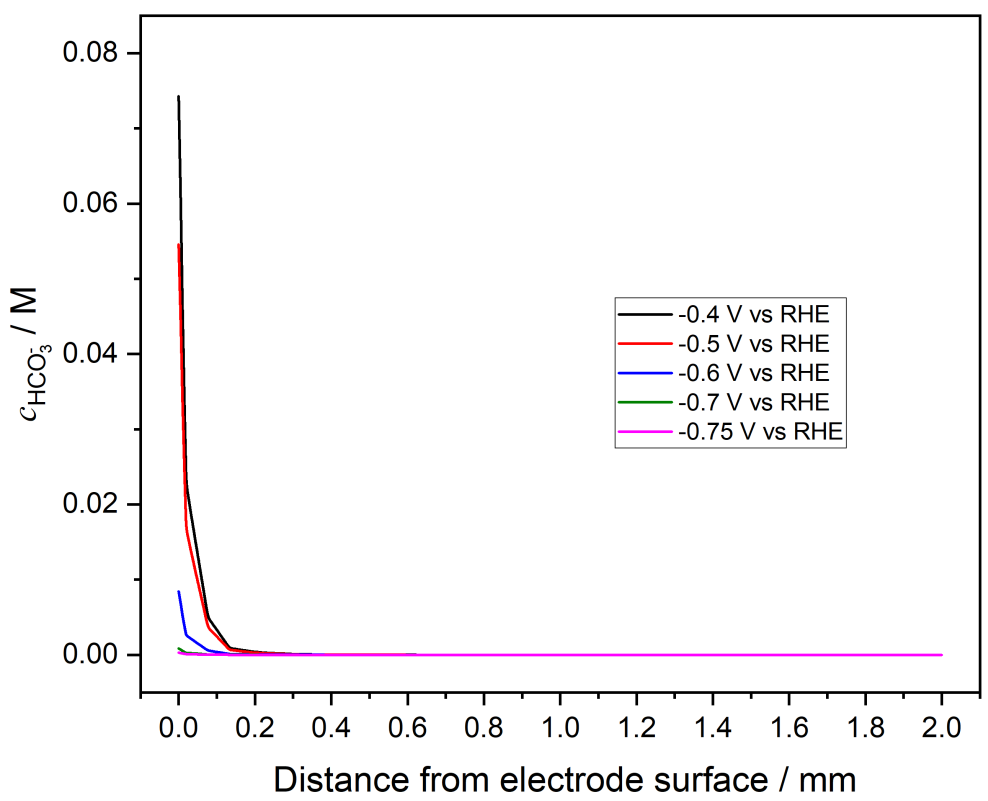

(d) y = 16 mm

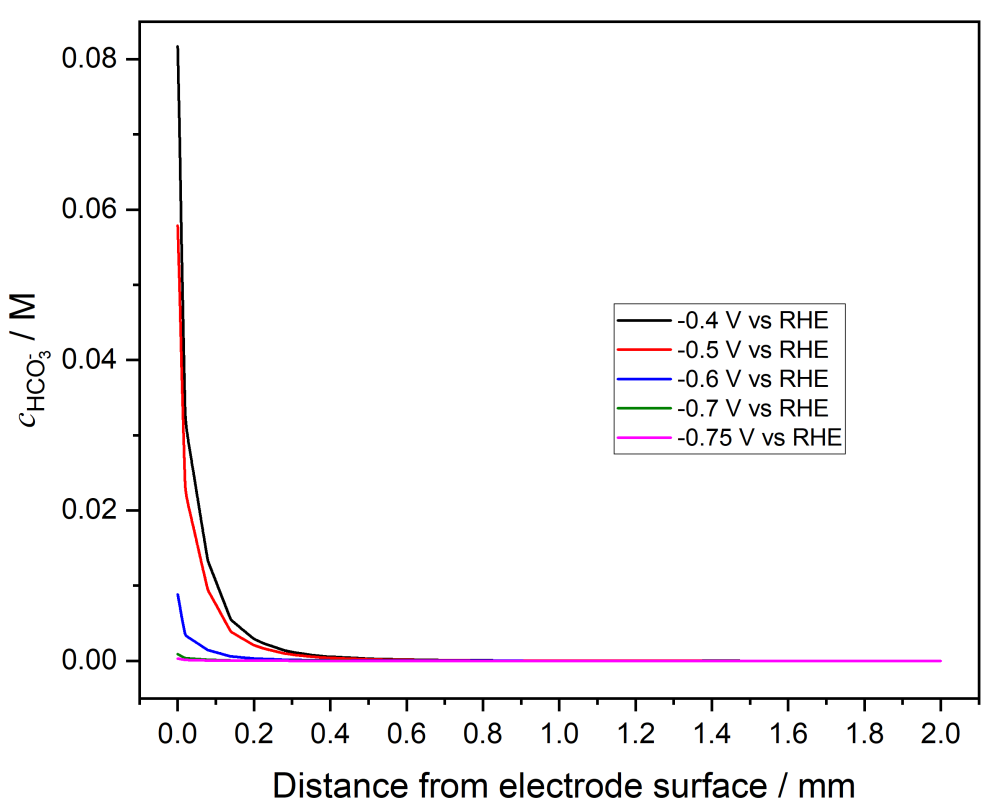

(e) y = 24 mm

FIGURE S10 | Through-plane HCO_3^- concentration profiles from the GDE/electrolyte interface into the first 2 mm of the electrolyte channel at different positions along the electrode. Profiles are shown at y = 0.5, 2, 8, 16 and 24 mm for cathode potentials from −0.40 to −0.75 V vs RHE. Bicarbonate accumulates close to the GDE surface at mild cathodic potentials and decays towards the bulk electrolyte, while the near-surface concentration decreases strongly as the cathode potential becomes more negative.

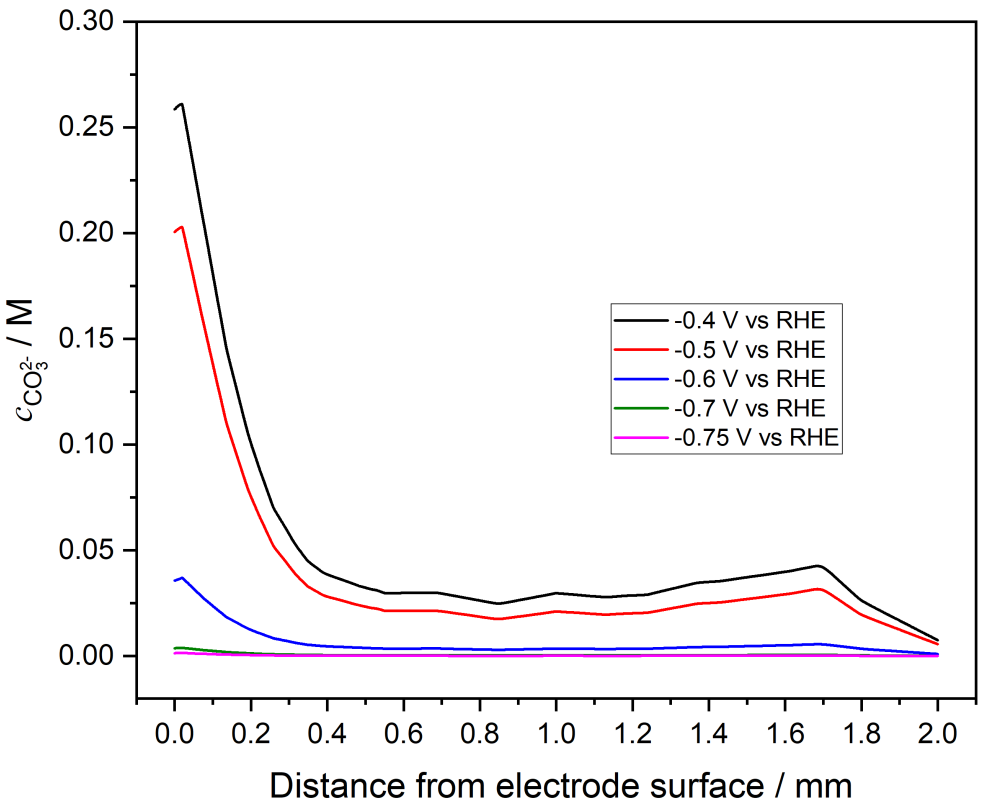

(a) y = 0.5 mm

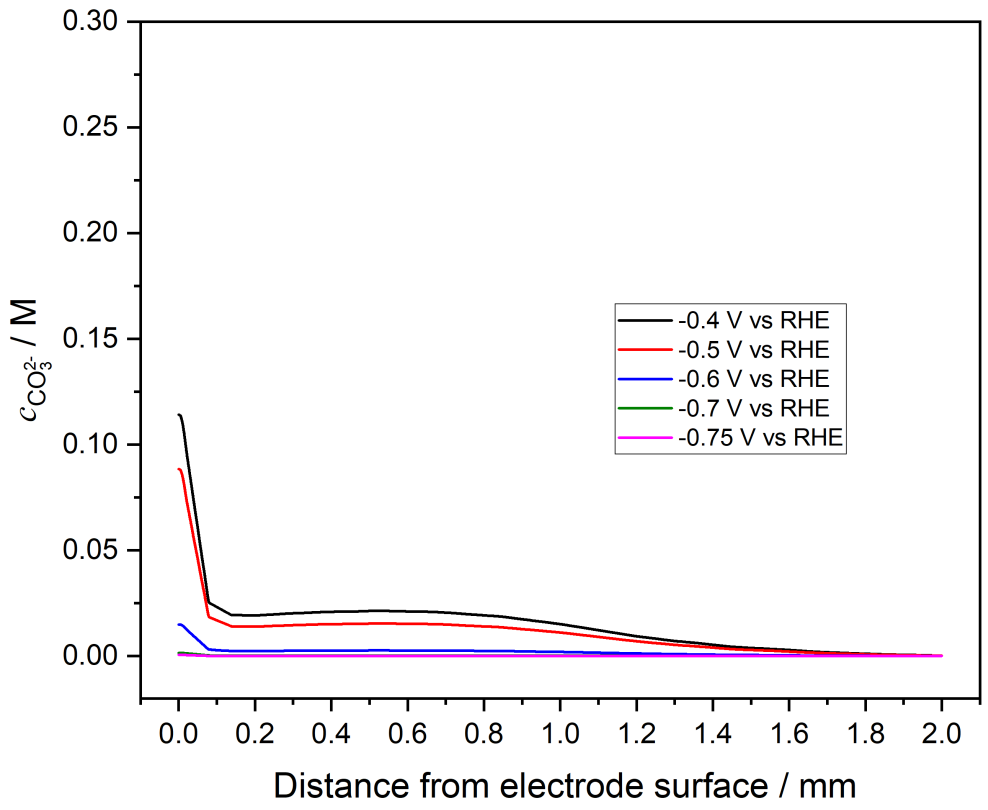

(b) y = 2 mm

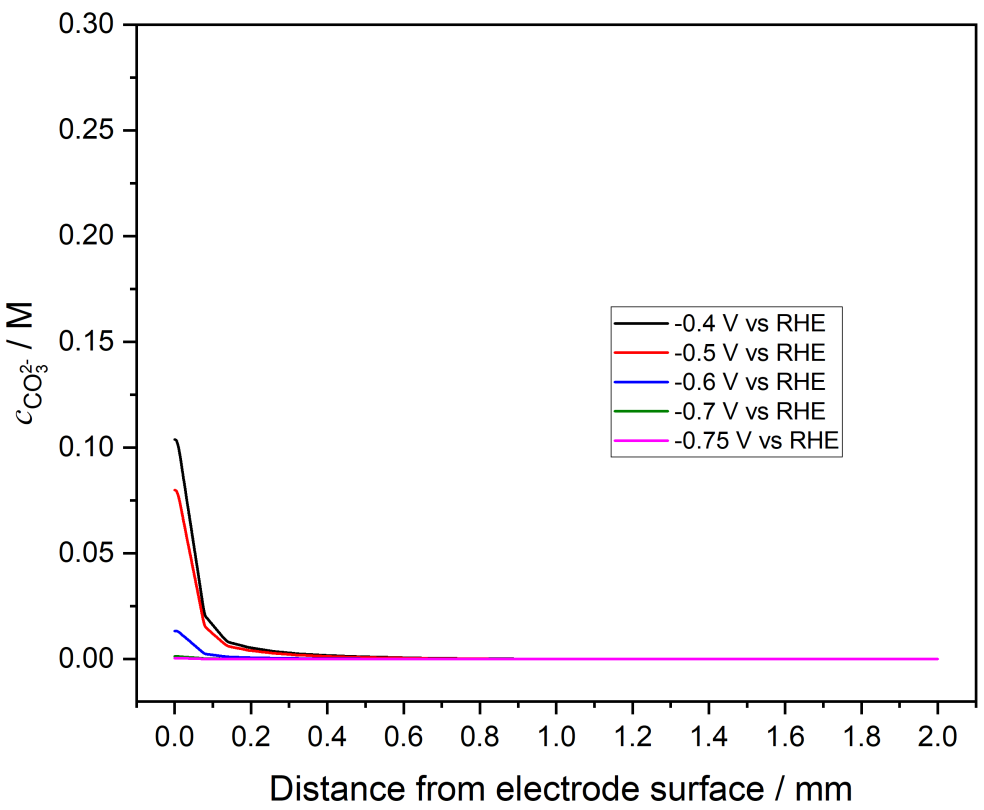

(c) y = 8 mm

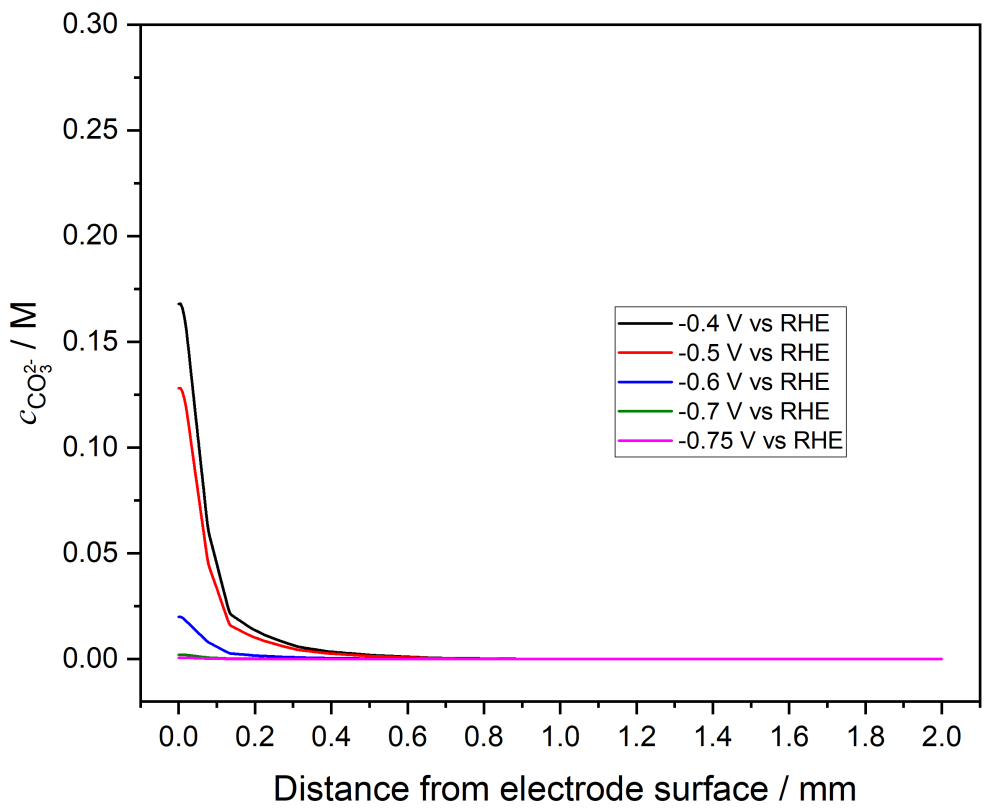

(d) y = 16 mm

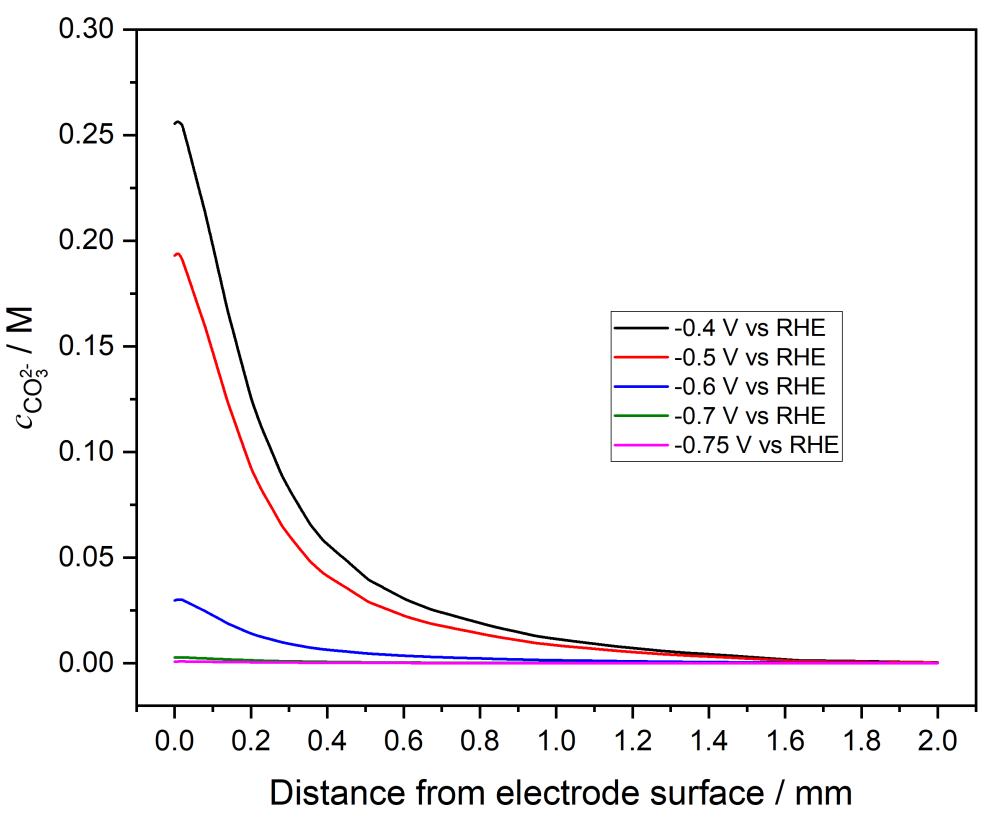

(e) y = 24 mm

FIGURE S11 | Through-plane CO_3^{2-} concentration profiles from the GDE/electrolyte interface into the first 2 mm of the electrolyte channel at different positions along the electrode. Profiles are shown at y = 0.5, 2, 8, 16 and 24 mm for cathode potentials from −0.40 to −0.75 V vs RHE. Carbonate concentrations are highest close to the GDE surface at mild cathodic potentials and generally decrease towards the bulk electrolyte, while substantially lower concentrations are predicted at increasingly negative cathode potentials.

References

[1] Venkata S.R.K. Tandava et al. Modulating the surface interface of PTFE/Cu-based GDEs to boost the electrochemical conversion of CO_2 to C_2H_4 at ultra-low overpotential. *Applied Catalysis B: Environment and Energy*, 371:125276, 2025. doi: 10.1016/j.apcatb.2025.125276.

[2] Ángel Frías-Ferrer et al. Optimized design of an electrochemical filter-press reactor using CFD methods. *Chemical Engineering Journal*, 169(1):270–281, 2011. doi: 10.1016/j.cej.2011.02.053.

[3] Recep Kas et al. Along the Channel Gradients Impact on the Spatioactivity of Gas Diffusion Electrodes at High Conversions during CO_2 Electroreduction. *ACS Sustainable Chemistry & Engineering*, 9:1286–1296, 2021. doi: 10.1021/acssuschemeng.0c07694.

[4] Lien-Chun Weng, Alexis T. Bell, and Adam Z. Weber. Towards membrane-electrode assembly systems for CO_2 reduction: a modeling study. *Energy & Environmental Science*, 12:1950–1968, 2019. doi: 10.1039/C9EE00909D.

[5] Eric W. Lees et al. Exploring CO_2 reduction and crossover in membrane electrode assemblies. *Nature Chemical Engineering*, 1:340–353, 2024. doi: 10.1038/s44286-024-00062-0.

[6] Lien-Chun Weng, Alexis T. Bell, and Adam Z. Weber. A systematic analysis of Cu-based membrane-electrode assemblies for CO2 reduction through multiphysics simulation. *Energy & Environmental Science*, 13:3592–3606, 2020. doi: 10.1039/D0EE01604G.

[7] Hugo-Pieter Iglesias van Montfort and Thomas Burdyny. Mapping Spatial and Temporal Electrochemical Activity of Water and CO2 Electrolysis on Gas-Diffusion Electrodes Using Infrared Thermography. *ACS Energy Letters*, 7(8):2410–2419, 2022. doi: 10.1021/acsenergylett.2c00984.

[8] Kyle R. Disselkoen et al. Continuum Model for Optimizing CO Reduction Gas Diffusion Electrodes. *ACS Sustainable Chemistry & Engineering*, 12(19):7235–7245, 2024. doi: 10.1021/acssuschemeng.3c05194.

[9] Zahra Teimouri et al. Integrating atomic scale catalyst design with transport engineering for stable and efficient CO_2 electrolysis to CO in a membrane electrode assembly. *Energy & Environmental Science*:in press, 2026. doi: 10.1039/D6EE00129G.

[10] Justin C. Bui et al. Continuum Modeling of Porous Electrodes for Electrochemical Synthesis. *Chemical Reviews*, 122:11022–11084, 2022. doi: 10.1021/acs.chemrev.1c00901.

[11] Lien-Chun Weng, Alexis T Bell, and Adam Z Weber. Modeling gas-diffusion electrodes for CO_2 reduction. *Physical Chemistry Chemical Physics*, 20:16973–16984, 2018. doi: 10.1039/C8CP01319E.

[12] Venu Gopal Agarwal and Sophia Haussener. Quantifying mass transport limitations in a microfluidic CO_2 electrolyzer with a gas diffusion cathode. *Communications Chemistry*, 7:47, 2024. doi: 10.1038/s42004-024-01122-5.

[13] Omnia A. El-Shafie et al. Modeling and Numerical Investigation of the Performance of Gas Diffusion Electrodes for the Electrochemical Reduction of Carbon Dioxide to Methanol. *Industrial & Engineering Chemistry Research*, 59:20929–20942, 2020. doi: 10.1021/acs.iecr.0c02358.

[14] Robert D. Walker Jr. *A study of gas solubilities and transport properties in fuel cell electrolytes - First semiannual report*. Tech. rep. NASA-CR-74779. Accessed: 31st January 2026. NASA, 1966.

[15] R.J. Gilliam et al. A review of specific conductivities of potassium hydroxide solutions for various concentrations and temperatures. *International Journal of Hydrogen Energy*, 32:359–364, 2007. doi: 10.1016/j.ijhydene.2006.10.062.

[16] Pal M. Sipos, Glenn Hefter, and Peter M. May. Viscosities and Densities of Highly Concentrated Aqueous MOH Solutions ($M^+ = Na^+, K^+, Li^+, Cs^+, (CH_3)_4N^+$) at 25.0 °C. *Journal of Chemical & Engineering Data*, 45:613–617, 2000. doi: 10.1021/je000019h.

[17] C. R. Wilke. A Viscosity Equation for Gas Mixtures. *The Journal of Chemical Physics*, 18:517–519, 1950. doi: 10.1063/1.1747673.

[18] Edward N. Fuller, Paul D. Schettler, and J. Calvin. Giddings. NEW METHOD FOR PREDICTION OF BINARY GAS-PHASE DIFFUSION COEFFICIENTS. *Industrial & Engineering Chemistry*, 58:18–27, 1966. doi: 10.1021/ie50677a007.

[19] K.G. Schulz et al. Determination of the rate constants for the carbon dioxide to bicarbonate inter-conversion in pH-buffered seawater systems. *Marine Chemistry*, 100:53–65, 2006. doi: 10.1016/j.marchem.2005.11.001.

[20] Ziming Yang et al. Modeling and Upscaling Analysis of Gas Diffusion Electrode-Based Electrochemical Carbon Dioxide Reduction Systems. *ACS Sustainable Chemistry & Engineering*, 9(1):351–361, 2021. doi: 10.1021/acssuschemeng.0c07387.

[21] Yikai Chen, Nathan S. Lewis, and Chengxiang Xiang. Modeling and Simulation of the Spatial and Light-Intensity Dependence of Product Distributions in an Integrated Photoelectrochemical CO_2 Reduction System. *ACS Energy Letters*, 1:273–280, 2016. doi: 10.1021/acsenergylett.6b00134.

[22] N Gupta, M Gattrell, and B MacDougall. Calculation for the cathode surface concentrations in the electrochemical reduction of CO_2 in $KHCO_3$ solutions. *Journal of Applied Electrochemistry*, 36:161–172, 2006. doi: 10.1007/s10800-005-9058-y.

[23] William L. Marshall and E. U. Franck. Ion product of water substance, 0–1000 °C, 1–10,000 bars. New International Formulation and its background. *Journal of Physical and Chemical Reference Data*, 10:295–304, 1981. doi: 10.1063/1.555643.

[24] Walter G. Whitman. The two film theory of gas absorption. *International Journal of Heat and Mass Transfer*, 5:429–433, 1962. doi: 10.1016/0017-9310(62)90032-7.

[25] Stephanie Nitopi et al. Progress and Perspectives of Electrochemical CO_2 Reduction on Copper in Aqueous Electrolyte. *Chemical Reviews*, 119:7610–7672, 2019. doi: 10.1021/acs.chemrev.8b00705.

[26] Theresa Jaster et al. Electrochemical CO_2 reduction toward multicarbon alcohols - The microscopic world of catalysts & process conditions. *iScience*, 25(4):104010, 2022. doi: 10.1016/j.isci.2022.104010.

[27] Edmund J.F. Dickinson and Andrew J. Wain. The Butler-Volmer equation in electrochemical theory: Origins, value, and practical application. *Journal of Electroanalytical Chemistry*, 872:114145, 2020. doi: 10.1016/j.jelechem.2020.114145.

[28] Mohammad Bahreini et al. Investigation of CO_2 Reduction to Formate in an Industrial-Scale Electrochemical Cell through Transient Numerical Modeling. *Industrial & Engineering Chemistry Research*, 63(42):18187–18198, 2024. doi: 10.1021/acs.iecr.4c03239.

[29] Shiyuan Liu, Ce Zhang, and Wei Yao. 3D Modeling and Numerical Investigation of Electrochemical CO_2 Reduction in Microfluidic Flow Cells. *Energy Technology*, 11:2300119, 2023. doi: 10.1002/ente.202300119.

[30] Guyu Zhang et al. Scale-up study of electrochemical carbon dioxide reduction process through data-driven modelling. *Fuel*, 373:132400, 2024. doi: 10.1016/j.fuel.2024.132400.

[31] Junbo Fu et al. Simulation of the electrochemical reduction of CO2 to CO from a bicarbonate solution. *Energy & Environmental Science*, 2026. doi: 10.1039/D6EE03258C.

[32] Kunna Wu et al. Modeling and Experimental Validation of Electrochemical Reduction of CO_2 to CO in a Microfluidic Cell. *Journal of The Electrochemical Society*, 162(1):F23, 2015. doi: 10.1149/2.1021414jes.

[33] Yao Jiang et al. Parameter sensitivity analysis for CO_2 electrochemical reduction electrolyzer. *International Journal of Hydrogen Energy*, 100:1291–1300, 2025. doi: 10.1016/j.ijhydene.2024.12.174.

[34] Yosra Kotb et al. Modeling of a Microfluidic Electrochemical Cell for the Electro-Reduction of CO_2 to CH_3OH. *Journal of The Electrochemical Society*, 164:E391, 2017. doi: 10.1149/2.0741713jes.

[35] John O'M. Bockris, Amulya K.N. Reddy, and Maria E. Gamboa-Aldeco. *Modern Electrochemistry 2A: Fundamentals of Electrodics*. 2nd. New York, USA: Kluwer Academic Publishers, 2000.

[36] Sivagaminathan Balasubramanian and Adam Z. Weber. "Continuum, Macroscopic Modeling of Polymer-Electrolyte Fuel Cells". *Physical Multiscale Modeling and Numerical Simulation of Electrochemical Devices for Energy Conversion and Storage: From Theory to Engineering to Practice*. Ed. by Alejandro A. Franco, Marie Liesse Doublet, and Wolfgang G. Bessler. London, UK: Springer, 2016, 91–149.

[37] Jorge H.B. Sampaio. An iterative procedure for perpendicular offsets linear least squares fitting with extension to multiple linear regression. *Applied Mathematics and Computation*, 176:91–98, 2006. doi: 10.1016/j.amc.2005.09.054.

[38] Michael Faraday. IV. Experimental researches in electricity. - Third series. *Philosophical Transactions of the Royal Society of London*, 123:23–54, 1833. doi: 10.1098/rstl.1833.0006.

[39] Michael Faraday. VI. Experimental researches in electricity. - Seventh Series. *Philosophical Transactions of the Royal Society of London*, 124:77–122, 1834. doi: 10.1098/rstl.1834.0008.

[40] Chao Zhan et al. Key intermediates and Cu active sites for CO_2 electroreduction to ethylene and ethanol. *Nature Energy*, 9:1485–1496, 2024. doi: 10.1038/s41560-024-01633-4.
